# Molecular Plasmonics with Metamaterials

**DOI:** 10.1021/acs.chemrev.2c00333

**Published:** 2022-10-04

**Authors:** Pan Wang, Alexey V. Krasavin, Lufang Liu, Yunlu Jiang, Zhiyong Li, Xin Guo, Limin Tong, Anatoly V. Zayats

**Affiliations:** # State Key Laboratory of Modern Optical Instrumentation, College of Optical Science and Engineering, Zhejiang University, Hangzhou310027, China; ‡ Department of Physics and London Centre for Nanotechnology, King’s College London, Strand, LondonWC2R 2LS, U.K.; § Jiaxing Key Laboratory of Photonic Sensing & Intelligent Imaging, Jiaxing314000, China; ∥ Intelligent Optics & Photonics Research Center, Jiaxing Research Institute, Zhejiang University, Jiaxing314000, China

## Abstract

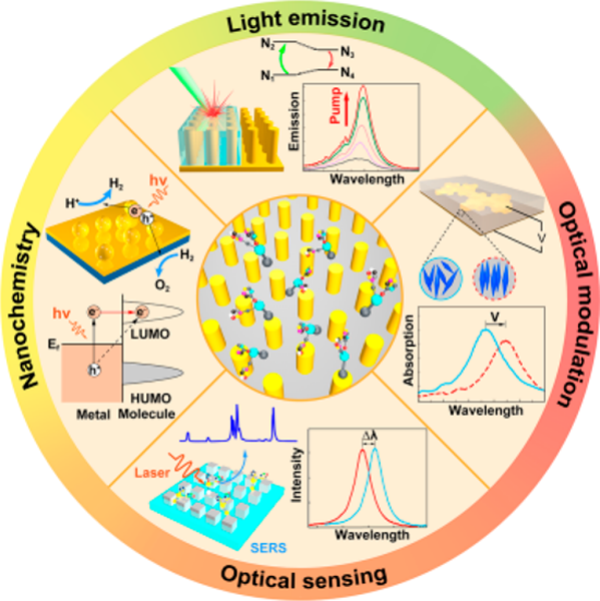

Molecular plasmonics, the area which deals with the interactions between surface plasmons and molecules, has received enormous interest in fundamental research and found numerous technological applications. Plasmonic metamaterials, which offer rich opportunities to control the light intensity, field polarization, and local density of electromagnetic states on subwavelength scales, provide a versatile platform to enhance and tune light-molecule interactions. A variety of applications, including spontaneous emission enhancement, optical modulation, optical sensing, and photoactuated nanochemistry, have been reported by exploiting molecular interactions with plasmonic metamaterials. In this paper, we provide a comprehensive overview of the developments of molecular plasmonics with metamaterials. After a brief introduction to the optical properties of plasmonic metamaterials and relevant fabrication approaches, we discuss light-molecule interactions in plasmonic metamaterials in both weak and strong coupling regimes. We then highlight the exploitation of molecules in metamaterials for applications ranging from emission control and optical modulation to optical sensing. The role of hot carriers generated in metamaterials for nanochemistry is also discussed. Perspectives on the future development of molecular plasmonics with metamaterials conclude the review. The use of molecules in combination with designer metamaterials provides a rich playground both to actively control metamaterials using molecular interactions and, in turn, to use metamaterials to control molecular processes.

## 1 Introduction

Interaction between light and molecules manifests itself as absorption, fluorescence, elastic and inelastic scattering, nonlinear optical processes, and photochemical transformations, to name but a few, and lays the foundation for the development of a wide range of technological applications, including generation and modulation of light, analysis, detection and identification of molecules, photocatalysis, photoelectrochemistry, and many others. However, due to the dramatic mismatch between the wavelength of light (typically 100s of nm) and the size of molecules (typically less than 10 nm), their interaction strength is extremely low. Various kinds of dielectric, semiconductor, and metallic nanostructures, acting as nanoantennas or metamaterials were proposed to enhance light-molecule interactions.^1,2^ An advantage of dielectric nanostructures is in their low loss and straightforward access to multipolar resonances, which gives the opportunity to influence molecular processes requiring higher-order symmetries. Nevertheless, plasmonic metallic nanostructures offer a richer playground for investigations and applications of molecular optical properties, providing extreme electromagnetic field localization and enhancement at the nanoscale together with other useful effects, associated with free-electron dynamics and, counterintuitively, losses.

Metallic nanostructures support surface plasmons, which are collective oscillations of free carriers at the interface between a conductor and a dielectric coupled to an electromagnetic field, manifesting themselves either in the form of surface plasmon polaritons (SPPs) propagating at extended conductor/dielectric interfaces or as localized surface plasmons (LSPs) in confined geometries.^3−5^ They have an intrinsic ability to localize the electromagnetic fields down to deep-subwavelength scales and increase local field intensity, resulting in greatly enhanced light-matter interactions.^6−8^ Therefore, they have opened up a new realm of possibilities for a variety of applications ranging from subdiffraction waveguiding,^9−11^ biochemical sensing,^12,13^ and optical modulators^14−17^ to nonlinear optics^18,19^ and nanolasers.^20−23^ In the past decades, benefiting from the advances in chemistry and nanofabrication, plasmonic nanostructures of different materials and shapes^24−32^ have been developed to achieve an engineered optical response and optimize the field localization and enhancement for a variety of applications.

Plasmonic metamaterials, consisting of periodically or randomly arranged plasmonic nanostructures (also called meta-atoms) with the size and spacing much smaller than the wavelength of interest, have been widely investigated to further enable the engineering of active functionalities and optical performance.^33−36^ In such artificially structured materials, the macroscopic optical properties are predominantly determined by the size, shape, and spacing of the meta-atoms of the metamaterials, in addition to the optical properties of the constituent materials of the meta-atoms. Using different meta-atom designs (e.g., split-ring resonators (SRRs), nanorods, nanospirals, and other shapes), the optical response of metamaterials can be engineered with unprecedented degrees of freedom to demonstrate exotic properties such as extremely low-frequency plasmons,^37^ artificial magnetism,^38,39^ negative refractive index,^40−42^ hyperbolic dispersion,^35,43^ enhanced nonlinear optical response,^15,44^ strong optical chirality,^45,46^ and enhanced optomechanic effects.^47,48^ Accordingly, applications of plasmonic metamaterials in various fields have been successfully demonstrated, including optical waveguiding, super-resolution imaging, ultrasensitive optical sensing, electromagnetic cloaking, nonlinear optical devices, and others.

Plasmonic metamaterials have large surface areas where molecules can be absorbed simultaneously providing a deep-subwavelength confinement of electromagnetic fields. Consequently, intense optical fields can be engineered at the required locations at the surface where molecules are placed. Apart from affecting the molecules directly, these fields generate energetic hot carriers through the nonradiative decay of surface plasmons^49^ as well as introduce heat due to thermal effects, influencing light-molecule interactions. Therefore, plasmonic metamaterials provide an attractive platform for the investigation and exploitation of molecular plasmonics, which have received ever-increasing research interest in the past decades.

In general, the light-molecule interactions in plasmonic metamaterials can be classified into two broad categories, which we call passive and active. In the former case, due to the strong dependence of the optical responses of plasmonic metamaterials on their local dielectric environments, the mere presence of molecules in the near-field of plasmonic meta-atoms introduces a substantial modulation of the optical properties of the metamaterials. In the latter case, the strong local fields can greatly enhance and modulate the optical properties of neighboring molecules such as absorption, fluorescence, Raman scattering cross sections, and the nonlinear response, while chemical reactivity of molecules can be activated by optically-generated hot carriers. Through understanding and control of the molecular plasmonic interactions, efficient approaches for exciting, manipulating, sensing, and analyzing molecules have been developed. These advances have in turn led to sensitive analytical tools and advanced nanophotonic devices for a variety of applications, such as ultrasensitive optical sensing, surface-plasmon-enhanced Raman spectroscopy, plasmon-enhanced fluorescence, nanoscale lasers, ultrafast optical modulation, and plasmon-assisted nanochemistry.

In this review, we focus on the developments in molecular plasmonics with metamaterials. We start with an introduction of the optical properties of plasmonic metamaterials in section 2, followed by a brief overview of the approaches for fabrication of plasmonic metamaterials and incorporation of molecules in section 3. In section 4, we discuss light-molecule interactions in plasmonic metamaterials in the weak and strong coupling regimes. Section 5 covers optical phenomena in plasmonic metamaterials functionalized with molecular gain media. Modulation of optical signals with molecular plasmonic metamaterials via various control mechanisms is overviewed in section 6. Section 7 is focused on the application of plasmonic metamaterials in optical sensing, ranging from biochemical sensing to surface-enhanced spectroscopies to chiral sensing. The exploitation of hot carriers generated in plasmonic metamaterials for nanochemistry is introduced in Section 8. Section 9 concludes this review with perspectives on the future development of molecular plasmonics with metamaterials.

## 2 Optical Properties of Plasmonic Metamaterials

Metamaterials provide a unique opportunity to create artificial optical media with engineered and frequently exotic optical properties beyond those present in nature. In this section, we overview the fundamentals of the metamaterials and discuss the metamaterial designs important in the context of engineering their interaction with molecules.

### 2.1 General Definition of Plasmonic Metamaterials

**Figure 1:**
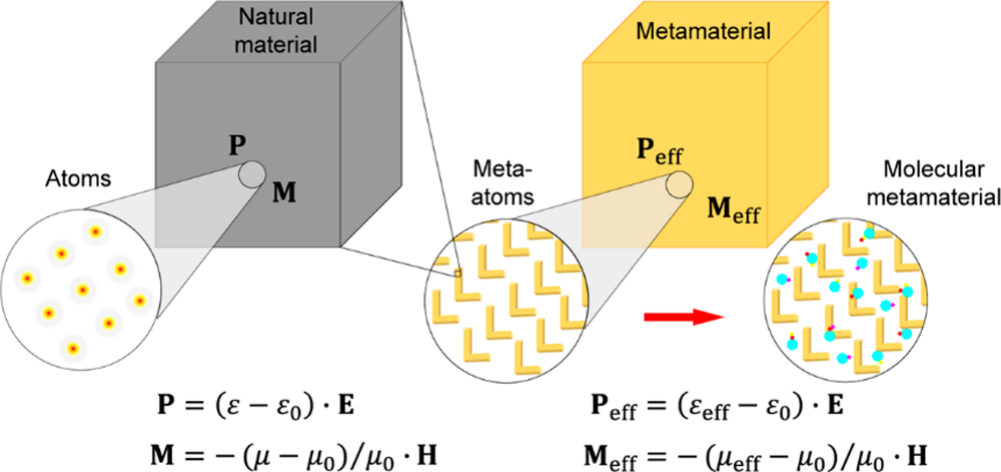
Schematics showing a transformation from natural materials to plasmonic metamaterials and further to molecular plasmonic metamaterials.

The development of nanotechnology enabled a revolutionary step in structuring matter at the nanoscale and creating optical resonators with subwavelength sizes. In this respect, metallic materials present a particular interest, offering very pronounced optical characteristics, underlined by dynamics of the free electron gas resulting in large material polarization. Nanostructured metallic objects possess an enhanced optical response through the support of LSPs, presenting resonant oscillations of the free-electron gas in a bounded geometry. By controlling the nanoparticle shapes using the nanostructuring or chemical fabrication methods, it is possible to engineer their optical behavior and, therefore, realize the so-called meta-atoms with a designed (e.g., resonant) optical response, going beyond the predefined optical properties of atoms given by nature. Taking one step further and arranging such meta-atoms in regular subwavelength arrays or random distributions (both producing no diffractive orders) leads to the realization of artificial nanostructured optical materials, metamaterials, with engineered optical properties, breaking through constraints set by ordinary optical materials on available permittivities and permeabilities (Figure 1). These properties are defined by both the material, shape, and size of the meta-atoms and their near-field electromagnetic coupling in the assembly.

Two-dimensional analogues of metamaterials, produced by 2D arrays of meta-atoms (in other words, thin films nanostructured on a subwavelength scale), are usually called metasurfaces. There are various definitions of metamaterials and metasurfaces, as wide as including the diffraction-related (photonic crystal) effects^50^ or as narrow as assuming a certain application, e.g., a phase control on a subwavelength scale.^51^ Here, we adhere to the most natural, in our view, definition: an optical metamaterial is an artificially structured medium, whose optical properties can be described by an effective medium theory (EMT) providing an effective permittivity and permeability (Figure 1); in case of the metasurface, one can speak about an effective surface impedance. As follows from this definition, the nanostructured media producing diffraction orders fall beyond the metamaterial realm. At the same time, within this definition of a metamaterial, the size of the meta-atoms may generally be larger than the light wavelength if at least one dimension is subwavelength. The derivation of an EMT is not always an easy task; for the same metamaterial, an EMT can describe well one class of optical phenomena and be marginally applicable to another. Thus, a nanostructured medium can behave as a metamaterial for one class of optical phenomena but require a much more complex treatment taking into account the actual nanostructuring for others. For example, light transmission through vertically oriented nanorod arrays is characterized by an EMT very well, and even better by its nonlocal extension,^52^ but for the description of molecular emission, the implementation of the nonlocal EMT is a must,^53^ and even in this case, the EMT description is not perfect. The latter is underlined by the complexity of the emission process due to the involvement of the emitter near fields possessing large wave vector components, breaking EMT as the associated wavelengths become comparable with the meta-atom spacing, which leads to the diffractive effects. Additionally, emission quenching related to the absorption of the near-field components of the emitter makes the emission process position-dependent, which produces difficulties for the introduction of homogeneous optical constants. For more details on this, see subsections 2.2, 4.2, and 4.3.

### 2.2 Metamaterials with Hyperbolic Dispersion

**Figure 2:**
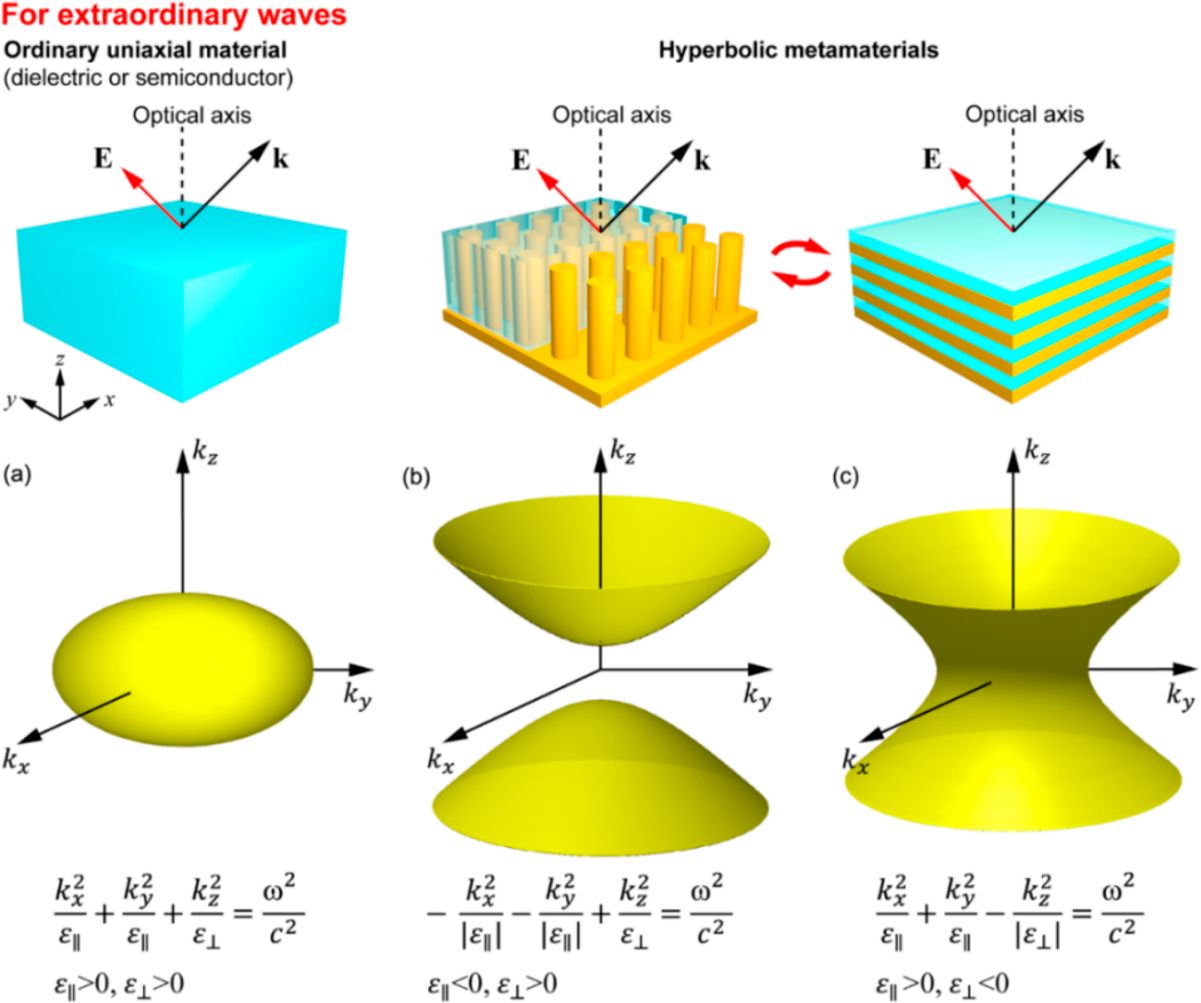
Transformation of the dispersion of extraordinary waves from (a) elliptical for natural uniaxial optical materials to hyperbolic for plasmonic metamaterials of (b) Type I and (c) Type II in a lossless case. Both Type I and Type II metamaterials can be realized with either nanowire or multilayer structures shown at the top.

Due to the vectorial nature of the electromagnetic field, the propagation of light in an anisotropic medium depends on both its propagation direction and polarization. In the general case, for any propagation direction there are two linearly polarized eigenwaves, which keep their state unchanged during the propagation but have different refractive indices *n* given by the solution of Fresnel’s equation of wave normals:^54^
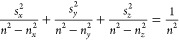
1where (*s*_*x*_, *s*_*y*_, *s*_*z*_) is the unit vector along the direction of the wave vector **k** and , , and  are the refractive indices along the three optical axes of the material (the permittivity tensor of any material can be diagonalized in the absence of chiral or gyrotropic contributions). The situation is simplified for uniaxial optical materials (here we consider dielectric or semiconductor materials with negligible losses, which are relevant to the discussion) having a selected axis with a refractive index *n*_e_ = *n*_∥_ (called the optical axis), while the refractive indexes for the other two directions are equal: *n*_0_ = *n*_⊥_ (subscripts mark the angle between the considered direction and the optical axis). In this case, the electromagnetic waves propagating in the material are divided into two types: ordinary, having polarization perpendicular to the optical axis and experiencing the refractive index *n*_0_ independently of the direction of their wave vector:
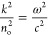
2and extraordinary with the propagation constant dependent on the propagation direction, so that their wave vectors are represented by an ellipsoid in the *k*-space (Figure 2a):
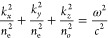
3

There are two simple examples of uniaxial plasmonic metamaterials. One of them is a nanorod metamaterial produced by an array of vertically oriented metallic nanowires (diameter ∼20–50 nm, spacing ∼50–100 nm, length ∼20–500 nm), usually fabricated using electrodeposition into a pored dielectric matrix (see subsection 3.3.1). The other is a multilayer metamaterial, produced by a stack of alternating dielectric and metallic layers with typical thicknesses ∼10–30 nm, fabricated using standard thin-layer deposition techniques. Using EMTs based on averaging the electric field vectors **E** and **D** over the volume, one can derive the effective permittivities of both nanorod and multilayer metamaterials (the effective permeabilities will be 1 due to the absence of either a natural or artificial magnetic response). For the nanorod metamaterial this gives^55,56^
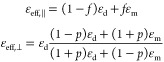
4where ε_d_ and ε_m_ are the permittivities of the dielectric and metal, respectively, while *f* = *πd*^2^/(4*p*^2^ sin(π/3)) is the metal filling factor calculated in this case of a hexagonal array, *d* is the nanowire diameter, and *p* is the array periodicity. For the multilayer hyperbolic metamaterial, the EMT results in
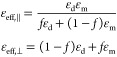
5where *f* = *t*_m_/(*t*_d_ + *t*_m_) is the metal filling factor calculated for the structure with thicknesses *t*_d_ and *t*_m_ for the dielectric and metal, respectively.^57^ Balancing the frequency-dependent permittivities of the dielectric (positive) and metal (negative) together with the geometrical parameters of the structure, it is possible to achieve a situation when the real part of ε_∥_ and ε_⊥_ have the opposite signs (at the first step, a lossless case is considered). With such a seemingly simple modification, the changes to the metamaterial dispersions are dramatic. The dispersions of the extraordinary (TM-polarized) waves change their shape from a confined ellipse to an unbounded hyperboloid (Figure 2). Both type I (ε_∥_ < 0, ε_⊥_ > 0, Figure 2b) and type II (ε_∥_ > 0, ε_⊥_ < 0, Figure 2c) hyperbolic dispersions are possible to realize with either nanowire or multilayer designs depending on the chosen material/geometrical parameters (Figure 2b and c). Thus, as it follows from the dispersions, the metamaterial starts to support optical modes with arbitrarily large wave vectors located in the vicinity of the cones defined by the hyperboloid asymptotes. Of course, these conclusions are based on the derivation of optical parameters with the use of the metamaterial homogenization approach and EMTs which rely on certain assumptions. Particularly, the nanostructuring is assumed to be subwavelength, which is legitimate only for wave vectors of the metamaterial modes smaller than ∼1/*a*, where *a* is the period of the nanostructuring. This limits the validity of the theory and its results in the *k*-space.^35^ Furthermore, the unavoidable presence of losses in the metal will transform the dispersion hyperbolas into finite hyperbola-like surfaces.^58^

The local EMT presented above describes well all the major optical properties of both nanorod and multilayer metamaterials, but for their detailed characterization, nonlocality (spatial dispersion) originating from the metamaterial nanostructuring should be considered.^15,52,53,59−61^ We note that this nonlocality is introduced purely by the nanostructuring and is different from the nonlocality originating from the plasmonic material response due to a complex free electron dynamics.^62,63^ In particular, the consideration of the nonlocal response in nanorod metamaterials results in values of the extinction which are closer to the experiment. The spatial dispersion results in the appearance of two TM-polarized modes in the metamaterial with drastically different refractive indices; the coupling efficiency of light to one or the other mode depends on the angle of incidence and manifests itself in the splitting of the angular-dependent peak in the extinction. The nonlocal version of the effective medium description can be developed by considering the interaction of the cylindrical surface plasmons, instead of the dipolar LSP modes, excited on the nanorods of the metamaterial.^56^ It should be noted that for the numerical simulations of the actual metamaterial structure, these nonlocal effects are automatically taken into account. The nonlocal response is particularly important for the description of nonlinear effects and molecular emission inside the metamaterial, where it plays a crucial role^53^ (see subsection 4.3). The effect of nonlocality was also observed for the multilayer metamaterials.^59,60^

**Figure 3:**
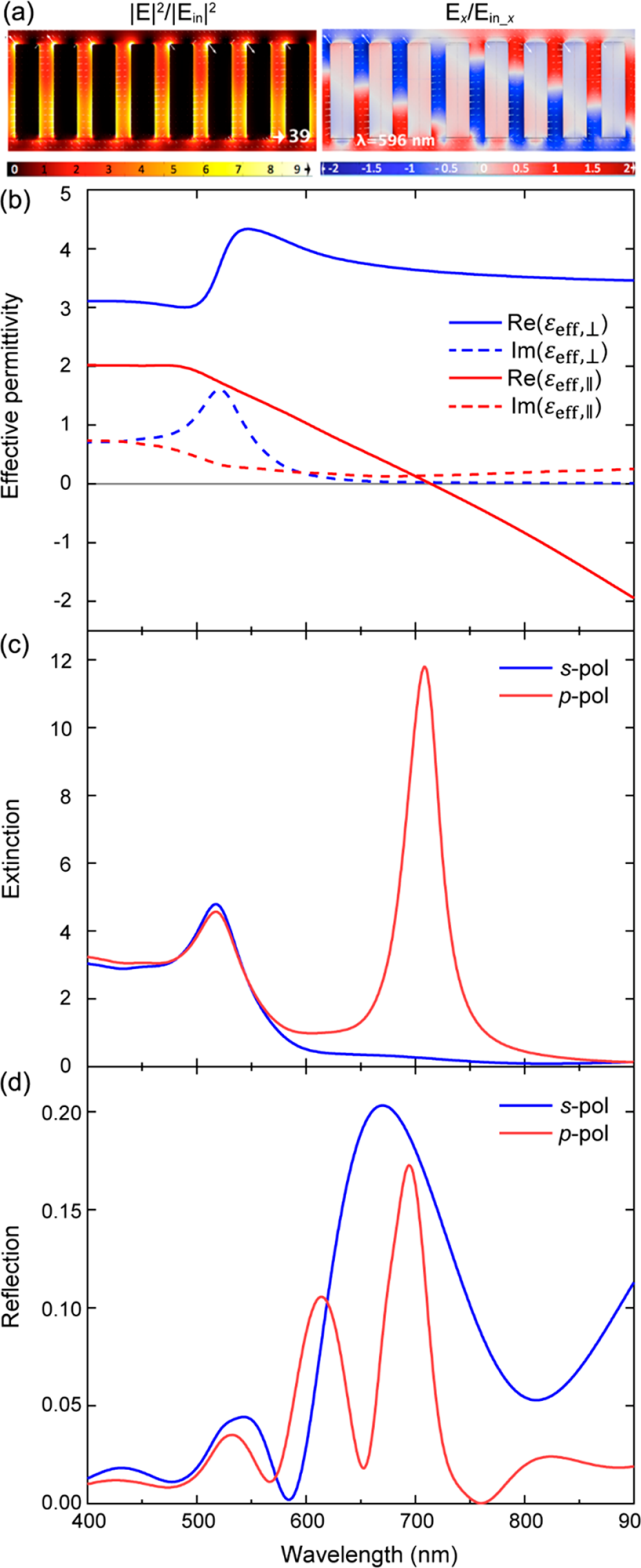
Optical response of a gold nanorod metamaterial. (a) Field maps of intensity and *E*_*x*_ component of the electric field inside the nanorod metamaterial in the hyperbolic dispersion regime excited at oblique light incidence, illustrating electromagnetic coupling between the nanorods in the metamaterial. Reproduced with permission from ref (72), Copyright 2019 IOP Publishing Ltd. (b) Real and imaginary parts of the effective permittivity of a plasmonic nanorod metamaterial (gold nanorods in an alumina matrix in a hexagonal array with periodicity 80 nm and diameter 30 nm) calculated using a local EMT. (c) Extinction: −ln(*T*), where *T* is the transmission, and (d) reflectivity of a metamaterial layer with a thickness of 450 nm and complex anisotropic permittivity as in panel b.

As an illustrative example, we discuss here the optical response of the nanorod metamaterials (Figure 3a). Using a conventional (local) version of an EMT presented above, one can calculate the tensor components of the anisotropic permittivity (Figure 3b). One can see that for chosen geometrical parameters and materials, the real parts of the permittivity components perpendicular to the optical axis are always positive, while the corresponding component along the optical axis changes sign at the wavelength around 700 nm. The spectral interval around this wavelength is called an epsilon-near-zero (ENZ) region; it is important for many applications, some of which will be discussed below. No less importantly, the ENZ wavelength divides the optical response of the metamaterial into elliptical (short-wavelength) and hyperbolic (long-wavelength) regions (bear in mind a breakdown of the EMT for a perfectly periodic structure when the spacing between the nanorods becomes comparable with the propagating mode wavelength, following which diffraction effects appear). For metals with lower losses, the double-bend in the spectral dependence of Re[ε_eff,⊥_(ω)] can have a larger amplitude, going below zero and thus resulting in two ENZ points for ε_eff,⊥_ and the other type of hyperbolic dispersion in the spectral interval between them. When one calculates (or measures) the transmission through a nanorod metamaterial layer, two extinction peaks are observed (Figure 3c). The short-wavelength peak corresponds to the Re[ε_eff,⊥_(ω)] → ∞ condition (in the lossless case), and the long-wavelength one, to the ENZ point Re[ε_eff,∥_(ω)] → 0. The peak related to ε_eff,⊥_ occurs for both *s*- and *p*-polarized waves (as they both have an electric field component perpendicular to the optical axis). The long-wavelength peak is observed only for *p*-polarized illumination with an electric field component along the optical axis and, therefore, perceiving ε_∥_.

In the microscopic description, the origin of these peaks can be traced to the plasmonic properties of individual meta-atoms. The short-wavelength peak originates from a dipolar transverse resonance of the individual metallic nanorods, shifted due to the near-field coupling between the nanorods in the metamaterial. This can be seen by separating the rods in the array (*f* → 0) which leads to recovering the resonance condition for the transverse dipolar LSP of individual nanorods ε_m_(ω) = −ε_d_. The characteristic double-bend in the real part of the transverse effective permittivity corresponds to the peak in its imaginary part (due to absorption), as expected for for a resonant response (Figure 3b). The long-wavelength peak in the ENZ spectral range (Figure 3b and c) is related to the purely collective response of the plasmonic nanorods, determined by the interacting cylindrical surface plasmons, which leads to only nonpropagating (evanescent) solutions for electromagnetic modes inside the metamaterial, resulting in the increased extinction,^52^ similar to natural ENZ materials.^64^ In the ENZ range, the optical response of the metamaterial is very sensitive to the change of optical properties of the constituting media. This phenomenon was used for the demonstration of ultrafast all-optical switching^15,65^ and polarization control (via the modulated anisotropy of the metamaterial)^44^ based on femtosecond-scale changes of the metal permittivity related to the optically-induced variation of the energy distribution of the free electron gas. The ENZ regime can also lead to the enhancement of second harmonic generation (SHG) if the fundamental frequency is in the ENZ spectral range.^66^ The microscopic origin of the enhanced SHG in this case is a surface nonlinearity of the nanorods, made from a centrosymmetric metallic material.^62^

The two extinction peaks considered above define the spectral regions where the metamaterial is opaque. At the same time, hyperbolic metamaterial slabs also support Fabry–Perot and guided modes, the spectral features of which are barely resolved in extinction spectra, overshadowed by the large magnitude of the extinction peaks, but are observed as minima or maxima (depending on the particular geometrical parameters of the sample) in transmission and, particularly, reflection (Figure 3d). The metamaterial slab guided modes can be used for engineering molecular emission^67^ (see subsection 4.3) or enhancement of second harmonic generation.^68^ A two-dimensional analogue of hyperbolic metamaterials for SPP waves–hyperbolic metasurfaces–has also been realized.^69,70^

Overall, the effect created by the metamaterial hyperbolicity can be very pronounced and, being proven experimentally, found applications in subwavelength microscopy and lithography, thermal and spontaneous emission control, ultrafast optical modulation, lasing, and sensing,^35,71^ many of which will be discussed below in the case of metamaterials functionalized with molecules.

### 2.3 Chiral Metamaterials

**Figure 4:**
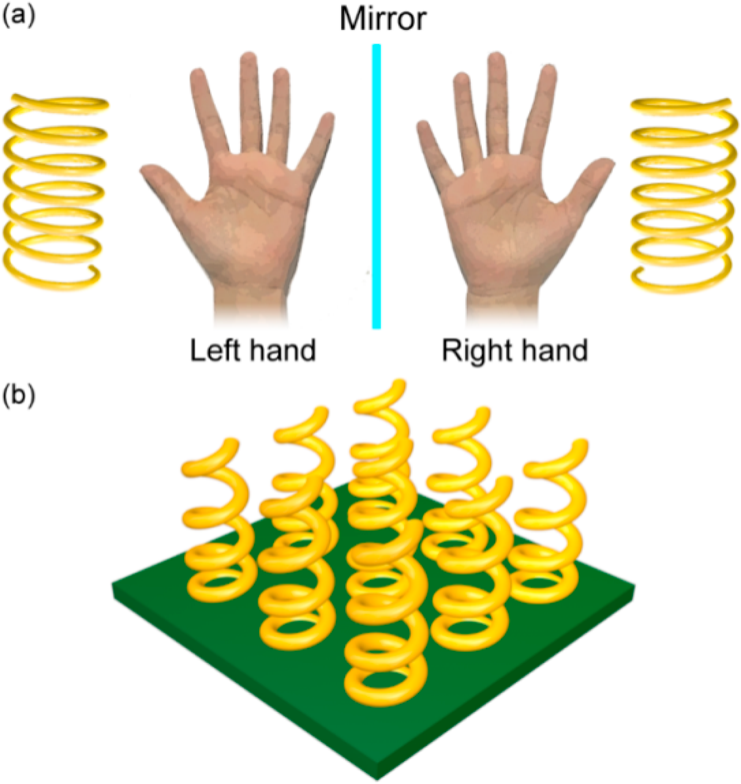
Schematic illustration of a chiral metamaterial. (a) Concept of chirality. (b) One of the possible designs of chiral plasmonic metamaterials, fabricated using, e.g., a direct laser writing in a positive photoresist followed by metal electrodeposition in the obtained helical channels.

A special type of metamaterials important for conditioning molecular interactions is a chiral metamaterial. According to the definition of chirality, an object is said to be chiral when it cannot be superimposed with its mirror image by any combination of translational and rotational operations. Some of the classical examples would be left and right hands (indeed, the term chirality comes from the Greek “χειρ”, meaning “hand”) or an ordinary elastic spring (Figure 4a). Chirality is widespread in nature, across various areas, from the spin of elementary particles to the configuration of chemical molecules. In electromagnetism, left and right circular polarizations (LCP and RCP) are two fundamental chiral states of light. Optical materials can also be chiral, resulting in different optical properties for circularly polarized light with different handedness. Particularly, this gives rise to optical birefringence, the effect when LCP and RCP light propagates through the medium with different propagation constants (which is related to different real parts of their refractive indices), and circular dichroism (CD), when LCP and RCP light states are differently absorbed (which is related to the different imaginary parts of their refractive indices). Together, these two effects are referred to as the optical activity of the medium. Phenomenologically, the chirality of the material means that its electric and magnetic responses are coupled:
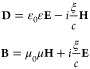
6where ξ is the chirality parameter. As the magnetic response of natural materials at optical frequencies is very weak, their chiral optical properties are weak as well: light has to travel macroscopic distances through the media to achieve pronounced optical activity effects.

As metamaterials provide the exact technology to enhance both electric and magnetic responses of the media, particularly through the exploitation of resonant effects, they offer an ideal platform for the realization of optical materials with greatly enhanced chiral properties. In this respect, a wide variety of chiral metamaterial designs has been implemented: an array of spirally shaped metallic nanowires would be a classical example (Figure 4b). Following this, artificial optical materials have been demonstrated with extremely enhanced CD and birefringence, which exceed by many orders of magnitude those of the natural materials. Consequently, chiral metamaterials have found applications in enhancing chiral sensing, which we will consider in subsection 7.5.

## 3 Fabrication of Plasmonic Metamaterials

Plasmonic metamaterials operating at optical wavelengths usually contain precisely shaped meta-atoms with subwavelength sizes and separations to ensure effective-medium-like behavior. While their arrangement does not need to be periodic, often periodicity is favorable due to fabrication considerations. Therefore, the fabrication of plasmonic metamaterials operating in the optical and near-infrared spectral ranges requires state-of-the-art fabrication processes with nanometer-scale resolution. In this section, we highlight some typical nanofabrication techniques that are widely used for the fabrication of plasmonic metamaterials. In general, top-down approaches, which start from continuous materials and sculpture the nanostructures, are sequential, and the overall size of the fabricated metamaterials is relatively small, while bottom-up approaches based on self-assembly provide an opportunity for large-scale fabrication of metamaterials.

### 3.1 Top-Down Lithography Approaches

#### 3.1.1 Electron Beam Lithography

**Figure 5:**
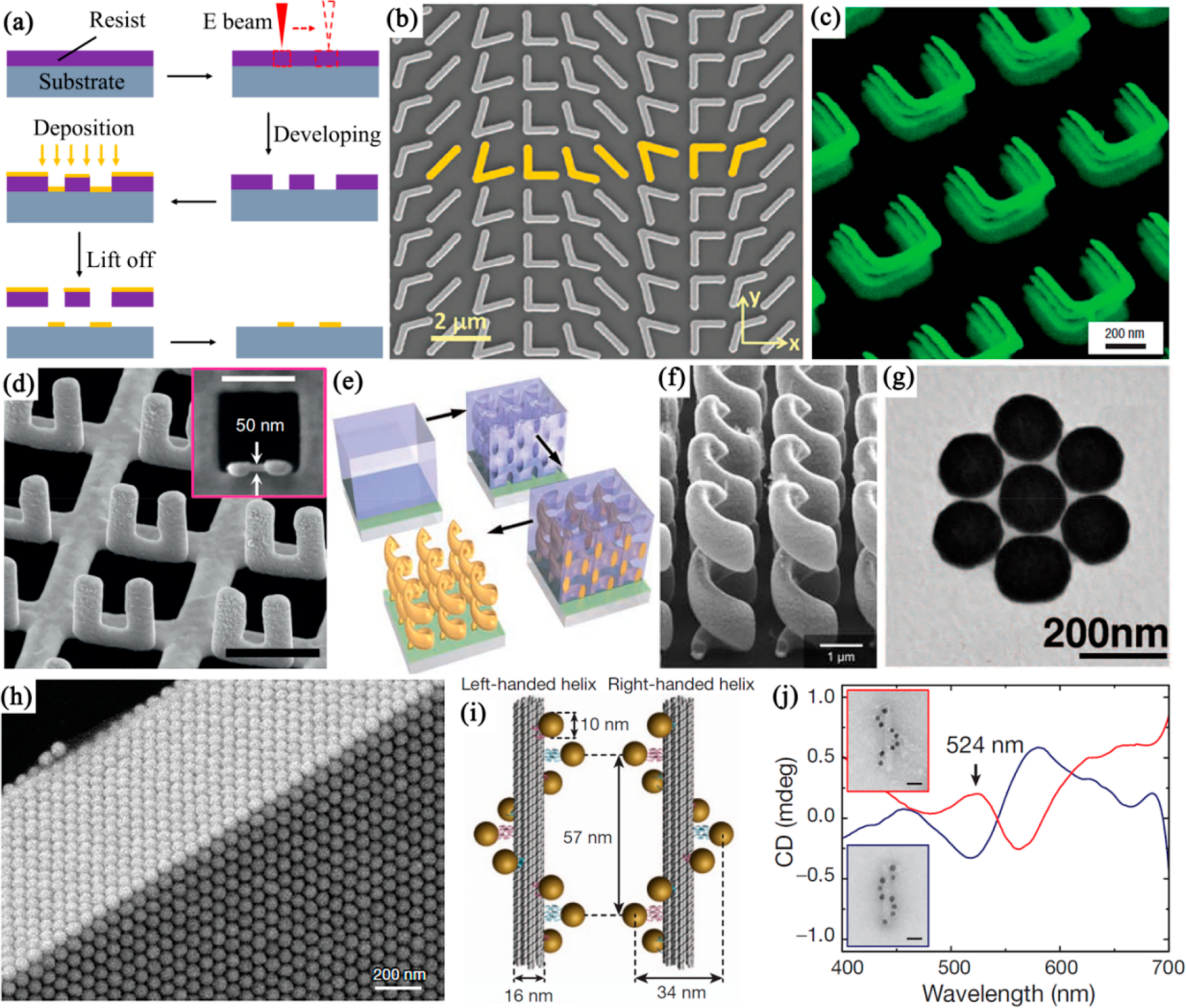
Fabrication approaches for plasmonic metamaterials. (a) Schematic illustration of the fabrication of metamaterials with EBL. (b) SEM image of a gold V-shaped antenna array fabricated on a silicon wafer. Reproduced with permission from ref (73). Copyright 2011 American Association for the Advancement of Science. (c) A four-layer SRR metamaterial. Reproduced with permission from ref (74). Copyright 2008 Springer Nature. (d) SEM images of a 3D U-shaped SRR array, with the top view of a metallic hole vertical SRR shown in the inset. Reproduced with permission from ref (77). Copyright 2015 Springer Nature. (e) Schematic illustration of the procedure for the fabrication of a square array of free-standing 3D gold helices and (f) a left-handed helix structure after removal of the polymer. Panels e and f are reproduced with permission from ref (45). Copyright 2009 American Association for the Advancement of Science. (g) TEM image of a heptamer self-assembled from gold nanoshells. Reprinted with permission from ref (78). Copyright 2010 The American Association for the Advancement of Science. (h) Edge view of a 3D crystal formed by nanospheres showing two (111) faces of a face-centered cubic crystal. Reproduced with permission from ref (79). Copyright 2020 Springer Nature. (i) Left- and right-handed nanohelices (diameter 34 nm, helical pitch 57 nm) formed by nine gold nanoparticles (10 nm in diameter) that are attached to the surface of DNA origami with 24-helix bundles (16 nm in diameter). (j) CD spectra of left-handed (red line) and right-handed (blue line) nanohelices in panel i. Insets: corresponding TEM images of left- and right-handed nanohelices (scale bars, 20 nm). Panels i and j are reproduced with permission from ref (80). Copyright 2012 Springer Nature.

Conventional photolithography has been widely used in the semiconductor and microfabrication industries. However, its spatial resolution is limited by the diffraction of light, making it unsuitable for the fabrication of plasmonic metamaterials with subwavelength-scale feature sizes for operation in visible spectral range. By using high-energy electrons with an extremely small wavelength (the de Broglie wavelength is around 0.01 nm) instead of light to expose the resist, electron beam lithography (EBL) provides a high spatial resolution on the order of 10 nm and has been widely used for the fabrication of metamaterials. As schematically shown in Figure 5a, in a typical EBL-based fabrication procedure, an electron beam is first focused to a spon size of several nanometers onto a substrate coated with an electron-beam resist to write a predesigned pattern. Subsequently, depending on the type of resist (positive or negative) used, the exposed (positive) or unexposed (negative) part of the resist is etched away with a chemical developer to form a patterned resist film. Finally, a thin layer of metal film is deposited onto the structure followed by a lift-off process to obtain a plasmonic metamaterial. In many cases, such a metamaterial is purely two-dimensional, i.e., a nanostructured surface, what is often called a metasurface. A typical plasmonic metasurface fabricated by EBL is shown in Figure 5b. Its unit cell (yellow) comprises eight gold V-antennas with width and thickness of ∼220 and 50 nm, respectively,^73^ showing the excellent control of the shape and size of each meta-atom with EBL.

Instead of using a single metal film, multilayered metal-dielectric metamaterials, such as hyperbolic metamaterial nanostructures,^43^ can be fabricated by depositing alternating metal and dielectric layers onto the patterned substrate. Plasmonic metamaterials with a 3D structure can also be fabricated by multistep EBL.^74−76^ The structure in each stacked layer as well as the lateral alignment and stacking distance between each layer can be precisely controlled during the fabrication, providing great opportunities for the development of metamaterials with exotic optical properties. Figure 5c shows an oblique view of a four-layer SRR structure,^74^ in which the underlying SRRs are clearly visible.

EBL is a powerful technique to fabricate complex plasmonic metamaterials with high spatial resolution. However, it is time-consuming due to the serial point-by-point scanning of the focused electron beam, which greatly limits its throughput and, thus, makes it rather problematic for the fabrication of large-scale metamaterials.

#### 3.1.2 Focused Ion Beam Lithography

Focused ion beam (FIB) milling uses a focused beam of ions (e.g., gallium, helium, neon) to sputter atoms away from a sample surface to form a desired nanostructure. Therefore, different from the EBL-based fabrication approach, which requires several processing steps and time-consuming optimization of lithography parameters, FIB lithography can directly mill a pattern onto a target substrate to form nanostructures,^81^ making it an attractive choice for the rapid prototyping of plasmonic metamaterials.^82−84^ In addition to the fabrication of planar plasmonic metamaterials, when combined with in situ irradiation-induced folding of metallic thin film structures, three-dimensional plasmonic metamaterials can be directly fabricated with FIB lithography.^77,85−87^Figure 5d shows an SEM image of a 3D U-shaped SRR array with each meta-atom composed of a vertical SRR placed at the edge of a hole in the metal film,^77^ which was fabricated with this approach.

Gallium-based FIB lithography has a resolution typically limited to 10 nm and provides undesired Ga-ion contamination of nanostructures, changing their optical properties. Helium- or neon-based FIBs have a much better fabrication resolution as low as 5 nm and greatly reduced contamination,^88^ which are extremely attractive for fabricating high-precision plasmonic metamaterials. However, they come at the cost of a significantly reduced milling speed due to the lighter ion mass in comparison with gallium ions. As in the case of the EBL-based fabrication technique, throughput and large-scale fabrication are the main challenges for the fabrication of plasmonic metamaterials with FIB lithography.

#### 3.1.3 Direct Laser Writing

With direct laser writing (DLW), nanopatterns can be directly written into a photoresist by nonlinear optical absorption-based polymerization. It is a versatile technique for fabrication of complex three-dimensional polymer micro-/nanostructures with submicron resolution.^89,90^ Combining DLW with the electrochemical metal deposition methods to metallize the dielectric framework, plasmonic metamaterials with complex structures can be readily fabricated.^45,91^ For example, Figure 5e shows a schematic illustration of the fabrication of a chiral metamaterial with DLW.^45^ First, a positive photoresist with an appropriate thicknessis spin-coated onto a conductive substrate. Second, a laser beam is tightly focused into the photoresist to write a pattern via two-photon polymerization, which is subsequently followed by the development to remove the polymer in the exposed regions. Third, the obtained voids are filled with metal using electroplating or other methods. Finally, the polymer template is completely removed by oxygen-plasma etching. Figure 5f shows an oblique-view SEM image of an as-fabricated left-handed gold-helix metamaterial, which has a fairly small surface roughness and works as a broad-band circular polarizer. To further improve the fabrication resolution of DLW, the combination of the stimulated-emission depletion microscopy technique and DLW has been introduced, which can reach a resolution limit as low as ∼50 nm.^92−95^

### 3.2 Bottom-Up Self-Assembly Approaches

In addition to the commonly used top-down fabrication approaches, plasmonic metamaterials can also be fabricated by a bottom-up self-assembly approach, which can build complex nanostructures directly in/from a solution from an ensemble of simple building blocks (i.e., plasmonic nanoparticles). The driving force for the assembly is originated from the mutual interactions (e.g., van der Waals forces, electrostatic forces, capillary forces, molecular binding forces) between nanoparticles as well as the interactions between nanoparticles and functional materials (e.g., polyelectrolytes, DNA). During the past decades, high-quality metallic nanoparticles with a variety of geometries (e.g., sphere, rod, triangle, cube) have been synthesized, providing a rich toolkit of building blocks for the construction of complex plasmonic metamaterials.

Evaporation of solutions containing plasmonic nanoparticles is a simple method to self-assemble nanoparticles into plasmonic metamaterials.^78,79,96−98^ As shown in Figure 5g, by slowly drying a droplet of plasmonic nanoshell (coated with a polymer monolayer) solution on a hydrophobic substrate, close-packed clusters of nanoshells such as trimmers and heptamers can be obtained. Such nanostructures exhibit pronounced magnetic and Fano-like resonances due to the strong near-field coupling.^78^ Their optical response is highly dependent on the interparticle spacing, which is defined by the chain length of the polymer and can be tuned with subnanometer precision.

Apart from the formation of nanostructures with short-range ordering, large-area self-assembled plasmonic metamaterials can also be fabricated with this method. For example, by evaporating a droplet of toluene containing polystyrene-stabilized gold nanoparticles on the interface of diethylene glycol, the nanoparticles can be assembled into densely packed face-centered cubic crystals with small gaps (∼1–4 nm) and excellent three-dimensional order (Figure 5h). Such nanoparticle arrangements support plasmon polaritons in the so-called “deep strong light-matter coupling” regime.^79^

Electrotunable plasmonic metamaterials can be further realized via the electrochemically controlled self-assembly of plasmonic nanoparticles at liquid/liquid or liquid/solid interfaces.^99−103^ A reversible electrotunable liquid mirror was demonstrated based on voltage-controlled self-assembly/disassembly of negative-charge-functionalized gold nanoparticles at the interface between two immiscible electrolyte solutions.^100^ The optical properties of the liquid mirror, such as reflectivity and a spectral position of the absorption band, can be tuned in situ within a low applied voltage of ±0.5 V. The electrochemically controlled self-assembly approach opens up a wide range of possibilities for designing electrotunable optical metamaterials, such as switchable mirrors, filters, and displays.

Based on the specific Watson–Crick base pairing of DNA, plasmonic nanoparticles can be self-assembled into metamaterials with great programmability and unprecedented nanometer-scale precision. In a typical example, one group of plasmonic nanoparticles is first functionalized with thiolated single-stranded DNA as programmable linkers, and then mixed with nanoparticles modified with complementary single-stranded DNA, which leads to self-assembly into complex nanostructures (e.g., dimers, trimers, chains, satellites, lattices) by the hybridization of the DNA strands into a double helix.^104−108^ By exploiting DNA origami (a DNA pattern fabricated by the folding of a long single-stranded DNA using specific single-stranded DNAs^109,110^) as the rigid scaffold, it is possible to organize plasmonic nanoparticles into 2D or 3D metamaterials by hybridizing single-stranded DNA modified nanoparticles with their complementary DNA strands extended from DNA origami at designated binding sites with nanometer-scale precision. This method was used to fabricate chiral plasmonic nanostructures showing strong CD at visible wavelengths.^80^ As schematically shown in Figure 5i, DNA origami with 24-helix bundles, which offer nine helically arranged attachment sites for gold nanoparticles, are used as rigid templates. By mixing them with gold nanoparticles functionalized with complementary DNA strands, gold nanoparticles can self-assemble around the DNA bundles, forming left- and right-handed plasmonic nanohelices, respectively (Figure 5j, insets). Due to the strong near-field coupling between the helically assembled gold nanoparticles, these left- and right-banded plasmonic nanohelices exhibit mirrored CD spectra with a characteristic bisignate peak-dip line shape (Figure 5j). Benefiting from the inherent sequence-defined addressability and high rigidity, DNA origami has been widely applied to precisely build plasmonic metamaterials with well-defined structures and optical responses.^97,111,112^

### 3.3 Large-Scale Fabrication Approaches

#### 3.3.1 Anodic Aluminum Oxide Template-Based Patterning for Hyperbolic Nanorod Metamaterials

Porous anodic aluminum oxide (AAO) is a typical self-organized nanoporous material formed by the anodization of an aluminum film.^113,114^ By controlling the anodization conditions, high-density nanopores with diameters in the range of several to hundreds of nanometers can be readily obtained. It is widely used as a template for the electrodeposition of various plasmonic nanostructures (e.g., nanodots, nanorods, nanotubes) into large-scale arrays with low cost and high resolution.^113,115,116^

**Figure 6:**
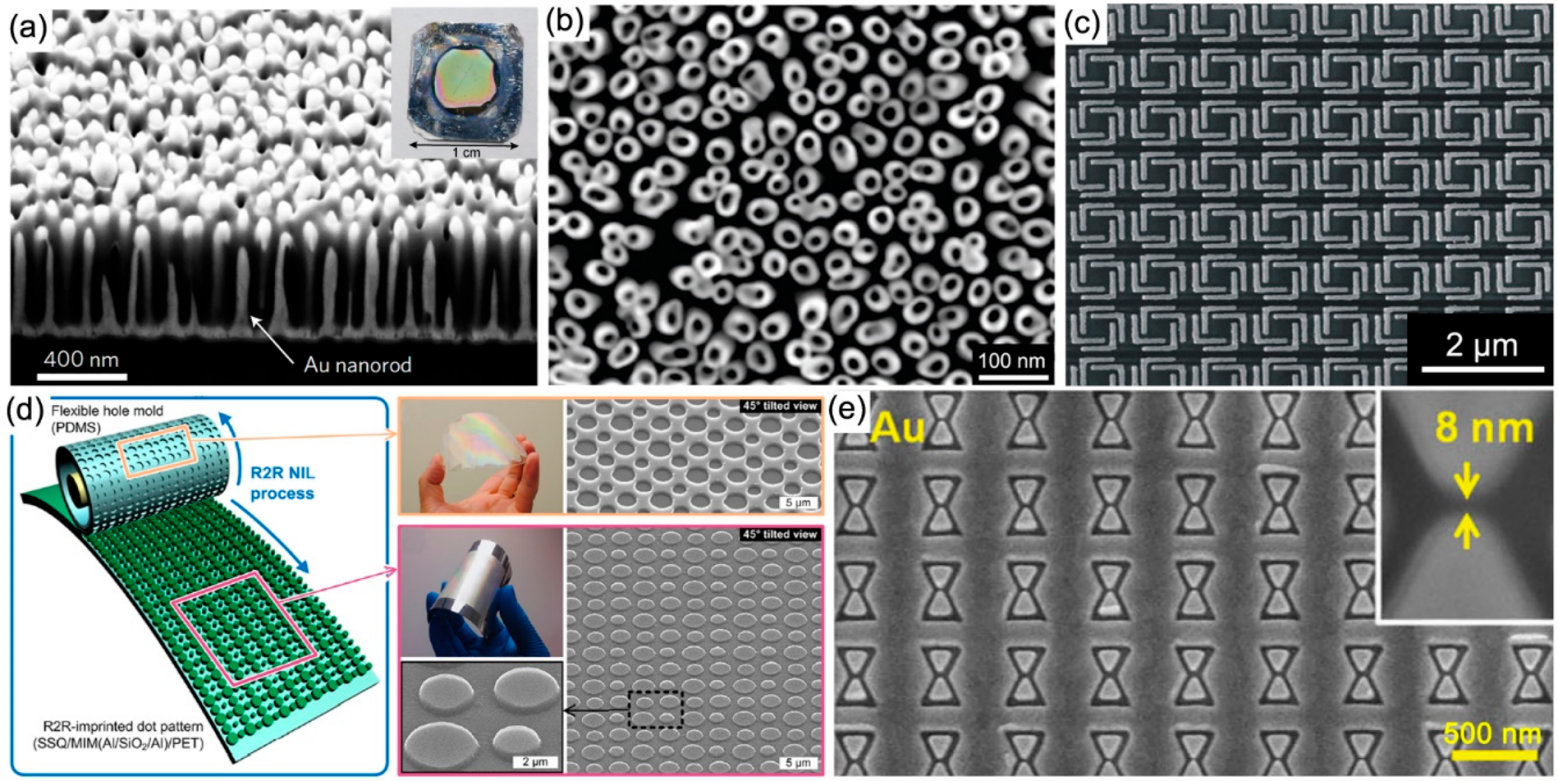
Large-scale fabrication of plasmonic metamaterials. (a) Cross-sectional view of a gold nanorod metamaterial. Inset, photograph of the nanorod metamaterial. Reproduced with permission from ref (119). Copyright 2018 Springer Nature. (b) SEM image of a gold nanotube array. Reproduced with permission from ref (125). Copyright 2010 American Chemical Society. (c) SEM image of a small area of a gold L-shaped resonator metamaterial. Reproduced with permission from ref (130). Copyright 2007 AIP Publishing. (d) Schematic illustration of roll-to-roll imprinting onto a UV-curable epoxy-silsesquioxane-coated Al/SiO_2_/Al/PET substrate under a conformal contact and fabricated nanostructures. Reproduced with permission from ref (131). Copyright 2012 AIP Publishing. (e) SEM top view image of the gold bow tie array. Reproduced with permission from ref (132). Copyright 2013 WILEY-VCH Verlag GmbH & Co. KGaA, Weinheim.

Plasmonic nanorod metamaterials, an archetypal type of hyperbolic metamaterials (HMM), which consist of a periodic array of metallic nanorods oriented perpendicular to a substrate, are fabricated by electrodeposition of metal into porous AAO templates on a substrate.^117,118^Figure 6a shows a cross-sectional view of a gold nanorod metamaterial.^119^ It can be clearly seen that gold nanorods with an average diameter and length of ∼50 and 420 nm, respectively, are closely embedded in an alumina matrix. The diameter and separation of the nanorods (typically in the ranges of 15–65 and 50–120 nm, respectively) are determined by the parameters of the AAO template (i.e., nanopore diameters and separations, regulated by the conditions of the anodization), while the length of the nanorods (typically in the range of 150–1200 nm) is controlled by the electrodeposition time and the thickness of the AAO template (as an upper bound). Benefiting from the scalable electrochemical fabrication technique, such metamaterials can cover macroscopic (centimeters squared) areas with typical nanorod areal densities as high as 10^10^–10^11^ cm^–2^. The functionality of nanorod metamaterials can be further extended by inserting functional materials into the nanorods to form split-rod metamaterials^120,121^ or by coating the surface of the nanorods with functional materials to form core–shell nanorod metamaterials.^122−124^ The former are usually fabricated via sequential electrodeposition of metal (bottom section), functional material (middle section, e.g., ZnO^121^), and metal (top section) into porous AAO templates on a substrate, and the latter can be readily fabricated by first widening the AAO pores to create a shell around the nanorods with nanometer-scale thickness, followed by the electrodeposition of functional materials such as palladium,^123^ polypyrrole,^122^ or nickel^124^ into the shells, to coat each nanorod. Furthermore, by electrodepositing metal around sacrificial polymer nanorods in a porous AAO template, plasmonic nanotube metamaterials can be fabricated (Figure 6b).^125,126^ Coaxial rod-in-a-tube arrays with gap as small as 5 nm can also be realized by sequential deposition of gold nanorods, sacrificial polypyrrole nanoshells, and gold nanoshells into a porous AAO template.^127^

Usually, the geometry and layout of as-fabricated nanorod metamaterials are determined by the fixed AAO template providing quasi-hexagonal arrangement of the nanorods. However, it is possible to precisely engineer the size parameters and arrangement of nanorods in the assembly by using EBL, FIB or NIL to define the templates for electrodeposition. In this way, high-uniformity nanorod metamaterials can be fabricated by the combination of these methods.^128,129^

#### 3.3.2 Nanoimprint Lithography

Nanoimprint lithography (NIL)^133^ is a low-cost and high-throughput technique for nanofabrication. Different from most lithographic approaches that transfer nanostructure patterns via a photo- or electron-induced changes in the resist, NIL transfers patterns mechanically with a stamp, providing a parallel processing for fabrication of plasmonic metamaterials with high throughput and low cost. In this technique, a stamp with a desired pattern is mechanically pressed into the imprint material (usually a polymer or monomer formulation) already coated on a substrate. The mechanical deformation causes the pattern to be transferred into the imprint medium. After a hardening process, the stamp is removed, leaving the nanostructure pattern on the substrate. A plasmonic metamaterial can then be fabricated by standard metal and dielectric depositions and a lift-off procedure. Therefore, with an optimized nanoimprint process, the resolution of NIL is only limited by resolution of the mold pattern, which is fabricated typically by EBL and can be as high as several nanometers.

Based on this approach, various plasmonic metamaterials have been demonstrated.^130,134−136^Figure 6c shows an SEM image of a small area of a metamaterial consisting of an ordered array of four L-shaped resonators.^130^ The total size can be as large as 1 mm × 100 μm, with a critical dimension smaller than 10 nm. By using the roll-to-roll NIL technique, continuous fabrication of large-scale plasmonic metamaterial films can be further realized.^131,137^Figure 6d illustrates the roll-to-roll imprinting of disk patterns on a metal–insulator–metal stack-coated polymer substrate using a flexible hole mold. The mold can continuously imprint the disk patterns onto a linearly fed substrate when it is rolled over the substrate under a conformal contact.^131^

Nanoimprinting can also be carried out directly on metal (e.g., gold, silver) substrates without using any resists by using hard molds to fabricate plasmonic metamaterials.^132,138,139^ Si molds have been proposed to pattern metal films at a high temperature (400 °C) and pressure (300 MPa), with the subsequent mold removal by wet-etching.^139^ This method was also demonstrated at low pressures (<4 MPa) and temperatures (25–150 °C) for imprinting silver and gold plasmonic nanostructures with Si molds (Figure 6e), which could be recycled many times.^132^

NIL provides an attractive approach for fabricating two-dimensional plasmonic metamaterials with high resolution, excellent repeatability, low cost, and high throughput. However, several critical issues need to be solved before it can be used in the highly demanding industry applications. For example, defects can be easily generated after each contact process, and pattern distortions can happen during the demolding process (separation of the mold from the substrate). Also, there is usually a residual layer left on the substrate after NIL, which needs to be removed before subsequent processing.

### 3.4 Incorporation of Molecules in Plasmonic Metamaterials

In order to combine plasmonic metamaterials with the molecular species, techniques for the incorporation of molecules into plasmonic metamaterials were developed. The simplest way is to place plasmonic metamaterials into liquid or gaseous environments where the targeted molecules are dispersed. Upon diffusion, the molecules enter the metamaterials becoming accessible to the near-field of the meta-atoms. This approach is widely used in biochemical and gas sensing applications. Target molecules can also be doped into a solid matrix (e.g., polymers) first and then coated onto the surface of meta-atoms to realize hybrid molecular metamaterial systems. In this case, the distance between the molecules and meta-atoms is fixed and random (with the upper limit of the distance determined by the thickness of the coating).

For more controllable functionalization, molecules can be fixed onto the meta-atoms using the well-established metal surface functionalization techniques, such as thiol-metal (or amino-metal) systems, based on the high affinity of sulfur (such as in a thiol group) to metal (e.g., gold, silver) surfaces. Target molecules can be a part of the thiolated derivatives or bind onto the thiolated groups separately. With this approach, the position and distance of the molecules to the meta-atoms can be precisely adjusted by controlling the length (usually less than 5 nm) of the thiolated group, which is required for engineering the optical interactions between meta-atoms and molecules. Silica shells or polyelectrolyte multilayers fabricated using layer-by-layer assembly are also widely used for the immobilization of molecules with larger molecule–meta-atom distance. Furthermore, target molecules can be bound onto a particular part of the meta-atom by using lithography approaches such as EBL to predefine the exposure area.^140−142^ They can also be used for the specific binding of other functional materials such as quantum dots to selected regions of the meta-atoms.^140,141,143^

## 4 Light-Molecule Interaction in Plasmonic Metamaterials

The interaction of molecules or atoms with plasmonic nanostructures gives rise to a wide variety of phenomena from the modification of the spontaneous emission to plasmon-exciton lasing. In this section, starting with the fundamentals of the light-matter interactions in weak and strong coupling regimes, we overview the intriguing effects of the plasmonic metamaterial environment on the optical properties of molecules.

### 4.1 Weak and Strong Coupling Regimes

**Figure 7:**
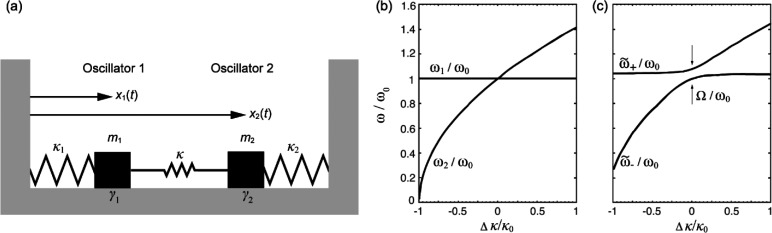
Weakly and strongly coupled oscillators. (a) Model of two coupled lossy oscillators. (b and c) Dependence of the frequency characteristics of weakly coupled (b) and strongly coupled (κ = 0.08κ_0_) (c) oscillators on their spectral detuning. Reproduced with permission from ref (144). Copyright 2010 AIP Publishing.

When quantum emitters are coupled with other resonant systems, depending on the resonant properties of each component and their coupling strength, their interaction can be significantly modified. The physics of the related phenomena can be explained in a very illustrative way considering a model of two coupled oscillators, where one oscillator represents a molecular excitation, the other represents a photonic resonance supported by the metamaterial, and the coupling between them accounts for the light-matter interaction (Figure 7a):^144,145^
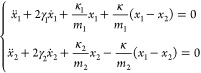
7where *m*_1,2_, γ_1,2_, and κ_1,2_ are the masses, individual (before coupling) loss rates, and spring stiffnesses of the oscillators, respectively, *x*_1,2_ are their positions, and κ is their coupling coefficient. We assume that the oscillators are well-defined, so when they are not coupled, their loss rates are much smaller than their frequencies (γ_1,2_ ≪ ω_1,2_). We also consider the case when their resonant frequencies (particularly their real parts) are the same ω_1,2_ = ω_0_. The latter assumption corresponds to the situation when the molecular transition and the metamaterial mode are in resonance. For illustrative purposes, we take the masses of the oscillators to be identical *m*_1_ = *m*_2_ = *m*, so their coupling is characterized by a single coupling coefficient . The solutions of eq 7 can be found in the form of damped oscillations:
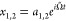
8where Ω̃ = ω̃ + *i*γ̃ is the complex-valued oscillation frequency. Considering the coupling as a small perturbation, so that solutions will correspond to the slightly modified eigenstates of each oscillator Ω̃_1,2_ = ω_1,2_ + Δω̃_1,2_ + *i*(γ_1,2_ + Δγ̃_1,2_) and applying a perturbative approach with a small parameter Ω/ω_0_, one can find a frequency shift of the oscillators due to coupling between them
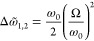
9and the change in their damping rates
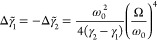
10

To further develop the analogy with a molecular system, one of the oscillators can be assumed to have a very low loss γ_1_ ≪ γ_2_, corresponding to a narrow frequency response representing the molecular transition. Then, for the case of reasonably weak coupling Ω^2^ ≪ γ_2_ω_0_, the frequency shifts will be very small for both oscillators. In particular, they will be much smaller than the line width of the second lossy oscillator Δω̃_1,2_ ≪ γ_2_, so the overall spectral response of the system remains unchanged and the oscillators keep their general behavior. For the molecular-metamaterial system, this means that the presence of the metamaterial practically does not change the transition frequency of the molecule and the presence of the molecules does not change the behavior of the metamaterial mode. This is the so-called weak coupling regime.

Even in this case, however, the coupling can have a very strong impact in terms of the loss. Particularly, if the coupling has a sufficient strength , the lifetime of the low-loss “molecular” oscillator will be significantly modified so its change is of the same order as its initial value |Δγ̃_1,2_ | ≳ γ_1_. By an analogy, the rate of the spontaneous decay, described by the oscillator, can be substantially increased in the presence of the metamaterial through the coupling to the metamaterial modes. It is worth stressing that both the above conditions can be simultaneously satisfied, as they require only γ_1_ ≪ γ_2_. It is also interesting to note that the relative line width modification of the second “metamaterial mode” oscillator will not be essential, as the initial loss is comparably quite high. This modification of the emission rate in a weak coupling regime can be rigorously described considering the local density of optical modes, as will be done in subsections 4.2 and 4.3.

In the strong coupling regime, when the coupling rate is comparable to or larger than the damping rate of both oscillators (the coupling time is comparable to or shorter than the oscillator lifetimes), the situation is very different. Physically this corresponds to the case when the oscillators can significantly exchange energy during their lifetime (even multiple times). Solving the model (eq 7) for this case and taking the lossless case to expose a clear physical meaning, one can determine the frequencies of the new eigenmodes of the system^144^
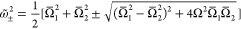
11where  and . The eigenmodes in this case are the collective oscillations representing hybrid states involving both resonant systems. In the molecule-metamaterial system, such eigenmodes, visible in its extinction or absorption spectra, are mixed light-matter states with specific anti-crossing dispersion. If we keep the parameters of one of the oscillators constant (κ_1_ = κ_0_, *m*_1_ = *m*_0_, resonant frequency ω_1_ = ω_0_, horizontal line in Figure 7b) and change the spring constant of the other (for simplicity taken with the same mass), so its uncoupled eigenfrequency sweeps to cross the one of the first oscillator through the variation in κ_2_ = κ_0_ + Δκ (the curved line in Figure 7b), the dispersions of the coupled states experience an anti-crossing with a frequency split Ω proportional to the coupling coefficient κ (Figure 7c). The phenomenon of the anti-crossing is also called the Rabi splitting. If the loss is introduced, the mode dynamics of the system become complex, but the main characteristics of the anti-crossing in a strong coupling regime remain. The splitting in this case might be indiscernible due to comparable or larger resonance widths. Here, a general condition for its observation is given by the expression:
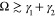
12

One needs to stress here that although the phenomena of weak and strong coupling were illustrated above for the case of localized resonances, they also exist for interaction of molecular resonances with propagating modes as in the case of, e.g., enhanced spontaneous emission in the presence of waveguided metamaterial modes^67^ (see subsection 4.3 below) or strong coupling of emitters to propagating SPPs.^146^

### 4.2 Weak Coupling: Modification of the Spontaneous Emission Rate

Since the pioneering work of Purcell,^147^ it has been known that the spontaneous emission rate of an atom, a molecule, or any other emitter is not a universal characteristic, defined only by the emitter internal properties, but is heavily influenced by the electromagnetic environment. This happens through an increase of a local density of optical states (LDOS) available for the emission. The modification of spontaneous emission by media, interfaces, or specially designed physical systems is called the Purcell effect, and the ratio of the emission rate to its free-space counterpart is called the Purcell factor.

Even a simple metallic surface creates a rise in LDOS in comparison to the free space, as it supports SPPs and lossy surface waves.^148^ This phenomenon is actually universal for any plasmonic system and can be related to the characteristic field enhancement provided by the plasmonic objects. Furthermore, by nanostructuring the metal into metamaterials, metasurfaces, and individual nanoparticles, it is possible to design the supported modes and their density, which can be used to engineer the light-matter interaction, particularly the electromagnetic behavior of molecules in the vicinity of the structures. Electronic transitions in molecules can be summarized in the framework of a Jablonski diagram showing the possible radiative and nonradiative transitions. Absorption, fluorescence, and phosphorescence can be efficiently influenced in a plasmonic environment, including both allowed and forbidden transitions, and the selection rules can be relaxed due to the symmetry of the plasmonic field. In this subsection we will discuss this phenomenon in the context of the modification of the spontaneous emission of excited molecules in plasmonic metamaterials or near plasmonic metamaterials and metasurfaces.

In the regime of weak coupling of the emitter with the environment, the light-matter interaction presents a small perturbation in a Hamiltonian describing the system of the emitter and the electromagnetic field. Applying a standard quantum-mechanical procedure, the rate of spontaneous emission of an excited molecule, defined by interaction with zero oscillations of the electromagnetic field, can be calculated using Fermi’s golden rule:^149^
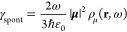
13where **μ** = ⟨*g*|**μ̂**|*e*⟩ is the matrix element of the dipolar transition operator **μ̂** involving the excited |*e*⟩ and ground |*g*⟩ states and *ρ*_*μ*_(**r**, ω) is the position- and frequency-dependent partial LDOS for the transition dipole moment with the considered orientation. The latter can be related to the total LDOS via a proper averaging over dipolar transition directions if there is no fixed one. Thus, as was mentioned above, plasmonic metamaterials and metasurfaces having highly resonant and/or unusual optical responses offer the opportunity for engineering the LDOS and, consequently, the spontaneous emission of the molecules or atoms. On one hand, the provided enlarged LDOS will lead to an increased spontaneous decay rate and, therefore, the possibility to realize faster light sources for, e.g., optical communication. On the other hand, the rise of the LDOS related to the plasmonic modes coupled to the free-space radiation can lead to the increase of the local quantum yield η of the fluorescent molecules and, therefore, a higher brightness of the emission:
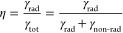
14where γ_rad_ is the emission rate into photons, which can be either free-space radiation or photons coupled to the waveguided modes, and γ_tot_ is the total decay rate which also includes the energy transfer into nonradiative channels given by γ_non-rad_. Therefore, the increase of the brightness is a more intricate question, because the presence of the metallic nanostructures will also result in the introduction of additional loss channels, related to nonradiative plasmonic modes, surface lossy waves, and damping of the energy into electron–hole excitations,^148^ thus also increasing γ_non-rad_.

From the point of view of the LDOS enhancement, hyperbolic plasmonic metamaterials present a particular interest. Generally, the LDOS is the number of optical states in the infinitesimal frequency interval from ω to ω + dω, normalized by dω. In other words, it includes optical states located between two constant-frequency surfaces near ω in the *k*-space. In the case of natural materials with an elliptical dispersion, such region is bounded by two infinitely close elliptical surfaces and has a finite (and infinitely small) volume (Figure 2). In the case of hyperbolic metamaterials, however, this space resides between two infinitely close hyperboloids, which extend infinitely in the high-*k* directions, resulting in an infinitely high LDOS. Furthermore, in contrast to plasmonic resonances which provide LDOS enhancement only in the vicinity of the resonance frequency, hyperbolic metamaterials provide a broad-band Purcell effect at all frequencies where the dispersion is hyperbolic.

**Figure 8:**
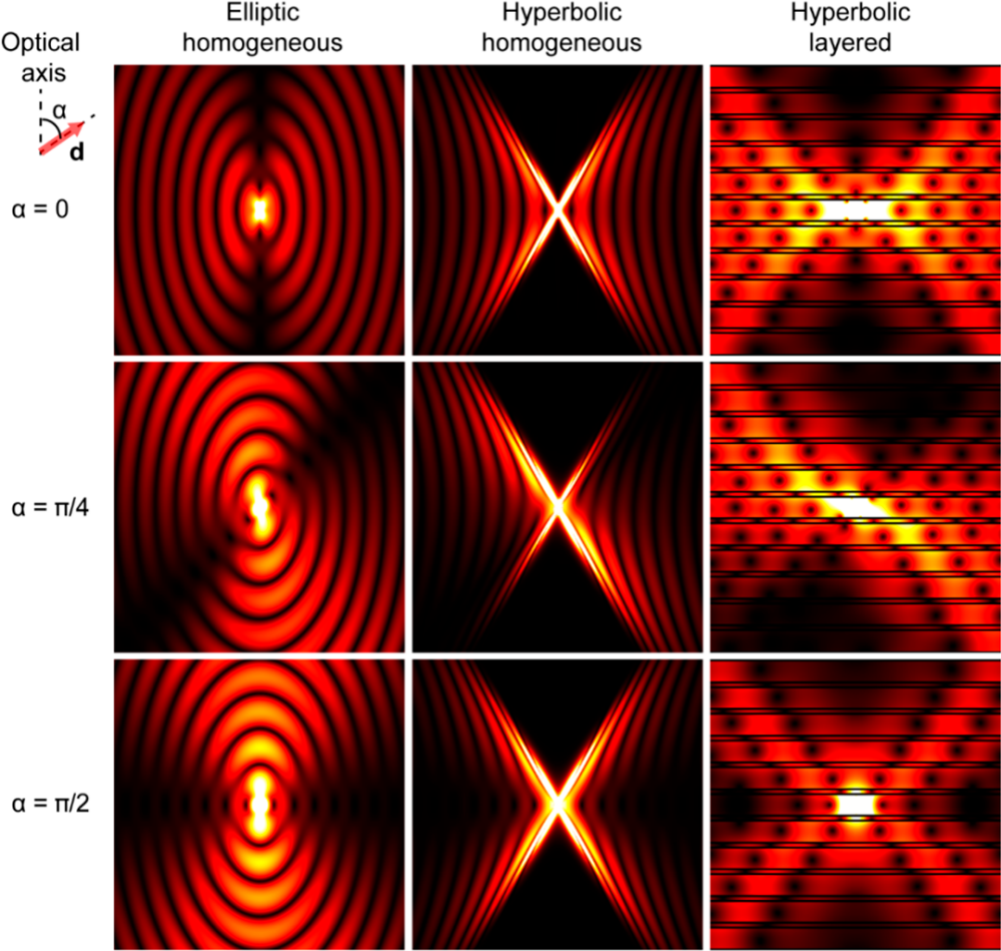
Emission patterns (|Re(**E**)|) of a radiating dipole placed inside an anisotropic material with an elliptical dispersion (left column, ε_∥_ = 3, ε_⊥_ = 1), a hyperbolic metamaterial described by an EMT (middle column, ε_eff,∥_ = 3 + 0.2*i*, ε_eff,⊥_ = −1 + 0.2*i*), and a multilayer hyperbolic metamaterial realization (silica (22 nm)/gold (5 nm)) corresponding to the considered EMT case. The maps are presented for three dipole orientations with respect to the optical axis, given by angle α. Reproduced with permission from ref (47). Copyright 2017 American Physical Society.

Additionally, the emission inside or near the hyperbolic metamaterial can possess high directionality. Indeed, the high LDOS is achieved for the modes inside a narrow wave vector cone marked by the hyperboloid asymptotes, and these are the modes into which the radiation will be predominantly emitted. The related wave vectors mark the directions of the phase velocities of the emitted modes. The actual directions of the emission, in which the energy is emitted, are defined by the directions of the group velocities of the modes (or the Poynting vector), which are perpendicular to the isofrequency surfaces. They also produce a cone with an angle of π/2 – θ in respect to the metamaterial optical axis *z*, where θ is the corresponding angle of the hyperboloid asymptotes. In Figure 8, the directions of the phase velocity are shown by the wave fringes, and the energy propagation directions, by the emission intensity (a cross section of the cone). A substantial modification of the emission directionality is observed in the hyperbolic regime in comparison with the case of the material with exaggerated anisotropy in the elliptical regime (cf. Figure 8 left and middle columns).

These considerations are related to the theoretically infinite LDOS and, therefore, a singular Purcell factor determined by an infinite extent of the hyperboloids in the *k*-space for (1) the lossless case and (2) EMT theory assuming an infinitely fine level of nanostructuring. In real metamaterials with a certain nanofabrication pitch and unavoidable losses, however, the hyperboloids are transformed into confined hyperboloid-like surfaces (see subsection 2.2 for the details). Additionally, the emission rate will be clamped due to a spatial dispersion of the metamaterial and a finite size of the emitter. Nevertheless, the fluorescence enhancement with the use of hyperbolic metamaterials presents a very elegant physical approach, which was extensively theoretically studied and experimentally demonstrated.^53,67,82,150−161^ Devices made on this basis can find applications in fast optical communication, biosensing, fluorescence imaging, single molecule detection, broad-band single photon sources, and quantum optics.

### 4.3 Spontaneous Emission Enhancement in Hyperbolic Metamaterials

Spontaneous decay of various emitters in the presence of hyperbolic metamaterials was investigated using time-resolved photoluminescence spectroscopy.^162^ The considered emitters included quantum dots, quantum wells, 2D materials, nitrogen-vacancy centers, and molecules; the latter presents a particular interest in the context of this review.^150,152,155,156^ The spontaneous decay rate of dye molecules in an epoxy layer at the interface with multilayer hyperbolic metamaterials was measured to be 1.7 times higher than its counterpart in the pure epoxy environment.^150^ Comparing with a control sample of a flat metal film (assumed to have the same near-field quenching loss into the metal), the increase of the spontaneous decay rates was attributed to the coupling to the high-*k* modes of the hyperbolic metamaterial. Further studies showed that the decay of dye molecules located inside the multilayer metamaterial, particularly in the dielectric layers producing it, is faster, showing a Purcell factor enhancement in the range of 3–6 for various geometrical parameters.^152^ The increase of emission into radiative modes (including photons and metamaterial modes) was estimated to be even higher (30–50 times); the difference occurs because the radiative rates enter the overall Purcell enhancement together with nonradiative losses, which are quite substantial, as the nominal quantum efficiency of the used IR140 dye in the PMMA matrix is quite low (9%). A careful study of spontaneous emission at various wavelengths with the use of quantum dots showed an increase of spontaneous rates at the spectral point where the metamaterial enters the hyperbolic regime.^156^ It was also shown that the spontaneous emission decay of the emitters located on the top of the metamaterial can be qualitatively, but not quantitatively, characterized by describing the optical properties of the metamaterial with the EMT (see below about the validity of EMTs for the emitters inside a nanowire metamaterial).

**Figure 9:**
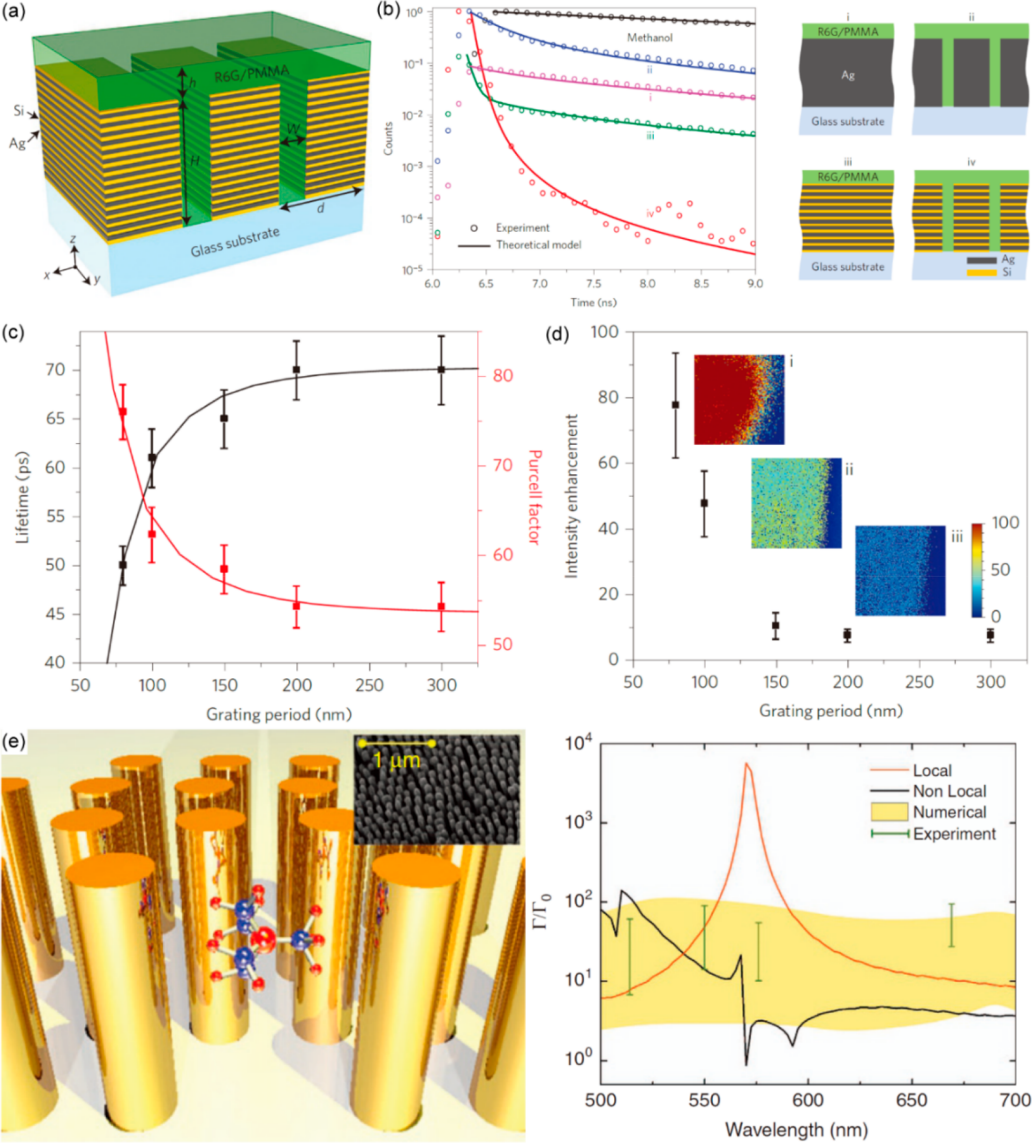
Fluorescence enhancement with hyperbolic metamaterials. (a) Schematics of the multilayered HMM. (b) Time-resolved fluorescence signals measured in various environments shown on the right. (c) The dependence of the lifetime and the related Purcell factor for the nanopatterned hyperbolic metamaterial (case (iv) in panel b) normalized by its counterpart for the uniform hyperbolic metamaterial (case (iii) in panel b) on the array period. (d) Enhancement of the fluorescence intensity as a function of the nanopatterned hyperbolic metamaterial period with the same normalization as in panel c. Insets show optical images of the hyperbolic metamaterial samples with (i) 80 nm, (ii) 100 nm, and (iii) 200 nm periods. Reproduced with permission from ref (82) Copyright 2014 Springer Nature. (e) Schematics of the nanorod HMM and the associated Purcell enhancement for the emmiters inside the metamaterial. The yellow shaded area indicates the range of the Purcell factor values for various positions of the orientationally-averaged emiter with respect to the nanorods. Reproduced with permission from ref (53). Copyright 2017 Springer Nature.

As we saw from the experimental results presented above, in metamaterials and, particularly, in hyperbolic metamaterials, the spontaneous decay rate can increase, potentially leading to faster light sources, but the radiation in this case is preferentially emitted into the high-*k* waveguided modes (see subsection 2.2). These modes are trapped inside the metamaterial due to the wave vector mismatch to the free-space photonic modes leading to their total internal reflection at the metamaterial interfaces. This problem can be solved by the implementation of nanostructuring of the metamaterial, which, supplying an additional wave vector, will couple the metamaterial modes to the free-space radiation.^82,163,164^ Particularly, it was experimentally shown that a grating-patterned multilayer hyperbolic metamaterial (Figure 9a) can offer a 76-fold enhancement of the Purcell factor (Figure 9b and c) together with an 80-fold enhancement of the fluorescence intensity radiated in the far-field compared to the uniform HMM (Figure 9d). The intensity enhancement originates from both grating-assisted outcoupling of the HMM modes and better pumping due to the larger local pump intensity. Importantly, the Purcell factor and the fluorescence intensity increase with the decrease of the grating period, confirming the coupling of the dye emission to the higher-*k* metamaterial modes, which are converted to free-space radiation with the shorter-period gratings supplying larger momenta. This finding is logical as higher-*k* metamaterial modes have a larger LDOS, which follows from the topology of the hyperbolic dispersion (Figure 2b and c). It was theoretically shown that the Purcell factor enhancement can be tuned by varying the thicknesses of the layers producing the hyperbolic metamaterial, as this changes the metamaterial optical properties described by the EMT via the variation of the metal filling factor *f* (see eq 5). The enhancement of the fluorescence intensity through nanostructuring of the multilayer hyperbolic metamaterial into a resonant antenna was also theoretically investigated.^158,165,166^ This provides the possibility to manipulate spontaneous emission in metamaterial components with subwavelength volumes, in contrast to the approach based on traditional optical cavities.

The increase of the spontaneous decay rate, underlined by the same physical mechanism, was observed in nanorod hyperbolic metamaterials both at a metamaterial interface^151^ and inside a metamaterial layer.^53,67^ In the latter case, using dyes with various emission wavelengths, it was shown that spontaneous emission of the dye can be coupled to the waveguided modes of the metamaterial slab.^67^ Furthermore, this can be used for spectral shaping of the emission. A particular interest in the case of nanorod hyperbolic metamaterials received a question of the influence of the nanostructuring-related nonlocality on the rate of the spontaneous decay (Figure 9e).^53^ In fact, in this respect the nonlocality was shown to play a crucial role, fundamentally limiting the Purcell effect through the correction to the metamaterial LDOS due to the so-called additional electromagnetic modes, related to collective excitation of cylindrical surface plasmons supported by the nanorods. The local EMT predicts that the Purcell factor enhancement has an enormous peak at the ENZ spectral point, where the metamaterial dispersion is transformed from elliptical to hyperbolic. Nonlocal EMT results in a flatter wavelength dependence of the spontaneous decay rate, which was confirmed by experimental observations and numerical modeling. The results show the essential breakdown of the local EMT for the description of quantum emitters located in the bulk of a nanorod metamaterial and the crucial importance of taking into account nonlocality if one stays within the effective medium description. On the other hand, the numerical simulations of the emission in the exact nanorod array environment demonstrated a dramatic dependence of the Purcell factor on the position and transition dipole orientation of the emitter.

It was shown that for forbidden non-dipolar singlet–triplet transitions, the increase of the LDOS was not enough to explain an experimentally observed 2750-fold increase of the spontaneous decay rate, and strongly inhomogeneous electromagnetic fields inside the metamaterial need to be considered to further facilitate the decay process.^161^ An interesting phenomenon related to the spontaneous decay modification by the electromagnetic environment is the modified Förster energy transfer (in a classic description, when an excited donor molecule transfers the energy to a receiving acceptor molecule via the near-field interaction), which was studied in the case of metamaterials.^167,168^ Another effect happening in the weak coupling regime is electromagnetically induced transparency,^145^ which was observed for interaction of molecular vibrations with an SRR metamaterial.^169^

The modification of the LDOS in hyperbolic plasmonic metamaterials affects not only the rate of the emission but also its directivity, which makes a pronounced impact on the molecular optomechanics inside the metamaterial. Using an analytical radiation reaction approach based on the Langevin local quantization of electromagnetic excitations, a universal theory of self-induced optical forces acting on a molecule inside an anisotropic homogeneous medium with arbitrary absorption and dispersion was derived.^47^ Particularly, it has been shown that a radiating molecule experiences a giant self-torque inside a multilayer hyperbolic metamaterial described by an EMT, two orders of magnitude larger than in materials with the highest anisotropy available in nature. The emitting molecule in this case was represented by a finite-sized dipole with a Gaussian distribution of a dipole density and realistic spatial dimensions (2 nm) corresponding to, e.g., rhodamine-like molecules. The origin of the self-torque is the maximization of the emitter radiation efficiency and the corresponding minimization of the potential energy of the dipole in the near-field, resulting in a preferable alignment of the dipole along the metamaterial optical axis. The effect persists beyond the EMT description, although with a smaller and broader spectral peak of the torque, in a multilayer metamaterial realization.

### 4.4 Strong Coupling: Hybridized Light-Matter States

Strong coupling is an intriguing phenomenon related to an oscillation of the energy between the excitations in matter and electromagnetic modes happening within their lifetimes. The strength of the coupling determines both the absorption and emission properties of molecules inside the metamaterials, effectively governing their optical behavior. A careful description, possible at various levels from classical to fully quantum, shows that this results in the hybridization of the material and photonic states, when the eigenmodes of the system are given by the mixed light-matter states.^145,146^

**Figure 10:**
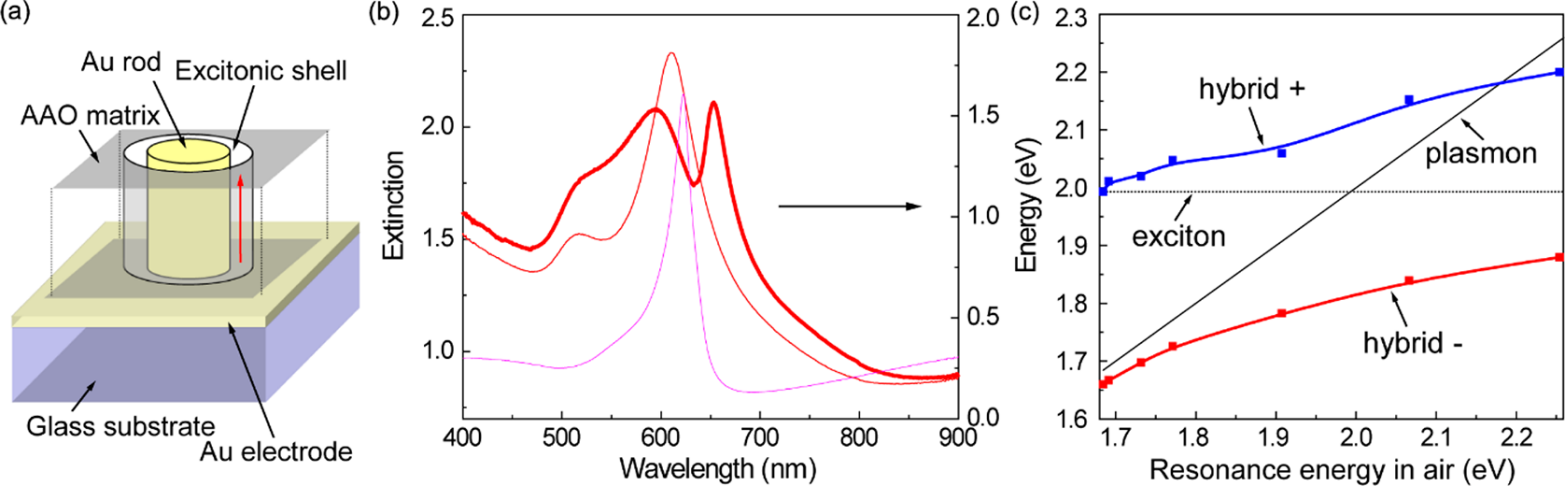
Strong coupling of J-aggregate excitons with plasmonic modes in a nanorod metamaterial. (a) Schematics of the metamaterial unit cell. (b) Extinction spectrum of the J-aggregate-functionalized nanorod metamaterial. (c) Anti-crossing of the hybrid plasmonic/excitonic modes, obtained by varying the metamaterial mode spectral position by changing the shell thickness around the nanorods. Reproduced with permission from ref (185). Copyright 2007 American Chemical Society.

Using the fact that the properties of metamaterials are defined by their geometrical parameters, the strength of the coupling can be engineered, as this gives a means to control a spatial overlap between the metamaterial modes and molecules, as well as a spectral overlap with molecular emission profile. A prominent example of the engineered strong coupling in a metamaterial-molecular system was demonstrated in a study of an interaction of molecular excitons and plasmonic modes in a core–shell metamaterial (Figure 10a). J-aggregate molecules were deposited into an empty shell around the nanorods produced via partial etching of the Al_2_O_3_ matrix. Changing the shell thickness controls the spectral position of the metamaterial mode, allowing design of the spectral overlap between extinction peaks of the metamaterial and of the excitons leading to the demonstration of an anti-crossing between them (Figure 10b and c). The strong coupling in this system can be phenomenologically described by the interaction of the molecular excitons with a metamaterial resonance. Strong coupling of rhodamine 6G dye with a plasmonic guided mode on the surface of a multilayered hyperbolic metamaterial was also observed through the splitting of reflection dips.^170^ The coupling strength in this system can be tuned through engineering the optical properties of the metamaterial via the metal filling factor. In the terahertz spectral range, strong coupling was demonstrated between the metasurface plasmons and molecular vibrations.^171,172^ Particularly, it was shown that if the metasurface is chiral, weak and strong coupling regimes can be interchanged via the change of the handedness of the illuminating light, which is associated with different characteristics of the excited plasmonic modes.^171^

Generally, in the photonic systems with modest Purcell factors, the losses and broadening of the emission line lead to the deterioration of the strong coupling effects, while in plasmonic systems they do not practically influence the relaxation process, as the emission happens at much faster time scale (picoseconds vs nanoseconds for the nonradiative broadening).^173^ Overall, in strongly coupled systems, it was shown that the radiative decay may be nonexponential with the energy being transferred from the emitter to the electromagnetic mode and back.^173^ The role of strong coupling under the gain conditions is important for the understanding of the mechanisms of metamaterial loss compensation (see section 5 for the details).^174^ On the basis of strong coupling between dye molecules and the modes on nanostructured plasmonic surfaces, exciton–plasmon lasing has been demonstrated^175^ (for a detailed discussion see subsection 5.1). The spatial coherence of the hybrid exciton–plasmon modes can extend up to a distance of 10 μm even when the presence of the excitonic component in the hybrid mode is 80%.^176^

With the increase of the coupling strength, when the coupling time becomes comparable with the period of the electromagnetic oscillations (and the Rabi splitting becomes comparable with the resonant frequencies), the system enters a so-called ultrastrong coupling regime. This was observed, e.g., for molecular optical transitions and high-Q nanocavity optical modes^177^ or molecular vibrational transitions and gap surface plasmons.^178^ Apart from the progress in fundamental understanding of the light-matter interaction, including the studies of chiral phenomena,^179^ strong coupling is important in many applications, particularly, electronic devices,^180^ nonlinear harmonic generation,^181^ and all-optical light control,^182^ lasing,^175^ and engineering chemical reactions.^183,184^

## 5 Plasmonic Metamaterials with Gain Media

As was discussed above, plasmonic metamaterials and metasurfaces provide a high density of local optical states and through this offer an opportunity to increase spontaneous emission rates. Thus, the incorporation of a gain, based, e.g., on molecular materials, inside or in the vicinity of metamaterials creates a rigorous platform for the realization of bright and fast incoherent light sources and lasers. Furthermore, through the design of meta-atoms and their arrangement and, therefore, the spectrum of the supported metamaterial modes, one can engineer spectral and directional properties of the emitted light. In this section, we overview recent developments in loss compensation, amplification, and lasing in fishnet and nanorod plasmonic metamaterials, also touching the topic of incoherent sources and lasers based on plasmonic crystals.

### 5.1 Loss Compensation, Amplification, and Lasing in Fishnet Plasmonic Metamaterials

Fishnet metamaterials present special interest in metamaterial research due to their ability to provide a negative effective refractive index, which opens a prospect for a plethora of applications. They can be fabricated by the multilayered EBL,^186^ FIB lithography,^187^ and large-scale nanoimprint lithography^188^ approaches introduced in section 3. The appearance of the negative effective refractive index in fishnet metamaterials relies on the simultaneous realization of negative effective permittivity and permeability utilizing an out-of-phase and enhanced response of the structure near its plasmonic resonance. At the same time the loss in the metal leads to damping of the resonant response and, therefore, difficulty for the effective optical parameters to reach negative effective values. Thus, adding a molecular-assisted optical gain in the dielectric component of the metamaterial, usually realized as a dye-doped dielectric, offers a prospective way to solve this problem. Furthermore, an inevitable transition of the metamaterial into a lasing regime with the increase of the pumping^189^ leads to the possibility of realization of coherent light sources with characteristics unattainable with usual lasers.^34^ To achieve the gain levels needed for substantial loss compensation or lasing, intense pumping using pulsed lasers is usually required.

**Figure 11:**
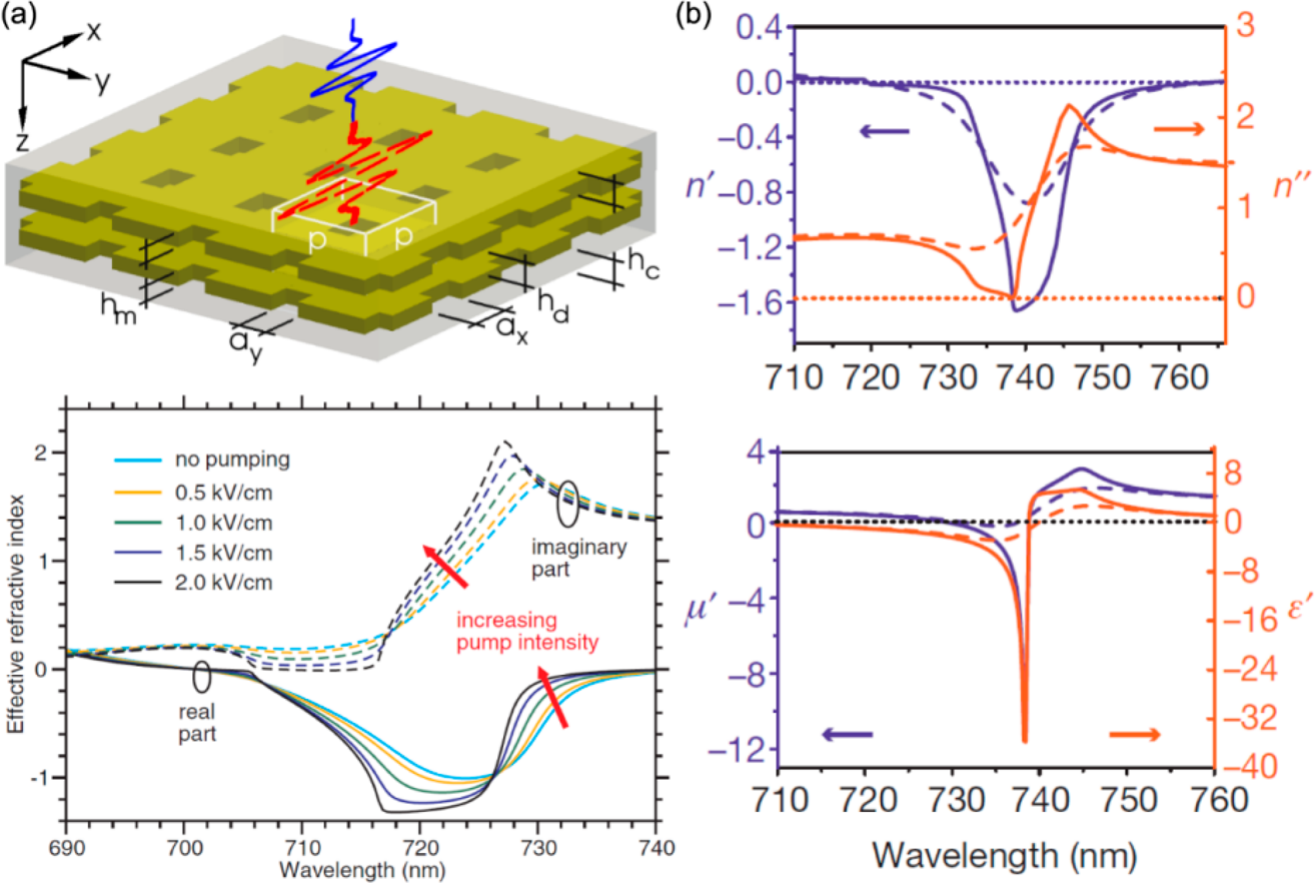
Loss compensation in fishnet metamaterials. (a) Theoretical idea and numerical demonstration of loss compensation in a dye-functionalized fishnet metamaterial. Adapted with permission from ref (191). Copyright 2010 The American Physical Society. (b) Experimental spectra of the refractive index, permittivity, and permeability for low (dashed lines) and high (solid lines) pump intensities for the metamaterial in panel a. Adapted with permission from ref (197). Copyright 2010 Springer Nature.

The possibility of compensating and overcompensating losses in fishnet metamaterials was extensively theoretically investigated (Figure 11a).^190−196^ In this setting, a gain produced by dye molecules can be described using a four-level semiclassical model with two (pumping and gain) dipole transitions.^191^ Particularly, the system was self-consistently numerically simulated using a full-vectorial Maxwell-Bloch approach implemented in a finite difference time domain code. It was shown that at the pump field magnitude of 1.85 kV/cm it is possible to achieve full loss compensation in the negative refractive index regime in a few-nm spectral region around the wavelength of the molecular transition and even amplification of the probe signal (negative absorption) with further increase of the pump. Here, one needs to note that the overcompensation and negative absorption do not necessary mean that the transmitted signal will be higher than the incident one, as a substantial part of the beam might be reflected. It was analytically shown that, in the overcompensation mode, the system enters an instability regime and tends to make a transition to lasing,^189^ maybe at higher pump powers, to compensate the emission output. In an amplification regime, when the system amplifies the incident light but does not yet enter lasing regime (the gain is not sufficient to compensate the output lasing “losses”), transmission values slightly higher than 100% were predicted.^192^

As soon as the dye-functionalized fishnet metamaterial starts to enter the lasing regime, an interesting question arises which mode or modes will be lasing. Within a gain spectral window of the dye, there can be two metamaterial modes satisfying the standing wave Bragg band-edge condition, one of these modes can be bright and the other dark.^195^ Time-resolved numerical simulations showed that both modes can enter the lasing regime and, moreover, revealed a dynamic competition between them. With an increase of the pumping power, it is the bright mode that reaches the lasing threshold first, favored by the chosen spectral profile of the gain. It was demonstrated that by the proper choice of the dye with the spectral overlap with one mode or the other, or both (or conversely by adjusting the geometrical parameters of the fishnet for a given dye), it is possible to engineer individual lasing of each mode or their simultaneous coexistence. Here, to obtain the needed spectral characteristics, the openings in the fishnet were chosen to be of a rectangular form, but with a symmetric square-opening design and the predefined spectral gain window leading to suppression of the dark mode, dynamic competition between two bright modes of orthogonal polarizations can be demonstrated.^194^

Various other designs for gain-assisted molecular metamaterials were considered, e.g. based on split-ring,^199,200^ split-ring/rectangular opening,^198^ and double-split-ring^201^ resonators, 3D SRR configuration producing a toroidal resonant structure,^202^ and negative index materials superlattices.^203^

Although there is a lack of experimental demonstrations of this type of dye-functionalized metamaterial lasing, full loss compensation in the regime of a negative real part of the effective refractive index of a fishnet metamaterial has been shown (Figure 11b). The plasmonic resonance in this case became much sharper, improving the negative index characteristics. The gain-assisted modulation of losses can be used for all-optical switching and will be further discussed in the next section.

### 5.2 Stimulated Emission and Lasing with Hyperbolic Metamaterials

As we discussed above, plasmonic metasurfaces and metamaterials (as well as individual plasmonic nanostructures) offer a high LDOS and, therefore, a local enhancement of the Purcell factor. This increases the spontaneous decay rate and, although increasing the threshold pump powers, enables an accelerated time response of plasmonic lasers and the possibility of their ultrafast modulation.^204^ The high LDOS near plasmonic nanostructures is usually associated with the resonances and, therefore, happens only in a certain spectral range. At the same time, we saw above that hyperbolic metamaterials provide a high LDOS in a broad spectral range in the hyperbolic regime and, therefore, offer a very versatile platform for use with various molecular emitters.

If hyperbolic metamaterials are covered with a dye-doped polymer film, the dye molecules will experience a high LDOS in the region a few tens of nanometers from the metamaterial surface. Due to the simplicity of fabrication, samples of this kind were primarily studied in the experiments. First, the phenomenon of amplified spontaneous emission (ASE) was studied.^205^ A multilayer (type II) 25 nm (silver)/25 nm (MgF_2_) hyperbolic metamaterial was covered with a 200 nm HITC-doped PMMA film, and the entire structure was pumped with 5 ns pulses from an optical parametric oscillator. A spectrum of light emitted from the hybridized metamaterial shows clear narrowing when the pump power increased above a certain threshold, characteristic of ASE. The threshold pump power for reaching the ASE regime was found to be 2.5 times lower and the emission power increase with the pumping power ∼3 times higher than in the case of an unstructured metallic film covered with the dye, used for comparison. These observations, within experimental uncertainties, agreed well with a ∼2 times enhancement of the Purcell factor determined by measuring spontaneous emission kinetics. The increase of the LDOS near the metamaterial interface, compared with that provided by usual SPP waves at the surface of the metallic film, was attributed to the influence of metamaterial bulk plasmon modes produced by coupled gap plasmons supported by the multilayer structure. In either case, outcoupling of these modes to the far field radiation is needed to observe the emission signal, which was facilitated by structure imperfections.

**Figure 12:**
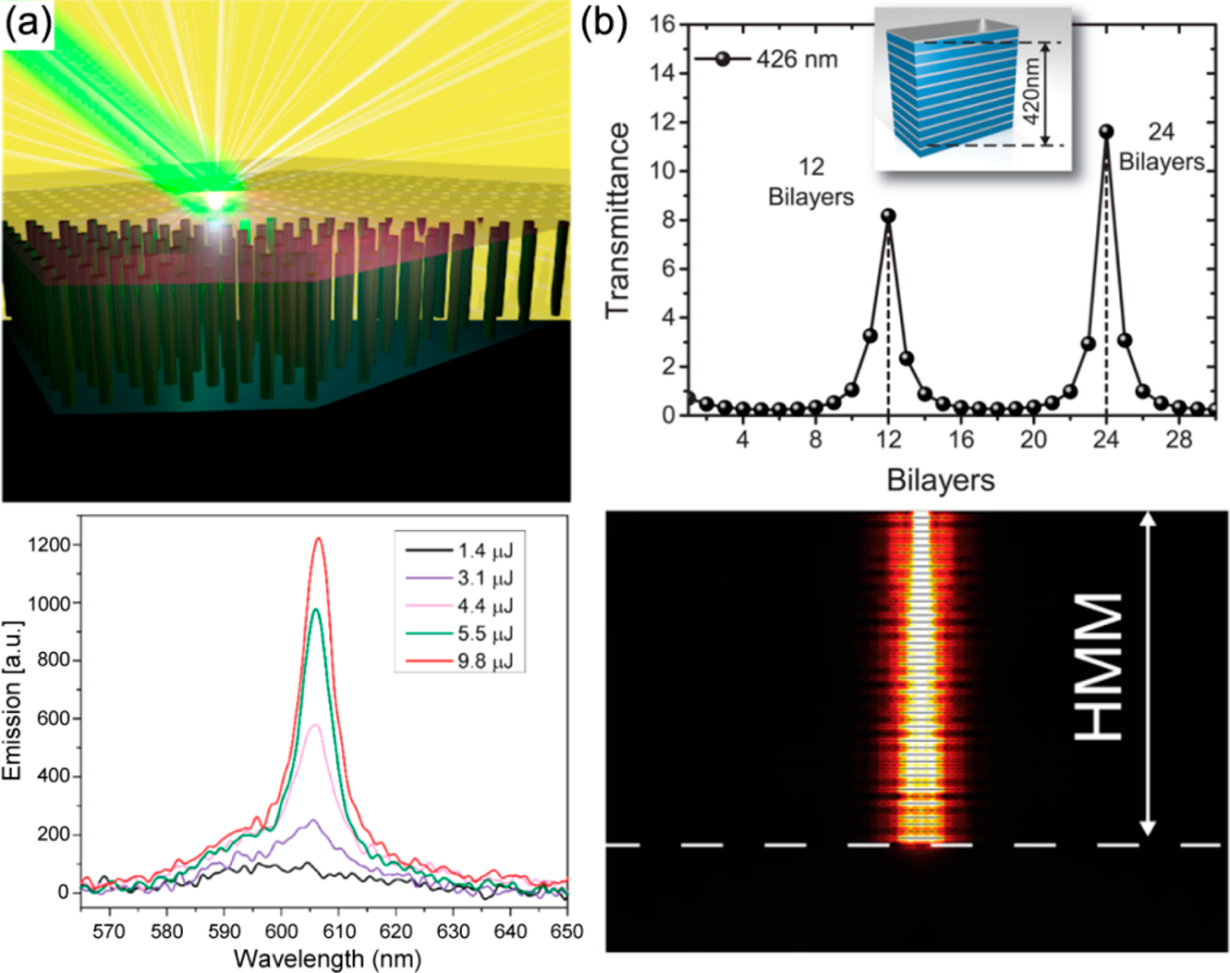
Lasing in hyperbolic metamaterials. (a) Schematics of a dye-functionalized hyperbolic nanorod metamaterial and the emission spectra for different pump intensities. Adapted with permission from ref (206). Copyright 2017 American Chemical Society. (b) Transmission spectrum showing the beam amplification (top) and nondiffracting beam propagation (bottom) in a gain-assisted multilayer hyperbolic metamaterial. Adapted with permission from ref (212). Copyright 2017 American Chemical Society.

Dye-functionalized hyperbolic metamaterial lasing was also studied.^206^ The hyperbolic metamaterial was produced by an array of vertically aligned plasmonic nanorods and covered with a rather thick (2 μm) dye-doped polymer film (Figure 12a). With the increase of the pulse energy of the pump laser, the spectrum of the emission experienced narrowing, while the output power dependence had a typical s-shape, characteristic to the initial onset of ASE and then transition to a lasing regime. Although a particular mode participating in lasing and the exact feedback mechanism were not determined, the lasing is probably related to the bulk plasmon modes of the hyperbolic metamaterial layer.^205,207^ The developed theoretical model allowed determination of a β-factor of the laser (showing the fraction of spontaneous emission going into the lasing mode; the higher, the better), which was found to be quite high (equal to 0.23), which generally leads to a low lasing threshold. A 35% lower lasing threshold and twice higher lasing power were observed in comparison with the nanorod metamaterial in an elliptic dispersion regime at the same emission wavelength (the dispersion was controlled by setting the nanorod diameters at the fabrication stage), while no lasing was observed for metallic films or multilayer hyperbolic metamaterials. Theoretical investigation of the phenomenon revealed the relation of the lasing threshold to the thickness of the metamaterial.^207^ As a final note, although enhanced ASE^205^ and lasing^206^ have been clearly shown in the above two articles, their relation to the nonlocal character of the effective optical properties of the metamaterial was not explicitly demonstrated and requires further investigations.

The possibility of using a multilayer hyperbolic metamaterial slab in hypercrystal configurations alternated with gain layers to achieve nonreciprocal transmission or unidirectional invisibility^208^ and to realize a DFB-type laser^209^ has also been theoretically discussed. Lasing in the cavity configuration containing an HMM and multiple quantum wells as the gain medium has been experimentally demonstrated,^210^ together with random lasing in disordered layers of gain-supplying NaYF_4_:Yb/Er/Tm@NaYF_4_:Eu core–shell nanoparticles covering a hyperbolic metamaterial slab.^211^

To stimulate experimental research and provide an explanation of the observed results, loss compensation via the incorporation of gain in the bulk of hyperbolic metamaterials, e.g. by doping the dielectric slabs in the multilayer hyperbolic material with dye molecules, was extensively theoretically studied. Employing various analytical and numerical approaches, it was found that when the dye is in the completely saturated state, the loss in the direction perpendicular to the layers can be fully compensated and even overcompensated, while the compensation in the orthogonal directions is generally beyond the achievable values of dye-facilitated gain.^213^ The influence of a partial loss compensation on the dispersion and negative refraction in hyperbolic metamaterials provides a possibility to switch between different propagation regimes.^212,214^ If the thicknesses of the layers are adjusted to be the same, then according to the EMT, the permittivity components ε_∥_ and ε_⊥_ can be simultaneously tuned to zero and infinity, respectively, so the metamaterial enters a so-called epsilon-near-zero-and-pole (ENZP) or canalization regime. Its interesting feature is that, at the spectral position at which the above condition is satisfied, the metamaterial switches the hyperbolic behavior from type I to type II. To realize this, the real parts of the permittivities of the metal and dielectric should be equal but oppositely signed (see eqs 4 and 5), which can be adjusted by the proper choice of the operation wavelength and/or materials. Then, the presence losses start to play the leading role, deteriorating the metamaterial performance, and adding a gain in the dielectric layers is a solution to bring the metamaterial close to the ideal singular optical response. In this regime, the optical signal, propagating along the metamaterial optical axis (perpendicular to the layers), is amplified. With reduced damping, multilayer metamaterial slabs with certain thicknesses have pronounced Fabry–Perot resonances (Figure 12b) at which the transmitted power is significantly higher than the power of the incident light. Furthermore, due to the strictness of the resonant condition, the spectral width of the amplified transmission was just 1 nm. One needs to stress here that this was achieved with a routinely used density of the doping dye (with a technological problem remaining, though how to achieve the required uniform gain). Furthermore, in the same way as was demonstrated for ENZP metamaterials with no gain,^215^ the gain-assisted “ideal” metamaterial canalized a light beam propagating along its optical axis into a narrow nondiffracting beam. On the basis of this phenomenon, an amplifying perfect lens was numerically demonstrated. Moreover, the implications of the gain-compensated singular optical properties of such a metamaterial were argued to go far beyond the enhancement of the optical signal and extend to the realization of spectral singularities, active nonlinear cavities, and novel lasing devices. Finally, through the implementation of an interplay between gain and loss, 2D hyperbolic metasurfaces for propagating SPP waves were theoretically demonstrated.^70^

### 5.3 Incoherent Light Sources and Lasers Based on Plasmonic Crystals

**Figure 13:**
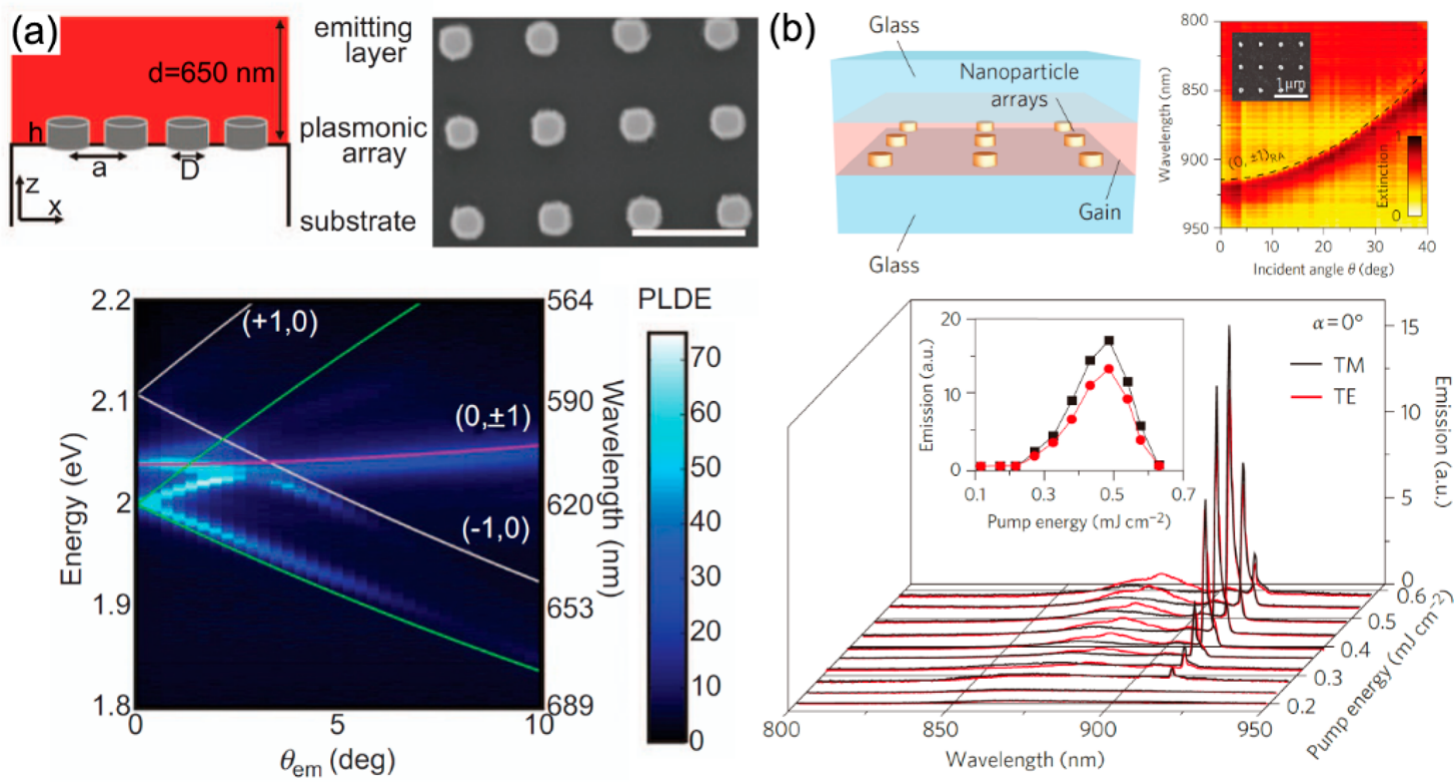
Spontaneous emission enhancement and lasing in dye-functionalized plasmonic crystals. (a) Schematics and an SEM image of the plasmonic crystal; the graph shows the achieved fluorescence enhancement (*p*-polarized detection), relevant to the excitation of the collective plasmonic modes (plotted with colored lines). Adapted with permission from ref (217). Copyright 2013 Springer Nature. (b) Schematics, an SEM image, and a dispersion diagram of the plasmonic crystal; the graph and inset present the spectra of the lasing signal and the magnitudes of the participating modes as a function of the pump pulse energy, respectively. Adapted with permission from ref (221). Copyright 2013 Springer Nature.

Although plasmonic crystals (Figure 13), which are an analogue of planar photonic crystals for SPP waves, are intrinsically based on diffractive effects and, therefore, strictly speaking do not belong to the class of metasurfaces, they present a stepping stone to achieving incoherent light sources and lasers based on metasurfaces. Plasmonic crystals present a unique platform for obtaining gain-assisted emission with very small spectral widths and very high directivity. Both incoherent light sources utilizing the enhancement of spontaneous emission and lasers making use of a Bragg-assisted feedback were demonstrated on the basis of plasmonic crystals.

We initially consider incoherent light sources,^216−220^ which are important in many applications, such as solid state lightening, fluorescence microscopy, data communications, quantum computing, and others. The general idea behind using the plasmonic crystal platform is the following. If the crystal, formed by an array of metallic nanoparticles or nanoholes in a metal film, is illuminated with light, for each wavelength there is a set of angles at which the diffraction orders propagate exactly along the plane of the array, corresponding to the points of disappearance of the diffraction orders producing Rayleigh anomalies in reflection. For slightly larger incidence angles, the increased in-plane wave vector leads to the coherent excitation of collective plasmonic modes of the array, which might be either plasmonic or nearly free-space photonic modes, depending on the strength of the interparticle coupling. The prominent feature of these modes is that, having a resonant character, they can have a very narrow excitation angle range. When an excited fluorophore is placed in the vicinity of the crystal, it emits light into these modes, resulting in the emission of each wavelength in the free space at the same angle. From this perspective, the collective plasmonic excitations are quasi-bounded leaky modes radiating into photons. The increased density of optical states associated with the modes leads to the decrease of the spontaneous lifetime and increase of the fluorescence signal, which results in faster and brighter light sources. At the same time, the high directionality of the emitted signal is achieved via the large in-plane spatial coherence of the collective plasmonic mode. In the experiments, plasmonic crystals are typically functionalized with dye molecules in a polymer matrix, which are pumped by CW lasers or LEDs.^216−220^ Particularly, for unpolarized emission at the normal direction, fluorescence enhancement of 60 times was achieved at the resonant wavelength of the plasmonic mode and 14 times for the signal integrated over the entire spectrum of the dye (Figure 13a).^217^ The divergence angle of the directional emission can be as small as 1.5°. High quantum efficiency of the dye is only weakly altered by the plasmonic structure, therefore,the high values of the fluorescence enhancement are attributed to the plasmonically assisted enhancement of the pump and the high directionality of the emission. For emitters with low quantum efficiencies, the plasmonic structures can additionally improve the efficiency by providing a high density of local optical states available for emission.^216,217^

On the other hand, it is interesting to look at the high directionality of the emission from another perspective: it actually means the large spatial coherence of the collective mode across the crystal (a wide, well-defined phase-profile of the emitting source is needed to produce a beam with high directionality). By varying the coupling of the dye molecules to the collective modes from weak to strong coupling regimes, it was found that, even in the latter case, when the mode is predominantly exitonic-like, the spatial coherence of the mode spans over several micrometers.^176^ Engineering the shape of metallic nanoparticles and spacing allows the control of the dispersion of the collective plasmonic modes and, therefore, the spectral and directional characteristics of the obtained light source.

To achieve lasing with dye-functionalized plasmonic crystals, two additional conditions need to be met. The first one is a much higher level of pumping required for the population inversion, which is realized with pulsed pump lasers. The second one is the presence of a feedback mechanism, required for the setting up the lasing modes, which is introduced by a cavity in the case of traditional photonic lasers or by a plasmonic resonance, which is the case of spasers (localized surface plasmon lasers, emitting into dark plasmonic modes). In plasmonic crystal lasers, similarly to distributed feedback (DFB) photonic lasers, the feedback mechanism is based on the periodicity of the structure.^221−230^ Particularly, only the modes corresponding to standing waves, located at the Bragg band-edge points on the plasmonic crystal dispersion, will enter a lasing regime. Strong scattering of the plasmonic modes on the meta-atoms provides an excellent mechanism for their formation. By increasing the pump fluence and crossing the lasing threshold for one of the Bragg band-edge modes, the formation of lasing with a 1.3 nm bandwidth and a divergence angle of just 1.3°, which always happens near the normal direction, as the result of the diffraction-assisted coupling of the plasmonic mode to the output light beam, was shown (Figure 13b).^221^ As was explained above, the strong directionality of the beam means large spatial coherence of the collective plasmonic band-edge mode, which was estimated to extend over a 50 μm distance. The spatial coherence of the mode can extend over a millimeter scale, while the temporal coherence can reach 2 ps.^231^ In plasmonic crystals, spontaneous emission mostly happens in the vicinity of the plasmonic nanoparticles and, therefore, is greatly facilitated by the high LDOS provided by them, both at the emission frequency, supplying a higher LDOS to emit into, and at the pump frequency, providing the excitation field enhancement.^231^ Ultrafast time-resolved fluorescence measurements confirmed up to a 200-fold decrease of the excited state lifetime in the vicinity of the nanoparticles, corresponding to the same increase of the LDOS. A superior performance of the dye-functionalized plasmonic crystals over their dielectric counterparts was also explicitly experimentally shown. The studies were extended to randomized and quasi-periodic structures.^224,227,232,233^

Plasmonic crystal lasers of a finite size demonstrate a more developed modal structure, corresponding to the standing wave cavity modes defined by the array dimensions.^228^ Based on this effect, multimodal plasmonic lasers with an engineered emission wavelength were realized, as each mode corresponds to a certain emission wavelength, defined by the collective mode dispersion and the resonant cavity wave vectors. The obtained nonzero resonant (in-plane) vectors correspond to different angles of the output emission for various laser modes. By varying the size of the nanoparticles and through this the near-field distribution of the lasing modes and pumping, it is possible to engineer the competition between the modes and, therefore, their output powers. Time-correlated photoluminescence measurements showed the possibility of an ultrafast plasmonic crystal laser operation at a picosecond time scale. Lasing was also demonstrated for plasmonic crystals realized with the use of periodic patterned metallic films, where it was based on standing SPP waves.^225^

It is interesting to note that lasing in dye-functionalized plasmonic crystals does not necessarily happen with the bright modes, coupled to the far-field radiation. Even more intriguing is that a dark mode, positioned at the top edge of the band gap, can actually win the mode competition, as it has smaller absorptive losses due to the minima of its field at the nanoparticle positions.^229^ The outcoupling to the far-field radiation can happen in this case through a mechanism related to a finite size of the array, and thus, the mode will dominate in the lasing spectral profile. This mechanism is based on the gradual evolution of quadrupolar nanoparticle resonances, with the minima of the fields at the nanoparticle centers, observed in an infinite array (and in the center of the finite-size array), to the dipolar one toward the edges of the finite array with a 50–100 μm size. Additionally, such a mode was demonstrated to have a very narrow 0.2 nm spectral width and 0.3° directionality, indicating that its spatial coherence extends over the entire array. The dark mode strongly coupled to the molecular transition, resulting in plasmon-exciton character of lasing was also observed.^175^ Since the metallic particles forming the plasmonic crystal in this case were nanorod-shaped and the emission from the bright (diploar) mode is polarized across the nanorods while the emission from the dark (quadrupolar) mode polarized along the nanorods, the onset of lasing was clearly seen in the switching of the polarization of the emission when the pump power crossed the threshold. When the band-edge Bloch modes spectrally overlap with the localized plasmonic mode supported by individual nanoparticles forming the array,^230^ the LSP resonance crosses the band-gap, and the lasing mode primarily contributing to the output signal switches from being at the bottom edge of the band gap to the top edge. Plasmonic crystal lasing with dynamic spectral tuning was also realized with a real-time change of dye solutions, interfacing the plasmonic crystal, with varied refractive indices, which modifies the frequencies of the band gaps and, therefore, the emission wavelength.^226^

## 6 Control of Light with Plasmonic Metamaterials Functionalized with Molecular Media

**Figure 14:**
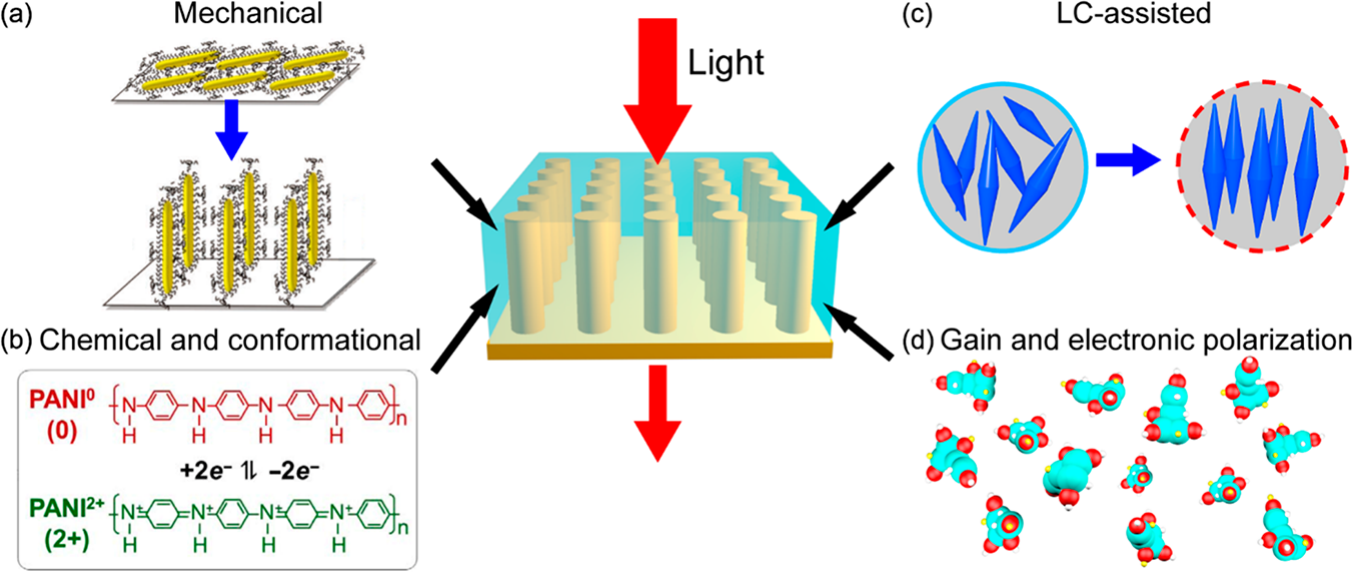
Control of the metamaterial optical response using various approaches based on: (a) molecular-assisted mechanical reconfiguration of the metamaterial, (b) chemical or conformational changes in the molecules, (c) phase transitions in liquid crystals, and (d) electronic nonlinearities related to gain and electronic polarization in molecular media.

Modulation of light using metamaterials with an actively controlled optical response can be implemented using various methods (Figure 14). Particularly, the optical properties of metamaterials can be modified through their mechanical reconfiguration (modifying the geometry of the meta-atoms and/or their spacing) or changing the optical properties of constituting materials using a variety of physical approaches. In the case of plasmonic metamaterials, both these methods will lead to the modification of the related plasmonic resonances and, therefore, the metamaterial optical response. In this section, we discuss the control of light using active plasmonic metamaterials with molecular-assisted modulation mechanisms.

### 6.1 Mechanically-Reconfigurable Plasmonic Metamaterials

**Figure 15:**
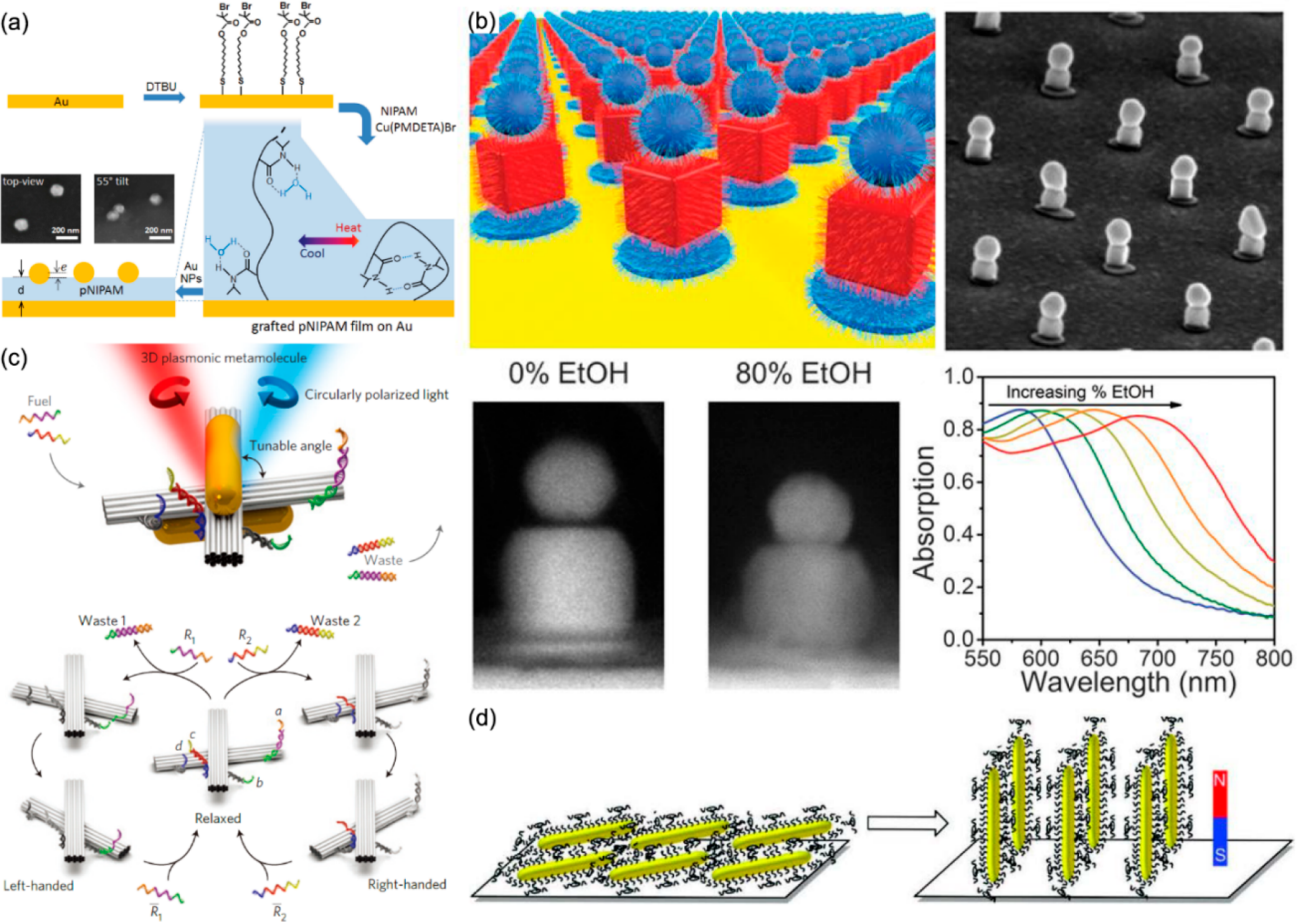
Molecular-assisted structural modulation of the optical response of metamaterials and metasurfaces. (a) Tuning the optical properties of a nanoparticle-on-mirror plasmonic system via molecular reconfiguration of the gap material (pNIPAM, *d* = 67 nm in the collapsed state). Reprinted with permission from ref (237). Copyright 2016 WILEY-VCH Verlag GmbH & Co. KGaA. (b and c) Tuning optical absorption (b) and CD of a metamaterial (c) with a chemically assisted DNA modification. Adapted with permission from ref (242). Copyright 2018 American Association for the Advancement of Science (b). Reprinted with permission from ref (244). Copyright 2014 Springer Nature (c). (d) Control of the optical properties of LC-functionalized nanorod metamaterials through reorientation of the nanorods due to the action of the magnetic field on the LC-ligands. Reprinted with permission from ref (245). Copyright 2013 WILEY-VCH Verlag GmbH & Co. KGaA.

As was explained above, the optical properties of a metamaterial or a metasurface depend on the geometry of their meta-atoms and the spacing between them. Therefore, engineering the metamaterial structural parameters offers a straightforward approach to tune the metamaterial optical response. In the case of a metasurface, probably the easiest way to realize this is by placing the meta-atoms on an elastic, e.g. polymer, substrate and changing their geometrical parameters and spacing upon mechanical stretching or electromechanical reconfiguration,^234^ influencing the electromagnetic coupling between the meta-atoms and their plasmonic resonances. Implementing mechanical reconfiguration, a meta-lens with a variable focal distance^235^ and a variable-image hologram^236^ were demonstrated. This opens an opportunity for a more advanced metasurface tuning based on reversible shrinkage of the substrate material upon thermal stimulation, stemming from the spatial reconfiguration of the constituting molecules. Particularly, poly[*N*-isopropylacrylamide] (pNIPAM) polymer offers a reversible volume variation in two or even more times upon the change of its molecular structure from hydrophilic (swollen) to hydrophobic (collapsed) at the critical hydration temperature (Figure 15a).^237^ This was used to tune a plasmonic resonance of a nanoparticle-on-mirror system submerged in water via the variation of the coupling strength between the particle and the metallic film with the heating-assisted change of the thickness of a pNIPAM film placed between them. Furthermore, it was demonstrated that the change of the optical response can be achieved by optical means through the local heating of the plasmonic system produced by a pump pulse. The switching times in this case were shown to be less than 2 μs, which was the limit of the temporal resolution in the experiments. A similar approach based only on the structural changes of the polymer was implemented for a pNIPAM-functionalized array of bow-tie antennas^238^ and hexagonal arrays of gold nanoparticles.^239^ In the latter case, upon the transition between the two structural states of the polymer molecules in a water environment, both the thickness (between 150 and 25 nm) and refractive index of the polymer (between 1.37 and 1.43) were changed, which led to the shift in the plasmonic resonance of the metasurface from 671 to 680 nm. In principle, a polymer-assisted mechanical reconfiguration can be induced directly by optical means^240^ or by application of a magnetic field.^241^

A breakthrough in this area came with the development of DNA-assisted techniques, in which DNA molecules are used both for fabrication of metamaterials and for their active tuning.^242,243^ Specifically, large-area multiparticle plasmonic metamaterial arrays were fabricated through a self-assembly of nanoparticles in periodically structured PMMA templates, when each next nanoparticle was functionalized with DNA strands complementary to that of the previous one, assuring their efficient attachment to each other (Figure 15b).^242^ When the template was dissolved, the meta-molecules, formed by the nanoparticles (meta-atoms), remained stable. Furthermore, they can interact with the surroundings, which was used for reversible tuning of their geometrical parameters and, thus, the optical response of the metamaterial by a chemical means. With a change of the solvent polarity, when its composition was changed from 0 to 80% of ethanol in a H_2_0/NaCl solution and back, the oligonucleotide bonds experienced contractions and expansions from >12 nm to <3 nm. This led to the modification of the electromagnetic coupling between the nanoparticles constituting the metamolecule and, therefore, its optical response. The optical absorption of the metamaterial was reversibly tuned from 14% to 73% over the entire visible spectrum.

Combinations of plasmonic and molecular functionalities within individual meta-molecules were developed using DNA molecules.^244,246−248^ Two plasmonic meta-atoms (gold nanorods) formed a meta-molecule and ensured strong interaction with light, while the molecular components (DNA) both provided the meta-molecule frame and acts as active elements causing the meta-molecule rotational reconfiguration (Figure 15c).^244^ Particularly, chemically induced locking and unlocking of the meta-molecule in the states with different chirality allowed control of the CD of the solutions containing the nanostructures. The approach was further extended to realize an optical control of the meta-molecule chirality, when the lock of the state was implemented with a use of photosensitive azobenzene molecules connected to the two arms of the meta-molecule.^248^ Under illumination with visible light, the molecules experience transition from *trans* to *cis* configuration in which the molecules can be hybridized into a locked state, switching the meta-molecule into a chiral state. UV illumination initiates the reverse process releasing the meta-molecule to the initial achiral form. The meta-molecule solution can then be reconfigured between the states with zero and substantially nonzero CD.

A possible alternative way to reversibly modify the optical response of meta-atoms through their structural changes is a voltage-induced formation of a conductive filament^249−251^ via ionic transport or electrodeposition.^252^ An interesting approach to structuring and tuning of plasmonic metamaterials via a molecular-assisted reorientation is the functionalization of metallic components with liquid crystals (LCs) and using their property of self-orientation with respect to the applied external fields. Particularly, gold nanorods were capped with nematic LC shells, and magnetic fields were used for their axial alignment in a bulk metamaterial^253^ or side-by-side packing into hexagonal arrays to form a metamaterial layer^245^ (Figure 15d). This results in a highly anisotropic structure (with a possible hyperbolic dispersion in the latter case) and a striking difference in the optical response to the initial isotropic medium. Similar orientation-assisted tuning can be achieved with an electric field with the aid of a LC host medium^254−256^ or directly through the induction of dipole moments in the plasmonic nanostructures themselves.^257^

### 6.2 Plasmonic Metamaterial Tuning with Molecular Transformations

**Figure 16:**
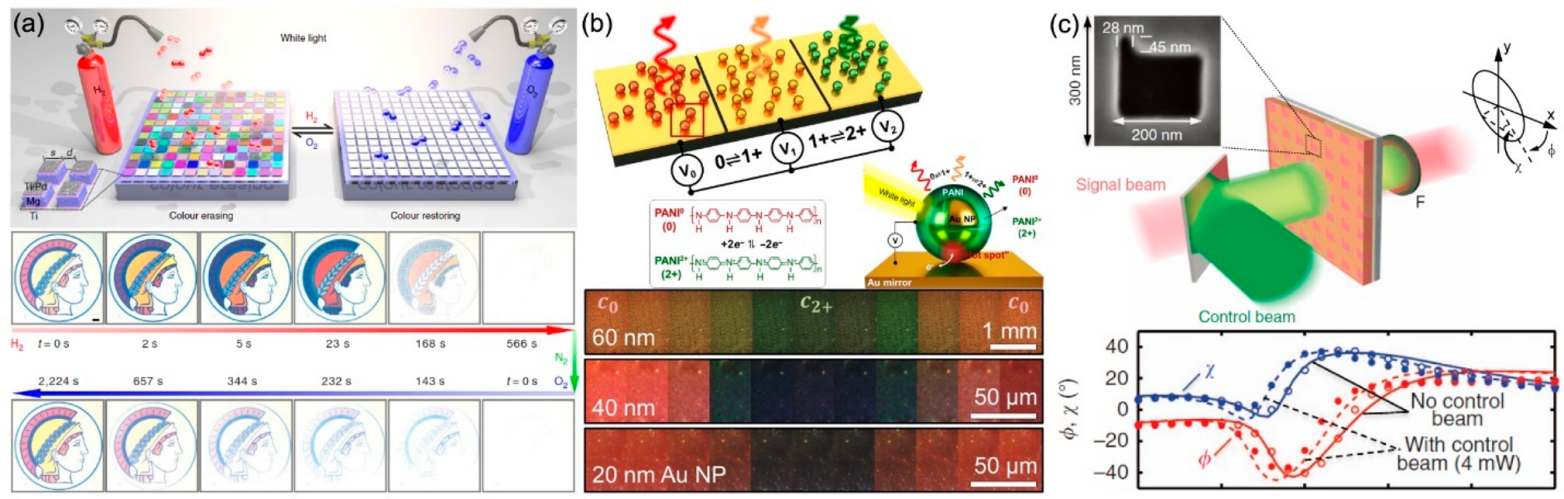
Modulation of the optical response of metamaterials and metasurfaces using molecular transformations. (a) Dynamic switching of metasurface colors by hydrogenation/oxidation of the constituting magnesium nanoparticle arrays. Adapted with permission from ref (260). Copyright 2017 Springer Nature. (b) Electrochemical control of pixel colors based on arrays of plasmonic nanoparticles functionalized with PANI. Adapted with permission from ref (265). Copyright 2019 American Association for the Advancement of Science. (c) Modulation of a polarization state of the transmitted light via a photoinduced structural transformation in an ethyl-red doped PMMA film coated onto a chiral metasurface. Adapted with permission from ref (266). Copyright 2017 Springer Nature.

The second principal way to tune the optical response of a metamaterial or a metasurface is changing the optical properties of the constitutive materials. We start with its implementation based on chemically induced modifications. When exposed to hydrogen atmosphere, some metals, such as palladium, yttrium, and magnesium, allow penetration of hydrogen atoms into their crystal lattices and undergo chemical reactions to become metal hydrides, which leads to dramatic changes in their optical properties. Importantly, this reaction happens at room temperature, which makes this phenomenon attractive for practical applications. Magnesium is a particularly interesting element in this sense, as in the metallic phase it is a plasmonic metal, whereas its hydride MgH_2_ is a dielectric. This property was used to realize chemically reconfigurable metasurfaces with resonances spanning the visible spectrum to obtain various colors in reflection and encode images by selective patterning which can be switched upon exposure to a hydrogen environment^258−261^ (Figure 16a). The process can be reversed by exposure of the metasurface to oxygen, which reacts with the hydrides, binding/removing the hydrogen atoms and restoring the metallic states. Such conversion can be repeated many times, before stress-related changes deteriorate the structure performance;^258^ however, the overall switching time is slow and takes 100s of seconds in either direction. The metasurface, defined by the exposure times or H_2_ and O_2_ concentrations, can be “frozen” at any stage by switching to a nitrogen atmosphere. This is contrary to the hydrogenation processes of yttrium and palladium, which are volatile, so that the metallic states are self-restored after the hydrogen atmosphere is removed. Apart from hydrogenation, redox reactions in silver (converting it between silver and AgCl)^262^ and H^+^/O^–^ ion implantation in TiO_2_ (converting it between normal and “black” versions, having a 1 eV difference of the band gap and, therefore, varied absorption)^263^ or electron and Li^+^ migration into WO_3_ (changing the carrier density)^264^ can also be used for reversible tuning of the metamaterial response.

Electrochromic polymers are attractive candidates for active media in dynamically tuned metamaterials due to their ease of deposition, mechanical flexibility, stable optical performance, and wide tunability in the visible spectral range. One of them, polyaniline (PANI), offers a drastic electrochemically induced refractive index change from a reduced dielectric state to an oxidized conductive state.^265^ It has been widely used in plasmonic metamaterials of various configurations, such as polymer-coated metallic nanoparticle arrays produced by electron beam lithography or ion beam milling,^267−269^ and monolayers of disordered polymer-functionalized metallic nanoparticles.^265,270^ Although the former methods can be replaced by more affordable nanoimprint techniques, the latter approach has an advantage of scalable fabrication using a low-cost self-assembly method “as is”, which is complemented by the fact that the nanoparticles with a PANI coating of a variable thickness can also be manufactured by a bottom-up technique.^265^ Implementing it on the surface of a metallic film, disordered nanoparticle-on-mirror assemblies were obtained with optical properties defined by out-of-plane and in-plane surface plasmon resonances due to coupling between the nanoparticle and the metallic film (Figure 16b). The out-of-plane resonance, having strong field localization in the polymer-filled gap between the metallic nanoparticle and film, shows a very high sensitivity to the optical properties of PANI. When PANI was electrochemically converted between reduced and fully oxidized states by the application of a variable voltage (−0.2 to 0.6 V), the out-of-plane resonance was reversibly shifted by Δλ = 60 nm and the color of the nanoparticle-on-mirror pixels in the scattered light was changed from red to green. The highly localized nature of the resonance has the advantage of angle-independent optical performance and enables the realization of an extremely small pixel size approaching the diffraction limit. The switching was completely reversible with possible modulation rates of 10s of Hz and energy consumption of just 9 fJ per pixel. Alternative electrochromic polymers, whose optical properties can be switched electrochemically or chemically between conductive and insulating states to obtain dynamic metamaterials, are poly[3,4-ethylenedioxythiophene] (PEDOT)^271−273^ and poly(3,4-propylenedioxythiophene) (PProDOT)^269^ derivatives, poly(thieno[3,4-*b*]thiophene),^274^ polypyrrole (PPy),^275−277^ and triphenylamine-based polyamide (TPA-PA).^278^

Photochromic dye-doped polymers received significant attention as active materials for optoelectronic devices due to optically induced high refractive index changes together with ease and versatility of fabrication.^279,280^ Therefore, it is not surprising that they were implemented in active metamaterials (Figure 16c).^266^ In one example, a metasurface based on a periodic array of L-shaped openings in a 100 nm thick metallic film was coated with PMMA polymer doped with ethyl red dye. As the openings are chiral, the linearly polarized light transmitted through such a metasurface acquires an elliptical polarization characterized by an ellipticity angle χ and an azimuth rotation angle ϕ (the linear polarization can be represented as a sum of LCP and RCP states, which upon transmission with different amplitude and phase coefficients results in an elliptical polarization state). When the metasurface is illuminated by a green pump light, the ethyl red molecules experience a structural transformation from *trans* to *cis* configuration, which changes their polarizabilities and modifies the refractive index of the doped polymer. This affects the optical response of the metasurface and results in a modification of the polarization state of the transmitted light, with up to a 20° change in the polarization angles for just a 4 mW pump power (6.3 kW/cm^2^ intensity), particularly pronounced in the spectral region of the metasurface plasmonic resonance. When the optical stimulation is withdrawn, the ethyl red molecules gradually experience the reverse structural transformation to the *trans* state. The temporal characteristics of the polarization state modulation were found to follow a biexponential time dependence with fast and slow components of <1 and ∼10 ms, respectively. Using a spatially structured pump beam, resulting in inhomogeneous photoisomerization across the metasurface, an optically controlled spatial light modulator was demonstrated.^281^

The performance of active metasurfaces functionalized with dye-doped polymers can be enhanced with the use of Fano-type plasmonic resonances^282^ and strong coupling between the plasmonic resonances and dye excitons.^283,284^ An alternative approach to dye-functionalized active metasurfaces utilizes the fluorescent properties of dyes.^285^ Here, the metasurface-enhanced emission of the dye is controlled by the modulated pump light, closely following its profile with picosecond time resolution, leading to the modulation bandwidth exceeding 14 GHz. Modulation of light and harmonic generation with the use of ultrafast electronic nonlinearities will be discussed in detail in subsection 6.5.

### 6.3 Active Metamaterials Functionalized with Liquid Crystals

**Figure 17:**
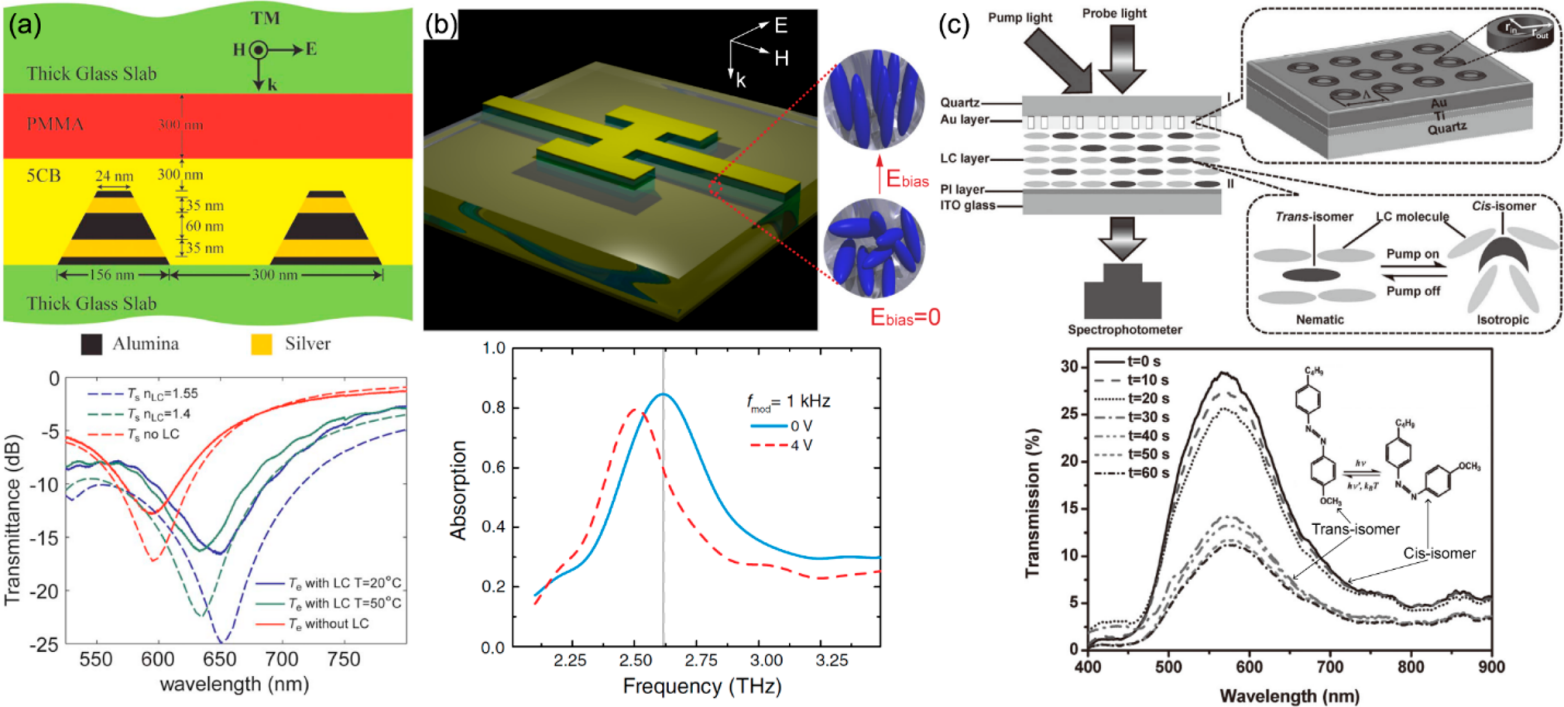
Active plasmonic metamaterials/metasurfaces functionalized with liquid crystals, featuring (a) thermal, (b) electrical, and (c) optical control. Adapted with permission from ref (286). Copyright 2009 American Institute of Physics (a). Adapted with permission from ref (289). Copyright 2013 American Physical Society (b). Adapted with permission from ref (297). Copyright 2012 WILEY-VCH Verlag GmbH & Co. KGaA (c).

Tuning of the optical properties of metamaterials with the use of LC-assisted mechanical reconfiguration was considered above in subsection 6.1; here we overview a classical approach for modulation of an optical response of LC-functionalized metamaterials through the induced changes in the LC refractive index. In this respect, LCs present an attractive choice of active materials, offering large modifications of the refractive index upon a phase transition, which can be induced with thermal,^286,287^ electrical,^288−295^ optical,^296,297^ or acoustical^298^ stimulations. They possess a wide tuning range of the refractive index over a broad spectral interval from visible to microwave frequencies and have low energy consumption together with high transmittance, which make them a very versatile active material platform.^291,299,300^ Strong modification of the optical response of a magnetic metamaterial functionalized with 5CB LC in a THz spectral range was demostrated (Figure 17a).^286^ The metamaterial consists of an array of parallel metallic strip pairs, producing a resonant magnetic response, which can be used to achieve an effective negative magnetic permeability. In particular, light incident on the metamaterial induces in the strips antisymmetric currents, which together with the displacement current form a closed loop. With a proper choice of a phase response near the resonance, a counter-directed magnetic field with respect to the driving one can be induceed, resulting in a negative effective permeability of the metamaterial. In the transmission spectra, such a magnetic resonance is observed as a dip. The space between the nanostrip pairs was infiltrated with LCs, which at 20 °C are in a nematic phase with molecules oriented in the plane of the sample and perpendicular to the paired-strip lines. When the temperature is increased above 35 °C, the LC experiences a phase transition to the isotropic phase, which leads to a refractive index change of Δ*n* = 0.15 and a spectral shift of the magnetic resonance, resulting in a substantial change of transmission. The modulation was shown to be fully reversible and repeatable.

The classic approach to modification of LC optical properties is an electric stimulation by application of a constant or oscillating electric field.^289−295^ Using this approach, an efficient active metamaterial absorber at THz frequencies was demonstrated (Figure 17b).^289^ The metamaterial consists of an array of coupled SRRs connected by wires, which facilitate the application of a voltage between them and a metallic film below. The SRRs are excited by an electric field component of incident light charging the capacitors, while the split metallic loops produce inductance, which overall results in a standard L-C resonance and gives an opportunity to tune the effective permittivity of the metamaterial in a broad spectral range. On the other hand, the two-layer geometry (the resonator layer and the ground metallic film) facilitates resonant coupling to the magnetic field and the related engineering of permeability, through the same mechanism as described in the previous paragraph. Overall, the effective permittivity and permeability can be designed to match the impedance of the free space, which results in almost zero reflection and total absorption through the losses in the metallic components. The resonators were surrounded by 5CB LC molecules from three sides, apart from the gap separating them from the metal film, which was filled with polyimide. Upon the application of the voltage modulated at various frequencies between the resonators and the ground metallic film, the LC molecules become oriented along the lines of the electric field, which leads to a substantial change in the optical properties. This results in a 4% shift of the metamaterial absorption resonance and a 30% modulation of the absorption at 2.62 kHz rate. In various metamateril geometries, such electric modultion was realized in various spectral ranges and can find applications in switches and special light modulators.^289,290,295,300−304^ Beam spatial modulation and steering devices,^305,306^ polarization converters,^307,308^ and displays^291,293,309,310^ with operational speeds up to 40 kHz have also been achieved.^300,306,307,311^ Additionally, control of the permeability of an LC-functionalized metamaterial with a magnetic field has also been demonstrated.^312^

All-optical modulation of LC-based metasurfaces offers the benefit of remote control and ease of selective modulation of chosen metasurface areas. Exploring this possibility, an active metasurface was demonstrated, based on annular aperture arrays in a metal film interfaced with a 4-butyl-4-methyoxyazobenzene (BMAB) photochromic LC molecules (Figure 17c).^297^ The transmission peak in the wavelength range of 500–600 nm corresponds to a coherent interaction of cylindrical surface plasmons excited inside the annular apertures and propagating surface plasmon polaritons excited on the metal film surface. Under illumination with UV light, *trans*-isomer elongated BMAB molecules, which are initially in a thermally stable, in-plane oriented nematic phase, are converted to a bent *cis*-isomer, which disrupts the order, finally resulting in an isotropic LC phase. This is accompanied by a change of the refractive index of the order of 4% which is sufficient to substantially decrease the transmission by more than two times, happening at a time scale of ∼10 s. Under illumination with visible light, BMAB can be transformed back into the *cis*-form, which makes the optical modulation completely reversible. Similar functionality was demonstrated with the LC-functionalized metasurfaces based on double-split ring resonators.^296^

### 6.4 Modulation of Metamaterial Optical Response with Gain

Introducing materials with optical gain in the design of metamaterials offers another efficient means to implement all-optical control of their optical properties and also achieve unusual parity-time (PT) symmetric (non-Hermitian) functionalities. These functionalities are related to the interplay between gain and loss and can lead to entirely new and unexpected features for light control.

**Figure 18:**
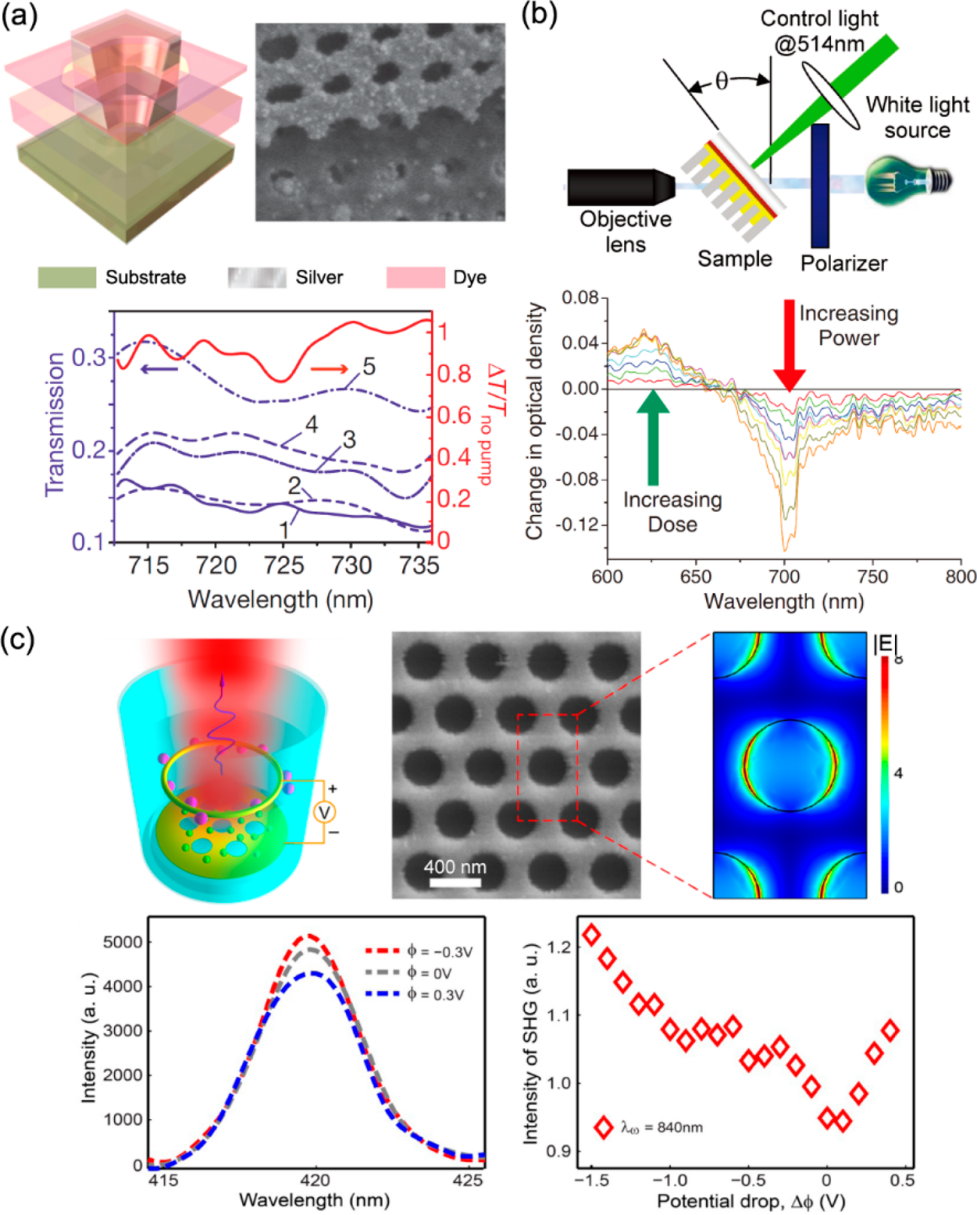
Nonlinear metamaterials employing gain and molecular electronic nonlinearities. (a) Modulation of metamaterial transmission through the introduction of gain in a constituent Rh800 dye: (line 1) without pumping, (lines 2–5) at different pumping intensities, (red line) the achieved transmission modulation. Adapted with permission from ref (197). Copyright 2010 Springer Nature. (b) Experimental setup and the change in the transmission of a plasmonic nanorod metamaterial functionalized with a nonlinear polymer (poly-3BCMU) at different control light intensities (0–5.5 mW/cm^2^). Adapted with permission from ref (313). Copyright 2008 The Royal Microscopical Society. (c) Electrically controlled SHG from a hole-array plasmonic crystal placed in an electrolyte: (top) schematics, SEM image and the electric field distribution near the holes; (bottom) the spectral profile of the SHG signal at different voltages (left) and the dependence of the SHG on the control voltage (right). Adapted with permission from ref (314). Copyright 2016 American Chemical Society.

By introducing a gain medium in the metamaterial and dynamically varying the amount of gain through pumping, one can control the quality factors of the metamaterial plasmonic modes and, therefore, the optical response of the metamaterial. This approach was used to modulate the optical properties of a fishnet metamaterial functionalized with epoxy doped with a dye (Figure 18a).^197^ The double-layer fishnet structure offers an effective negative refractive index provided by a negative real part of the effective permittivity and a negative real part of the effective permeability of a double-layer metallic structure of crossed arrays of strip-pair lines (the crossing is introduced to achieve polarization insensitive performance at normal incidence). If gain is introduced through hybritisation with active molecules, the optical properties of the metamaterial can be significantly improved through the compensation of the metallic loss. Pump–probe experiments together with the numerical analysis show that the quality factor of the magnetic resonance of the metamaterial substantially increased and more pronounced negative values of both the effective permittivity and permeability ere achieved. This results in the all-optical control of the transmission through the metamaterial layer with a modulation depth approaching 100% (Figure 18a). The high level of the gain in the dye-doped epoxy nanolayers, much higher than in the case of the bulk counterpart, was provided by large local enhancement of the excitation field.

Other approaches based on gain-assisted modulation of absorbance,^315^ including coherent control^316^ and plasmon-induced transparency,^317^ were introduced. By exploiting non-Hermitian effects due to the interplay between gain and losses, it is possible to achieve very strong circular birefringence and CD.^318^ The interplay between the PT symmetry and the non-Hermitian physics can find numerous applications in, e.g., sensing,^319^ optical cloaking,^320^ lossless guiding,^321^ active components^322,323^ for electromagnetic field manipulation and imaging,^324^ and nonlinear^325^ and quantum optics.^326,327^

### 6.5 Metamaterials with Molecular Electronic Nonlinearities

Plasmonic metamaterials have been widely developed for nonlinear optical applications using intrinsic nonlinearities related to a free-electron gas of plasmonic constituents.^15,19,44,62,65^ Both Kerr-type and coherent (harmonic generation, four-wave mixing) nonlinearities have been engineered and enhanced through metamaterial designs. Traditional second- and third-order nonlinearities based on the electronic response of a molecular medium can also be enhanced in the metamaterial environment and used for the modulation of light or nonlinear harmonic generation. In this respect, polymers are well suited for integration in metamaterials. Nonlinear polymers with a very strong third-order Kerr nonlinearity based on the high nonlinear polarizability of π-electron clouds were used for all-optical control of light in the visible spectral range using both metamaterials^313^ and plasmonic crystals.^328^ Particularly, plasmonic nanorod metamaterials were functionalized with poly(3-BCMU) nonlinear polymer UV-cured from a spin-coated monomer solution, and transmission of signal light through such hybrid metamaterial was modulated by illuminating it with a control light which induces refractive index changes of the polymer (Figure 18b).^313^ The strong modulation effect is underlined by two key advantages provided by the metamaterial: (1) large enhancement of the local pump intensity due to the electromagnetically coupled meta-atoms (nanorods) and (2) high sensitivity of the metamaterial transmission to minute changes in the refractive index of the surroundings. Both reversible changes due to optically induced nonlinear electronic polarization in the polymer and nonreversible changes due to continuing polymerization of the molecular medium were observed, associated with the strong field enhancement between the meta-atoms. The nonreversible changes happen mostly in the region of the absorption band of the polymer (around 625 nm), while the reversible changes are pronounced in the metamaterial ENZ region (around 700 nm), where the optical response is particularly sensitive to the changes in the refractive index.^329^ This approach was also realized in the case of plasmonic crystals, where all-optical modulation of the transmitted light signal accompanied by bistability of the transmission was observed.^328,330^

Ionic-assisted second-order nonlinear effects for the realization of nonlinear metasurfaces with electrically controlled SHG were also demonstrated (Figure 18c).^314^ SHG from a honeycomb nanohole array plasmonic (gold) crystal placed into a potassium sulfate (K_2_SO_4_) electrolyte solution was modulated. Since gold is a centrosymmetric material, the SHG is generated at the gold interface with the electrolyte. The application of a control voltage results in the accumulation of either K^+^ cations or SO_4_^2–^ anions near the interface, depending on the voltage polarity. The SHG efficiency modulation up to 150%/V was achieved.

## 7 Optical Sensing with Plasmonic Metamaterials

Sensors are widely exploited in modern technologies for the detection of events or changes in their local environment. Compared with their electric counterparts, optical sensors provide a number of advantages including high sensitivity, fast response, immunity to electromagnetic interference, safe operation in explosive or combustive atmosphere, and rich options for signal retrieval (e.g., optical intensity, spectrum, phase, and polarization). Benefiting from the strong subwavelength-scale confinement and enhancement of electromagnetic fields at the metal interface, surface plasmons are extremely sensitive to changes in the local dielectric environment of metallic structures and can greatly enhance Raman scattering and infrared absorption of molecules. Therefore, optical sensing has become one of the most representative applications of plasmonics since the first demonstrations of probing of electrochemical interfaces^331^ and detection of gases.^332^ Different from LSP-supporting metal nanostructures, the optical response (e.g., extinction and reflection spectra) of plasmonic metamaterials is determined not only by the plasmonic response of individual meta-atoms in the metamaterials but also by electromagnetic coupling between them. As a result, the optical response is highly sensitive to variations in the dielectric environment surrounding the meta-atoms, which also influences the coupling strength between them. This makes plasmonic metamaterials a particularly attractive platform for high-performance optical sensing applications. In this section, we review the applications of plasmonic metamaterials in optical sensing ranging from biochemical and gas sensing to surface-enhanced spectroscopy and chiral sensing.

### 7.1 Performance Characteristics of Plasmonic Sensors

The operational principle of the optical sensors is based on the modulation of the properties of a light wave reflected (transmitted) from (through) the sensor by a stimulus to be sensed. The detection can be realized monitoring the changes in wavelength, intensity, resonant angle of incidence, or phase. To evaluate the performance of the sensors, in addition to sensitivity, several benchmark characteristics were introduced such as bulk refractive index sensitivity, limit of detection (LOD), reproducibility, response time, and various figures of merit (FOMs), involving combination of parameters.

Sensitivity, which is expressed as the ratio of the change in the sensor output (e.g., resonance wavelength, light intensity, coupling angle) to the change in the quantity of the targeted analyte (e.g., its concentration), is a key performance characteristic of a sensor. Specifically, for plasmonic sensors, the sensitivity depends on both the bulk refractive index (RI) and local RI changes induced by the presence of analytes.^333^ The bulk refractive index sensitivity (*S*_*RI*_) is widely used to quantify the intrinsic sensing performance of a plasmonic system. In the case of plasmonic sensors monitoring the intensity changes at a fixed wavelength, it is defined as *S*_*RI*_ = Δ*I*/Δ*n*, where Δ*I* is the change of the scattered, transmitted, or reflected light intensity corresponding to the refractive index change Δ*n*. For the sensors based on the detection of the spectral responce, it is defined as the spectral shift of a plasmonic resonance peak, Δλ, with RI change: *S*_*RI*_ = Δλ/Δ*n*, where Δλ is the spectral shift of resonance peak. The distinguishability of the spectral shift of the resonance depends not only on the absolute value of the shift but also on the full-width at half-maximum (fwhm, *δλ*) of the resonance. Therefore, to compare the performance of different sensors to the changes of a bulk RI of the surroundings, a figure of merit FOM is introduced as FOM_λ_ = *S*_*RI*_/*δλ*.

The limit of detection is another important characteristic of a plasmonic sensor, which is defined as the minimum quantity (e.g., concentration) of analyte that can be detected by the sensor: LOD = 3σ/*S*_*RI*_, where σ is the standard deviation of the sensor output measured for a control sample without analyte which determines the system noise floor. Finally, a response time is determined by the time required for a sensor output to change from its initial state to 90% of its final settled value.

### 7.2 Biochemical Sensing

Optical biochemical sensors are highly required in various areas such as environmental monitoring, food safety, and disease diagnostics. Several types of such sensors have been developed employing the sensitivity of plasmonic response to refractive index changes in the local environment. Metal film-based SPP biochemical sensors can provide an extremely small LOD approaching ∼10^–7^ RIU.^12,334^ However, they are less sensitive to analytes with small molecule weights (e.g., <500 Da) due to the relatively weak confinement of electromagnetic field near smooth metal films, compared to the size of a molecule. In contrast, benefiting from the stronger confinement of electromagnetic field in two or three dimensions, sensors based on the LSP interrogation of metallic nanostructures are more suitable for sensing of small molecules.^335^ However, their overall refractive index sensitivity is typically less than 400 nm/RIU,^335,336^ which is at least one order of magnitude smaller than that of the SPP-based sensors. Providing great flexibility in the engineering of near- and far-field optical responses, plasmonic metamaterials possess an inherent sensitivity to their local dielectric environment and, therefore, provide a superior platform for biochemical sensing compared to individual nanostructures or smooth metal films. In the past decades, a great number of plasmonic metamaterial-based biochemical sensors have been demonstrated, with considerable attention focused on the improvement of the FOM_λ_ by increasing the RI sensitivity and/or narrowing the fwhm.

#### 7.2.1 Hyperbolic Metamaterial-Based Biochemical Sensors

A variety of plasmonic metamaterials, such as SRR arrays^337,338^ nanohole arrays^339−341^ and nanorod arrays,^123,329,342^ have been exploited for biochemical sensing with excellent RI sensitivity. Among them, hyperbolic metamaterials have attracted significant attention due to the extreme sensitivity of their optical responses to the coupling between meta-atoms, which is influenced by the surrounding environment. Hyperbolic metamaterials have been used to develop biochemical sensors with some of the record performances.^36,129,329,342−347^ For a hyperbolic nanorod metamaterials as an example, the optical response (e.g., extinction and reflection spectra, see details in subsection 2.2) depends not only on the plasmonic response of each nanorod in the assembly but also on the electromagnetic coupling between them. For these reasons, nanorod metamaterials are ideally suited to sensing applications under oblique illumination required to excite the modes with the field along the nanorods.

**Figure 19:**
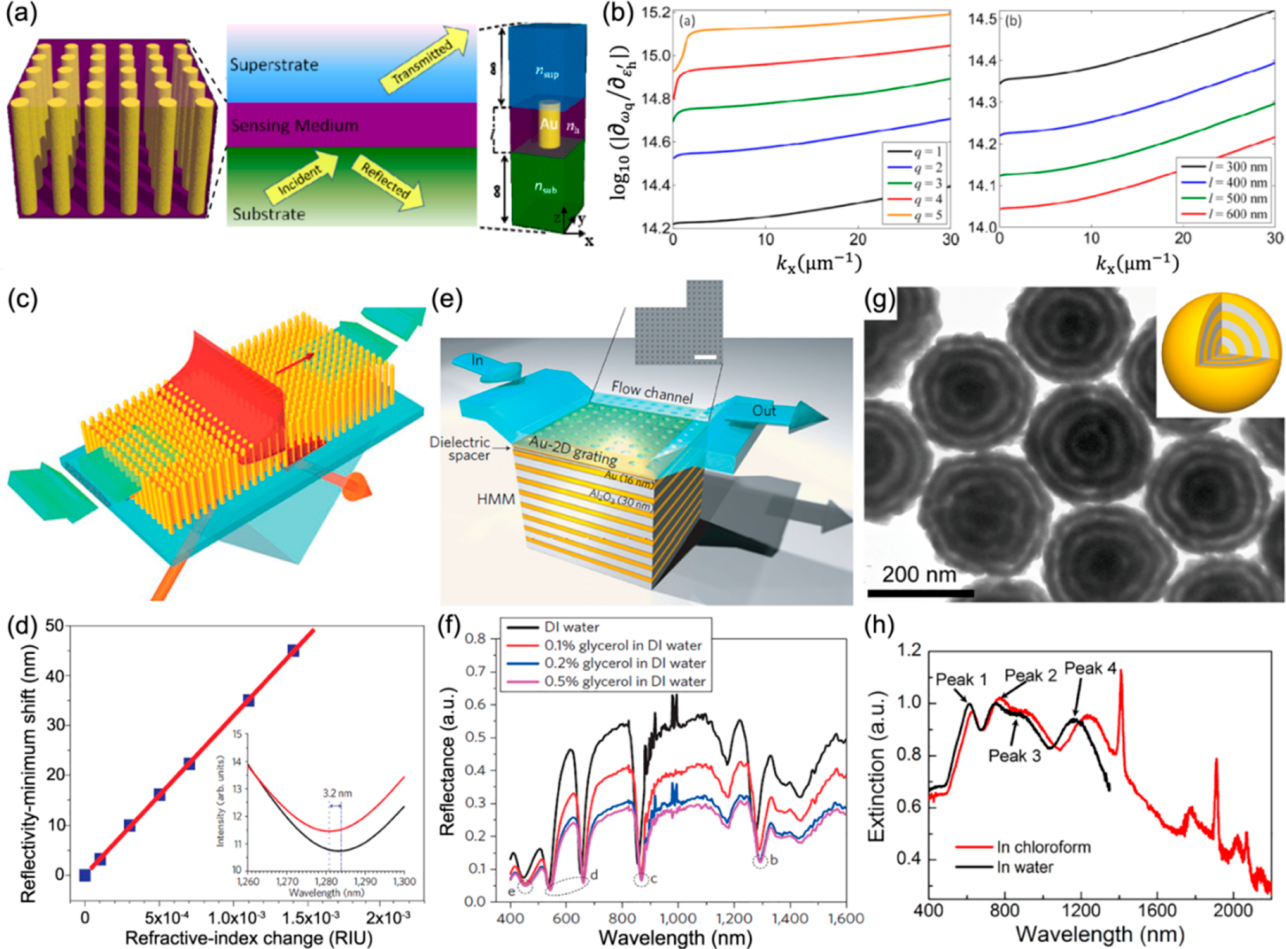
Hyperbolic metamaterial-based biochemical sensors. (a) Schematics of an array of gold nanorods for refractive index sensing in the reflection or transmission geometry. (b) The sensitivity of the metamaterial mode dispersion on the real part of the host medium permittivity ε_*h*_′: (left) the first five modes of the metamaterial layer with thickness *l* = 400 nm and (right) the fundamental mode (*q* = 1) for various thicknesses of the metamaterial. Panels a and b are reproduced with permission from ref (329). Copyright 2015 Optica Publishing Group. (c) Schematic of the ATR measurements in a flow cell configuration. (d) Calibration curve for the metamaterial-based sensor (monitored at the wavelength of 1230 nm) under the steplike changes of the refractive index of the environment. Inset: Reflectivity spectrum modifications with the changes of the refractive index by 10^–4^ RIU. Panels c and d are reproduced with permission from ref (342). Copyright 2009 Springer Nature. (e) Schematic representation of the miniaturized grating-coupled hyperbolic metamaterial sensor with a fluid flow channel. Inset: SEM image of the fabricated 2D subwavelength gold diffraction grating on top of the hyperbolic metamaterial (scale bar, 2 μm). (f) Reflectance spectra of the sensor device obtained by injecting different weight percentage concentrations of glycerol in distilled water. Panels e and f are reproduced with permission from ref (343). Copyright 2016 Springer Nature. (g) TEM image of metaparticles with three pairs of SiO_2_/Au shells. (h) Extinction spectra of the metaparticles dispersed in water (black curve) and chloroform (red curve). Panels g and h are reproduced with permission from ref (348). Copyright 2018 WILEY-VCH Verlag GmbH & Co. KGaA, Weinheim.

The performance of the nanorod metamaterial for sensing applications in various configurations (transmission, reflection, total internal reflection, as shown in Figure 19a) can be evaluated usinga the local EMT model.^329^ The sensitivity of the mode frequency ω_q_ of the *q*-th TM mode to variations of a permittivity of the host medium, *ε*_*h*_, can be derived to be
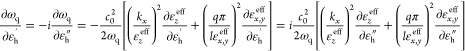
15where *c*_0_ is the speed of light in vacuum, ε_*x*,*y*_^eff^ and ε_*z*_^eff^ are the effective permittivities of the metamaterial for ordinary and extraordinary axes, and *l* is the length of the nanorod in the metamaterial, determining metamaterial thickness. The mode frequency sensitivity with respect to the real part of the host medium permittivity (ε_h_′) increases for higher-order modes of the metamaterial sensor and with a decrease of the metamaterial thickness (Figure 19b). The superior RI sensitivity of higher-order modes is a consequence of their spectral position close to the resonance in ε_*x*,*y*_^eff^ and the increased field gradients inside the metamaterial. These gradients are determined by the mode spatial frequency *qπ*/*l*, increasing with increasing *q* value or decreasing sensor thickness *l*. Notably, the sensitivity to the RI variations of the host medium between the nanorods is at least two orders of magnitude higher than the sensetivity to the RI variations of the superstrate above the metamaterial.

The use of plasmonic hyperbolic metamaterials basd on the nanorod arrays for label-free biochemical sensing was demonstrated with ultra-high sensitivity.^342^Figure 19c shows a typical sensing configuration, in which a gold nanorod metamaterial was attached on the surface of a prism and illuminated in the ATR geometry with a broadband white light. In addition to the resonances observed in transmission, nanorod metamaterials also support waveguided modes, which are largely localized within the metamaterial slab and, therefore, provide an excellent overlap between the sensing field and the sensed substance. As a result, the nanorod metamaterial showed a refractive index sensitivity as high as 32,000 nm/RIU (Figure 19d), which is about 2 orders of magnitude higher than the sensitivity of LSP-based sensors.^335,336^ The FOM_λ_ of the nanorod metamaterial-based sensor reached a value of 330, which is much higher than those of LSP- and SPP-based sensors.^12,334−336^ The detection of biotin molecules with a small molecular weight (244 Da) was further demonstrated by functionalizing the nanorod surface with a streptavidin complex as a receptor. The detection limit of the metamaterial-based sensor to biotin was estimated to be below 300 nM, which is more than two orders of magnitude lower than that of conventional SPP-based sensors using continuous gold films.^12,334^ This approach has recently been further developed by using high-uniformity nanorod hyperbolic metamaterials fabricated by combining EBL and electrodeposition, offering the bulk RI sensitivity of 41,600 nm/RIU and achieving a FOM_λ_ as high as 416.^129^ The improvement in the sensing performance can be attributed to the more regular arrangement of the nanorod in the array fabricated with the EBL approach and the associted narrowing of the resonance.

In addition, nanotube and coaxial rod-in-a-tube metamaterials have also been fabricated and employed for optical sensing.^125,127^ In contrast to the nanorod metamaterials, which require ATR-based or oblique illumination with TM-polarized light, the high-sensitivity plasmonic modes of the nanotube and rod-in-a-tube metamaterials can be excited at normal incidence. However, their refractive index sensitivity is less than 300 nm/RIU, which is much less than that of the waveguided mode in nanorod metamaterials.^12,334−336^

As shown in the above examples, prism-based ATR configurations are usually used to satisfy the momentum-matching condition for the excitation of hyperbolic metamaterial modes. To eliminate the bulky excitation setup, miniaturized grating-coupled sensors based on layered hyperbolic metamaterials were developed.^343,344,349^ The proposed metamaterial sensors consist of a Au/Al_2_O_3_ multilayer hyperbolic metamaterial, a two-dimensional metallic nanohole diffraction grating, and a microfluidic flow channel (Figure 19e). The nanohole grating is placed on the top of the hyperbolic metamaterial to diffract the incident light and produce a wide range of wave vectors for the excitation of high-*k* modes of the metamaterial. Under illumination with TM-polarized light, multiple highly dispersive bulk plasmon modes with high quality factors are observed in the reflectance spectra in the hyperbolic dispersion regime, covering the visible and near-infrared spectral ranges. Both spectral and angular detection schemes have been demonstrated for the grating-coupled hyperbolic metamaterial sensors.^343^ In the former configuration, the layered metamaterial sensor exhibited a maximum sensitivity of 30,000 nm/RIU (Figure 19f), which is similar to that of above-mentioned nanorod metamaterial-based sensors. Benefiting from the narrower line width of the resonance modes, the corresponding FOM_λ_ reaches a record-high value of approximately 590. The high sensitivity of the grating-coupled metamaterials is originated from the extremely sensitive dependence of the coupling condition between grating surface modes and high-*k* modes on the refractive index of the surrounding medium. The ability of the metamaterial platform to detect small-molecular-weight (244 Da) biotins at picomolar concentrations was also reported. When operated in the configuration based on monitoring the angle of resonant excitation of metamaterial modes,^344,349^ which can provide a higher measurement precision owing to its higher signal-to-noise ratio, high angular sensitivities were demonstrated on the order of 7,000°/RIU,^344^ which is about 1 order of magnitude higher than that of existing SPP-based biochemical sensors. This allows the angular detection of large-molecular-weight biomolecules such as Cowpea mosaic virus (5 × 10^6^ Da) at concentrations as low as 1 fM. Hyperbolic metamaterial-based biochemical sensors can be further miniaturized by integrating the metamaterials with optical fibers, such as D-shaped fibers.^350−353^

In addition to substrate-supported metamaterials, a colloidal version of hyperbolic metamaterials, named metaparticles, also provide refractive index sensitivity.^348^ They were realized by coating gold nanospheres with alternating silica and gold layers, forming multishell particles with diameter less than 300 nm (Figure 19g). These metaparticles possess a rich and highly tunable plasmonic mode structure including dipolar and quadrupolar resonances of various orders, covering a broad spectral range from 400 to 2,200 nm (Figure 19h). Compared with gold nanospheres or nanoshells,^335,336^ the metaparticles show greatly improved refractive index sensitivity with a value as high as 740 nm/RIU, which is attractive for optical sensing applications with high spatial resolution. The strong and spectrally broad local-field enhancement in the metaparticles also makes them attractive for applications in surface-enhanced spectroscopies.

#### 7.2.2 Fano Resonant Metamaterial-Based Biochemical Sensors

**Figure 20:**
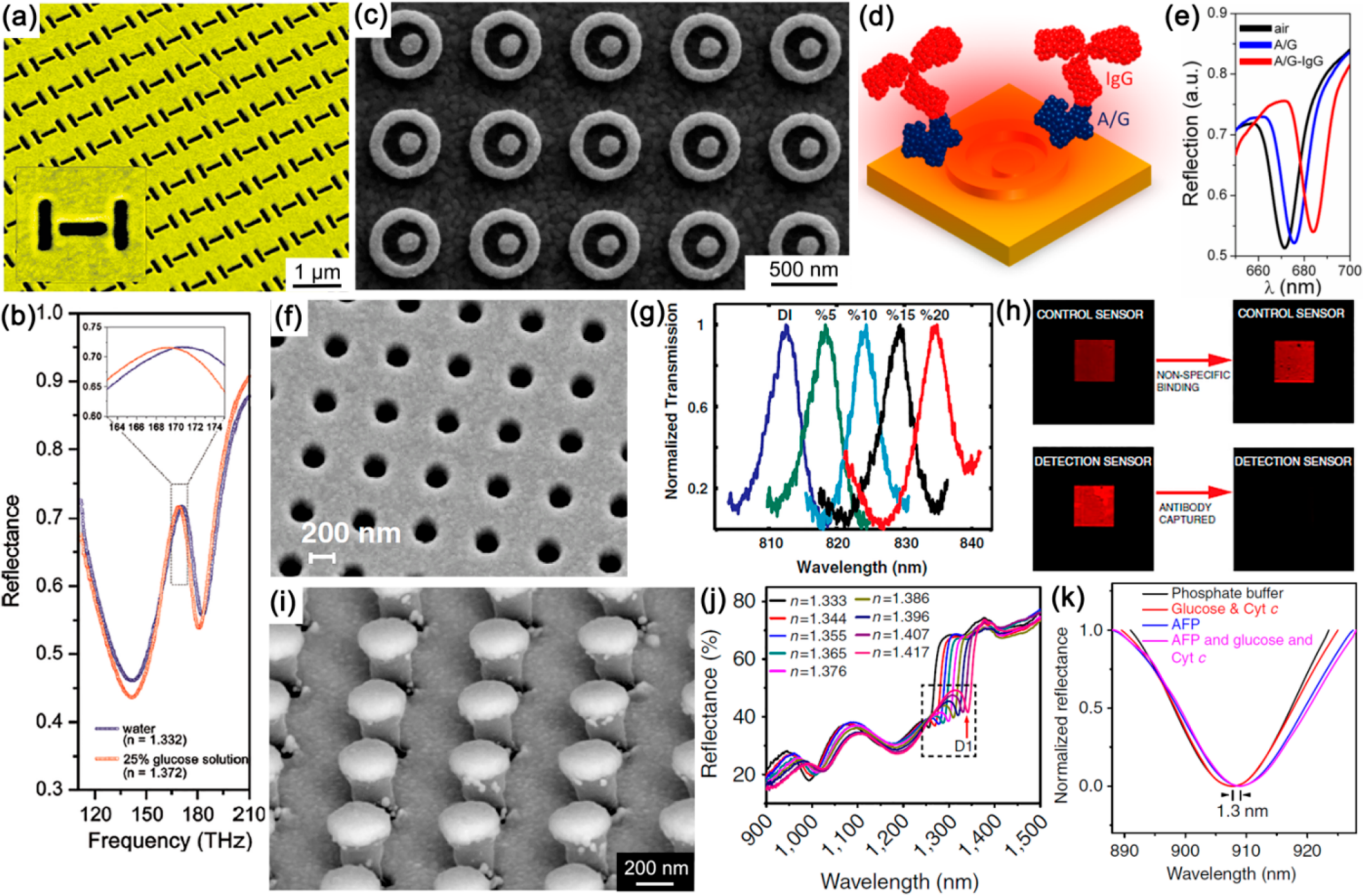
Biochemical sensors based on Fano-resonant metamaterials. (a) SEM image of an array of asymmetric H-shaped cut-out metasurface. Inset: enlarged view of the metamaterial unit cell. (b) The depndence of the reflectance spectrum of the metasurface in panel a on the liquid environment: water and a 25% aqueous glucose solution. Inset: zoom into the reflectance peaks. Panels a and b are reproduced with permission from ref (363). Copyright 2010 American Chemical Society. (c) SEM image of an asymmetric ring/disk cavity array fabricated on a gold ground layer. (d) Schematic illustration of the interaction of the local electromagnetic field of the nanostructures in panel c with proteins and (e) the changes of the reflectance spectra with the introduction of 1 mg/mL protein A/G and 1 mg/mL IgG antibody. Panels c–e are reproduced with permission from ref (365). Copyright 2012 American Chemical Society. (f) SEM image of a plasmonic nanohole array. (g) Resonance shift for the subradiant (+1,0) SPP mode of the nanostructure in panel f with exposed to different NaCl concentrations. (h) Optical images of the transmitted light obtained from detection and control sensors in panel f. A dramatic reduction of the transmitted light intensity through the detection sensor can be observed after the capturing of antibodies. Panels f–h are reproduced with permission from ref (368). Copyright 2011 Proceedings of the National Academy of Sciences of the United States of America. (i) SEM image of a gold mushroom array. (j) Reflectance spectra of a gold mushroom array immersed in glycerine-water mixtures with varying RIs. (k) Reflectance spectra of an anti-AFP-functionalized gold mushroom array when immersed in various analytes for demonstration of selectivity. Panels i–k are reproduced with permission from ref (369). Copyright 2013 Springer Nature.

In addition to the increase of sensitivity, significant efforts have also been made to reduce the fwhm of metamaterial resonances to increase the FOM values. An effective approach to achieve this is to couple a broad plasmonic resonance with a different resonant mode that possesses a smaller fwhm. Fano resonances, which originate from the interference between a broad superradiant mode with a narrow subradiant mode, exhibit asymmetric sharp spectral profiles attractive for optical sensing. In the past decades, Fano resonances have been found not only in individual plasmonic nanostructures (e.g., nonconcentric ring/disk cavities^354−356^ and dolmen-type nanostructures^357^) but also in plasmonic metamaterials based on split-ring arrays^358,359^ and metal nanoparticle oligomers^78,360−362^. By engineering the line shape of Fano resonances to obtain small fwhw and strong field enhancement, the sensing performances of plasmonic metamaterials have been improved.^363−367^ In a complementary planar metamaterial consisting of asymmetric H-shaped cut-out nanostructures (Figure 20a), electromagnetically induced transparency-like Fano resonances have a narrow line width (∼153 nm), which originate from the interference between the spectrally broad bright mode in the slot dipole antenna and the spectrally narrow dark mode supported by the slot quadrupole antenna.^363^ A metamaterial sensor based on this Fano resonance (Figure 20b) exhibits a high RI sensitivity of 588 nm/RIU and a FOM_λ_ of 3.8. In the other realization, by introducing a conducting metal layer underneath an asymmetric ring/disk nanocavity array, Fano resonances in a cavity system with strongly enhanced electromagnetic fields were demonstrated for advanced biochemical sensing.^365^ This cavity system (Figure 20c) supports a Fano resonance with a spectrally sharp feature with the fwhm as small as 9 nm due to the contribution of subradiant and superradiant modes as well as propagating SPP modes residing at the surface of the conducting substrate. At the same time, the electromagnetic fields, supported by the Fano-resonant asymmetric ring/disk system, extend deeply into the surrounding medium (Figure 20d), which greatly enhances the accessibility of the optical fields by analytes. As a result, the RI sensitivity of the Fano resonance in such a metamaterial sensor can be as large as 648 nm/RIU, and the FOM_λ_ reached a value of 72. This enabled the sensitive detection of protein mono-/bilayers. As shown in Figure 20e, the subsequent attachment of protein A/G and IgG antibody resulted in 5 and 14 nm red shifts, respectively, of the spectral feature within the Fano resonance profile.

In addition to the realization of Fano resonances based on coupling of localized modes in each meta-atom, Fano resonances with narrow line widths can also be obtained with SPP modes in plasmonic metamaterials for optical sensing with high performance.^368,369^ With this approach, an ultrasensitive label-free detection technique was realised based on asymmetric Fano resonances in plasmonic nanohole arrays, which originate from the interference between the light transmitted through the holes and the scattered SPP modes supported by the hole arrays (Figure 20f).^368^ By exploiting the subradiant SPP mode (+1,0) with a remarkably small fwhm of ∼4 nm, the experimental refractive index sensitivity was measured to be around 717 nm/RIU along with a FOM_λ_ of 162 (Figure 20g), which is much higher than the theoretically estimated upper FOM_λ_ limit (∼108) of gold-film-based standard SPP sensors under the Kretschmann configuration.^370,371^ The excellent performance of the nanohole array sensor was attributed to the nearly complete suppression of the radiative losses achieved due to the subradiant nature of the the SPP resonance. The direct detection of a single monolayer of biomolecules using these Fano resonances and the associated Wood’s anomalies was demonstrated. As shown in Figure 20h, a dramatic reduction of the transmitted light through a nanohole array, strong enough to be discerned by naked eye, was observed after capturing a monolayer of mouse IgG antibody by the protein A/G deposited on the nanohole array. In another example, high-performance sensing using Fano resonances was demonstrated in an array of submicrometer gold mushrooms.^369^ Each gold mushroom is composed of a gold cap on the top of a photoresist pillar placed in a hole in a gold film (Figure 20i). In addition to localized plasmonic modes supported by the caps and holes, a new coupled plasmon resonance (Fano resonance) with a fwhm as small as 10 nm appeared. It originates from the interference between the Wood’s anomaly (caused by the diffraction of the incident light into a propagating wave) and the scattered light from the gold caps. The Fano resonance shows a high sensitivity to the change of the refractive index of the surrounding solutions (Figure 20j), with a RI sensitivity determined to be around 1015 nm/RIU. Such high sensitivity is mainly due to the greatly increased interaction of the enhanced local fields with molecular species in the gold mushroom array. The combination of the narrow fwhm and high RI sensitivity in such a metamaterial sensor gives rise to a FOM_λ_ as high as 108, which is extremely high in comparison with LSP sensors based on isolated mushroom nanostructures. The same metamaterial was used as a biosensing platform for detecting cytochrome c and alpha-fetoprotein (AFP), with detection limits down to 200 pM and 15 ng/mL, respectively. As an example, Figure 20k shows highly selective detection of AFP with an anti-AFP-functionalized metamaterial; a clear red shift of 1.3 nm was measured for the Fano resonance dip after the exposure of the sensor to only 20 ng/mL AFP with no observable shift for other analytes tested.

### 7.3 Gas Sensing

Plasmonic metamaterials, with their optical response highly sensitive to the dielectric properties of the metamaterial constitutes and the surrounding environment, have also been widely exploited for the detection of gases, such as hydrogen, and carbon monoxide, relative humidity, and volatile organic compounds.^372−374^ Among the various detected gases, hydrogen is important in many areas of chemical industry (e.g., production of ammonia, refinement of crude oil) and is also an important energy source of the future. Nevertheless, hydrogen is colorless, odorless, and highly flammable for a wide range of hydrogen-air mixture concentrations (4–75 vol %) with a very low energy input of ignition, which brings about critical safety concerns due to the risk of explosion. Therefore, fast and sensitive detection of hydrogen at all levels of the hydrogen-based economy is required for the safe use of hydrogen. Conventional hydrogen sensors are mostly based on electric resistance changes and often operated at high temperatures, which increases the explosive hazard. In contrast, plasmonic hydrogen sensors, which not only eliminate the generation of spark in order to minimize the risks of explosion, but also feature remote readout and immunity to electromagnetic interference, are attractive for use in harsh environments.^372^ In the following, we focus on the review of optical hydrogen sensing with plasmonic metamaterials.

Typical plasmonic hydrogen sensors includes hydrogen-active materials, such as transition metals (e.g., palladium, magnesium, yttrium), metal alloys, and metal oxides (e.g., SnO_2_, TiO_2_, WO_3_). Taking palladium as an example, upon exposure to H_2_, palladium is transformed into a metal hydride (less metallic) due to metal–hydrogen interaction, leading to a significant change in its permittivity and volume that can be optically detected. Generally, plasmonic hydrogen sensors can be classified into two types: direct and indirect. For hydrogen sensors with a direct sensing configuration, hydrogen-responsive metals act as both the plasmonic and hydrogen-active materials, reacting with hydrogen, forming hydrides, and finally producing the optical response. Hydrogen sensors with an indirect sensing configuration usually consist of a plasmonic nanostructure fabricated with a hydrogen-inert plasmonic material (e.g., gold, silver) and a hydrogen-active material in its optical near field. Upon exposion to hydrogen gas, the hydrogen-active material undergoes a chemical transformation, resulting in a change of its RI and volume expansion, which is then probed through the optical response of the plasmonic nanostructure.

**Figure 21:**
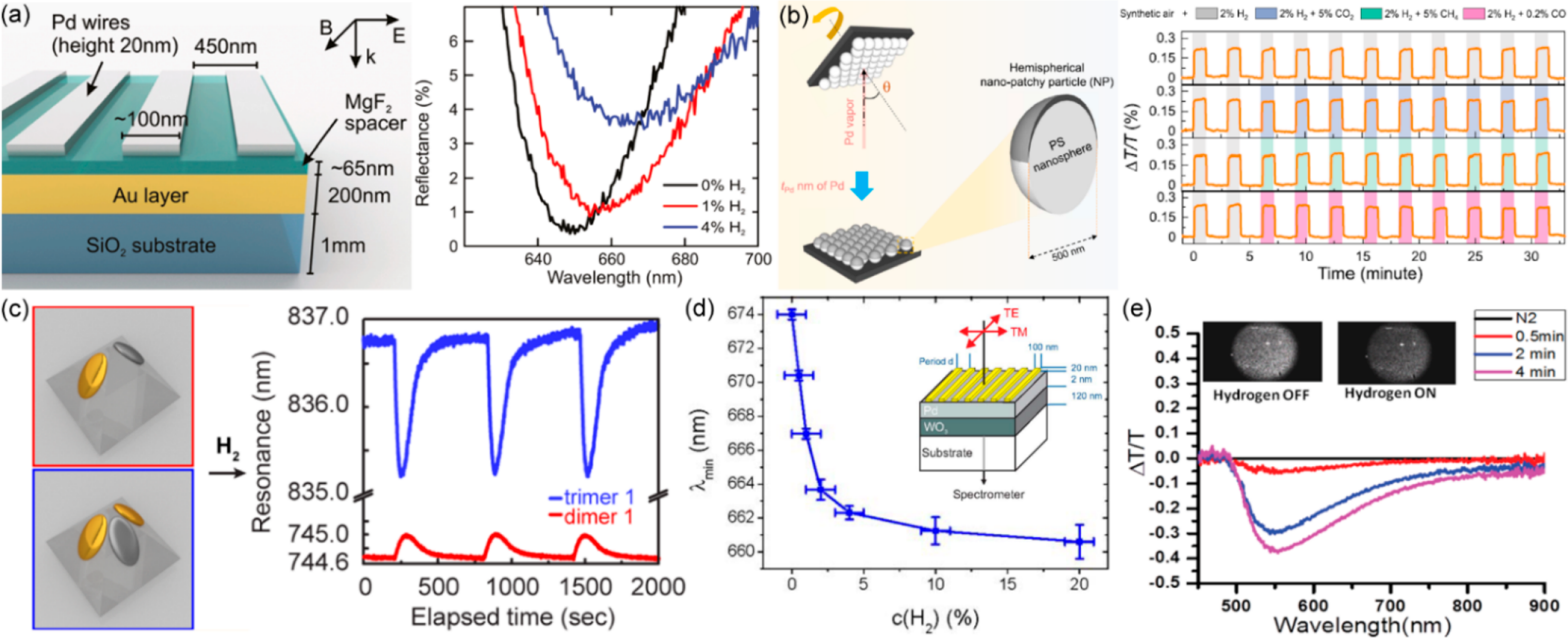
Plasmonic hydrogen sensors. (a) Schematics of the plasmonic perfect absorber based hydrogen sensor with palladium nanowires stacked above a gold mirror separated by a MgF_2_ film, and reflectance of the sensor for different hydrogen concentrations. Reproduced with permission from ref (375). Copyright 2011 American Chemical Society. (b) Schematics of the fabrication procedure and nanoarchitecture of an optical hydrogen sensor based on a hexagonal array of palladium hemispherical nanoparticles, and the time-resolved Δ*T*/*T* response of a composite Pd_80_Co_20_ nanoparticle/PMMA sensor to different H_2_ gas mixtures. Reproduced with permission from ref (376). Copyright 2021 Springer Nature. (c) Time dependence of the optical responce of Au–Pd dimer (red) and trimer (blue) arrays upon injection of H_2_ and N_2_ gases (three cycles). Reproduced with permission from ref (372). Copyright 2014 American Chemical Society. (d) Spectral shift of the extinction minimum with varying concentrations of H_2_ for the metallic photonic crystal slab using WO_3_ as the waveguide layer material shown in the insert. Reproduced with permission from ref (377). Copyright 2010 Optica Publishing Group. (e) Variation of transmission (angle of incidence is ∼40°) of a Au–Pd core–shell nanorod metamaterial on exposure to 2% hydrogen gas in nitrogen. Inset: photographs of the sensor in the absence and presence of hydrogen gas. Reproduced with permission from ref (123). Copyright 2014 WILEY-VCH Verlag GmbH & Co. KGaA, Weinheim.

A variety of plasmonic metamaterial-based direct hydrogen sensors have been developed, including palladium nanohole arrays,^339,378^ palladium nanowire arrays,^379,380^ palladium nanohelix arrays,^381^ and metal–insulator–metal nanostructure-based perfect absorbers.^375,382−384^ For example, in a palladium-based perfect absorber structure,^375^ an array of palladium nanowires stacked above a MgF_2_ spacer layer separating it from a gold bottom mirror (Figure 21a) was used to ensure nearly zero transmission through the structure due to the strong coupling between the plasmon resonances in the nanowires and the image dipoles induced in the gold mirror. Upon exposure of the sensor to 1% and 4% H_2_ in N_2_ carrier gas, the sensor showed an obvious change in the reflectance (Figure 21a). The maximum change in the reflectance value of ∼4.4% at 650 nm wavelength and a spectral red shift of the resonance of 19 nm were observed when the H_2_ concentration changes from 0% to 4%. The response time of the sensor is in the range of 10–50 s. However, for pure palladium-based hydrogen sensors, problems including hysteretic behavior, long response time, and sensor poisoning by trace amounts of species such as CO and NO_2_ remain widely unresolved. Recently, these long-standing limitations can be overcome by the use of palladium-alloy nanostructures covered with a tailored thin polymer layer.^376,385,386^ In these realizations, a compact optical hydrogen sensing platform with a subsecond response time, sub-10-ppm LOD, and excellent robustness against interfering gases was realized.^376^ The sensor is composed of a palladium-alloy nanopatch array (fabricated by glancing angle metal deposition on a hexagonally packed polystyrene nanosphere monolayer (Figure 21b) and features a simple transmission intensity detection method. By alloying palladium with 20% Co, the sensing performance of the Pd_80_Co_20_ sensor was significantly enhanced compared with its pure palladium counterpart, showing a response time of just 0.85 s for 1 to 100 mbar of H_2_ partial pressure, a LOD of as low as 2.5 ppm, and an excellent accuracy (<2.5%). Moreover, upon coating with a thin layer of PMMA, the Pd_80_Co_20_ sensor exhibited an excellent robustness against interfering gases such as CO_2_, CH_4_, or CO (Figure 21b), temperature, relative humidity, and aging, which is of great importance for practical applications. It is worth noting that magnesium-based plasmonic metamaterials, whose optical response can be dynamically modulated upon exposure to hydrogen, as discussed in subsection 6.2, can also be developed for hydrogen sensing applications.

Hydrogen-responsive metals, such as palladium, are rather “poor” (highly damped) plasmonic materials, which may limit the sensing performance due to their very broad plasmonic resonances . It is possible to sense hydrogen using nanostructures with superior plasmonic properties as sensors to probe the hydrogen-active entities located in their optical near fields. One approach for such indirect hydrogen sensing is to place a hydrogen-active metal nanostructure in the close vicinity of a plasmonic antenna, which has been successfully demonstrated in the case of both single heteronanostructures^387−389^ and heteronanostructured arrays (Figure 21c).^372,390−395^

Other types of sensors can be developed by attaching plasmonic nanostructures to a film of a hydrogen-active material.^377,396,397^ A gold nanowire array fabricated on top of a gasochromic WO_3_ planar waveguide (an ultrathin 2 nm catalytic palladium layer was incorporated between the nanowire array and the waveguide) undergoes a strong modification of its optical properties when exposed to H_2_.^377^ The plasmon resonance of the nanowires can couple to the quasi-guided mode of the WO_3_ waveguide, resulting in the formation of a polariton-type coupled system with a sharp extinction dip between two pronounced maxima with a small fwhm. A resonance blue shift as large as 13 nm was observed when the concentration of hydrogen increases from 0% to 20% (Figure 21d), along with the extinction change up to 247%.

A similar approach is based on core–shell nanostructures, which provide much higher surface area compared to the thin-film realizations discussed above.^123,398−400^ In this way, the sensing capability of hyperbolic nanorod metamaterials discussed in subsection 7.2.1 can be extended to the detection of gases by coating a thin layer of palladium on the surface of the nanorods.^123^ A ∼40% change in the transmission of the Au–Pd core–shell nanorod array was demonstrated when exposed to 2% H_2_ (Figure 21e). This is clearly noticeable to the naked eye as a change in the intensity of light transmitted through the metamaterial (inset of Figure 21e). The high sensitivity of this sensor to hydrogen results from a combination of both the change of the plasmonic properties of individual core–shell nanorods and the modification of inter-rod coupling in the metamaterial due to the refractive index and thickness changes of the palladium shell, both affecting the optical properties of the metamaterial. The sensor can be rapidly reset (<30 s) by heating under illumination with laser light.

### 7.4 Surface-Enhanced Spectroscopy

#### 7.4.1 Surface-Enhanced Raman Scattering

Raman scattering is an inelastic scattering process of photons by molecules, which results in a shift of the frequency of the incident photons due to the excitation of molecules into higher vibrational or rotational energy states. Therefore, different from the detection of molecules based on the change of the local dielectric environment, Raman spectroscopy provides a spectral fingerprint of molecules and has been widely used for material identification and analysis. To overcome the small cross section of Raman scattering, surface-enhanced Raman scattering (SERS) based on local field enhancement from surface plasmons has been developed to amplify Raman signals by many orders of magnitude,^401−405^ which allowed to achieve sensitivity down to the single-molecule level. However, one of the major concerns in SERS-based biochemical sensing is the problem of poor uniformity, reproducibility, and the related quantification of the SERS signals with conventional SERS substrates (e.g., aggregated nanoparticles) since the SERS enhancement may depend on the smallest variations of the geometry and feature sizes. Benefiting from advantages provided by engineering of the spectral response and local field enhancement, plasmonic metamaterials provide an attractive platform as substrates for SERS spectroscopy with pronounced signal enhancement and good signal uniformity and reproducibility. In this section, we review the application of plasmonic metamaterials in SERS for molecular detection and analysis.

**Figure 22:**
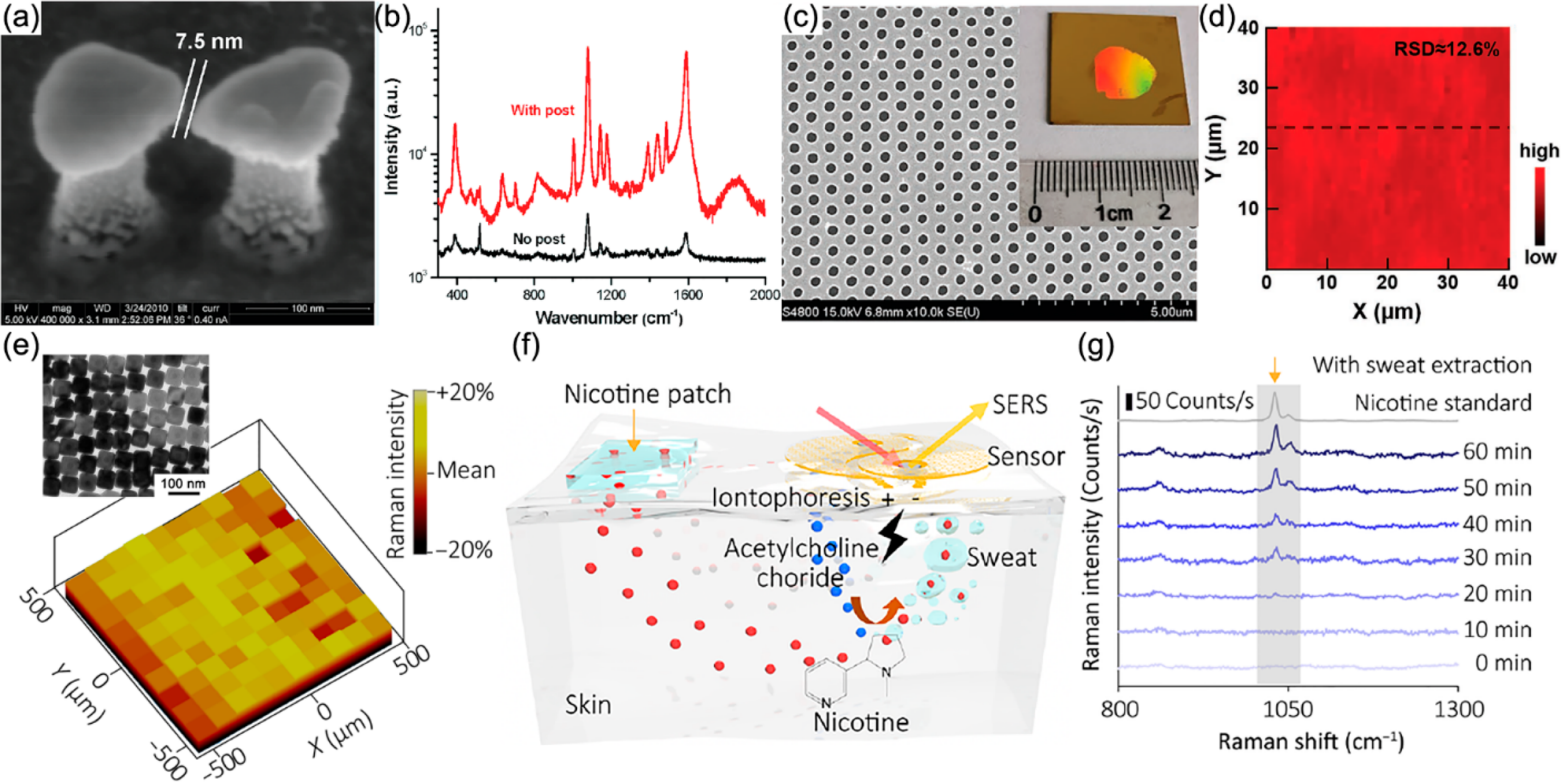
Application of plasmonic metamaterials in SERS for molecular detection and analysis. (a) SEM image of a three-dimensional gold bow tie nanoantenna with a gap of 8 ± 1 nm. (b) Comparison of SERS spectra of *p*-mercaptoaniline from elevated and nonelevated bow tie array substrates. Panels a and b are reproduced with permission from ref (406). Copyright 2010 American Chemical Society. (c) SEM image of a plasmonic metamaterial formed by a gold nanohole array/SiO_2_ spacer/gold film structure. The inset shows a photo of the metamaterial sample. (d) SERS image of the nanostructure obtained using the intensity of the 1580 cm^–1^ peak of benzenethiol. Panels c and d are reproduced with permission from ref (407). Copyright 2021 Wiley-VCH GmbH. (e) SERS intensity map (the wavelength of ∼1621 cm^–1^) of the metasurface after the treatment with the Raman probe (crystal violet, 10^–5^ M). Inset: TEM image of a plasmonic metasurface formed by an ordered silver nanocube array. (f) Schematic illustration of the working principle of the sweat extraction system and (g) real-time monitoring of nicotine in human skin using the integrated sensor with sweat extraction. Panels e–g are reproduced with permission from ref (408). Copyright 2021 American Association for the Advancement of Science.

In the past decades, various types of metamaterial-based SERS substrates have been demonstrated. For example, SRR metamaterials offer two transducing channels for parallel acquisition of quantitative binding data and characteristic fingerprints of biomolecules, enabled by the simultaneous probe of optical transmission and sensitive SERS spectra.^409,410^ Self-assembled plasmonic metamaterials at liquid/liquid or liquid/air interfaces provide the ability of trace SERS-based detection of multianalytes from the aqueous, organic, or air phase.^411−413^ Within this approach, great efforts have been dedicated to increasing the enhancement factor through the field enhancement in order to lower the limits of the detection.^120,406,414−416^ Using an array of silver nanorods, strong dependence of the SERS signal on the metamaterial geometry was demonstrated, showing an over 200-fold SERS intensity increase by varying the inter-rod gap distance from 35 to 10 nm.^414^ This is explained by the increase of the electromagnetic fields in the gaps between the neighboring nanorods with the decreased inter-rod gap distance. Employing elevated gold bowtie nanoantenna arrays with a gap thickness of ∼7.5 nm (Figure 22a), large SERS enhancement factors were obtained exceeding 10^11^, which was attributed, on one hand, to the greatly enhanced local fields in the nanoantenna gap and, on the other hand, to the elevated structure that can produce up to 2 orders of magnitude additional enhancement in the SERS response due to its radiation efficiency (Figure 22b).^406^ Generally, SERS signals dramatically increase with the decrease of the distance between plasmonic components due to the increase of the local field, but this tendency is reversed at the distances smaller than ∼1 nm, at which the local field enhancemnet is detriorated by the onset of the electron tunnelling.^417^

In contrast to conventional SERS substrates , metamaterial-based substrates have good controllability and reproducibility in fabrication, providing uniform and reproducible SERS signals over a large area highly required for practical applications.^407,408,418^ Using holographic lithography, plasmonic metamaterials formed by gold nanohole array/SiO_2_ spacer/gold film structures (Figure 22c) were fabricated for SERS detection with high uniformity.^407^ The size of the metamaterial sensing area can be as large as 10 mm (inset of Figure 22c), and the SERS mapping of the metamaterial on random areas of 40 × 40 μm^2^ shows an excellent SERS signal uniformity (Figure 22d). The calculated relative standard deviation of the measured signal was about 12.6%, which meets the requirement for a commercial SERS substrate.^419^ Using an ordered silver nanocube superlattice metafilm (inset of Figure 22e) fabricated via a Langmuir–Blodgett self-assembly approach on a flexible polymer substrate, the relative standard deviation of the SERS signal was demonstrated to be only ∼3.9% (Figure 22e), suggesting an excellent signal reproducibility and high potential for quantitative analysis.^408^ This plasmonic metamaterial, integrated with a flexible electronic system capable of automatically extracting sweat and analytes from the body (Figure 22f), was a basis for a wearable sensing platform with an almost “universal” molecular recognition ability. Figure 22g shows an example of the real-time monitoring of nicotine in human skin with this integrated wearable sensor.

#### 7.4.2 Surface-Enhanced Infrared Absorption

In addition to Raman scattering-based spectroscopy, infrared (IR) absorption spectroscopy is an alternative technique for the chemical identification of molecules through their vibrational fingerprints. Surface-enhanced infrared absorption (SEIRA), which can significantly increase the IR absorption of molecules via either surface plasmon-enhanced light-molecule interactions or molecular dipole enhancement, has attracted significant interest. It was first demonstrated with thin metal films covered by molecular monolayers using the ATR configuration.^421^ Subsequently, SEIRA was widely investigated employing various roughened metal surfaces and metal-island films.^422−426^ Since the plasmonic response of these structures usually lies in the visible spectral range, the mechanism of the observed SEIRA effects can be explained by the excitation of vibrations of the absorbed molecules through the induced fields in the metal islands.^423^ Differently, by engineering the geometry and material composition of meta-atoms as well as their near-field coupling, the optical response of plasmonic metamaterials can be readily tuned into the near- and mid-IR ranges to achieve strong field confinement and enhancement. Therefore, plasmonic metamaterials provide an ideal platform for SEIRA spectroscopy with high detection sensitivity.

**Figure 23:**
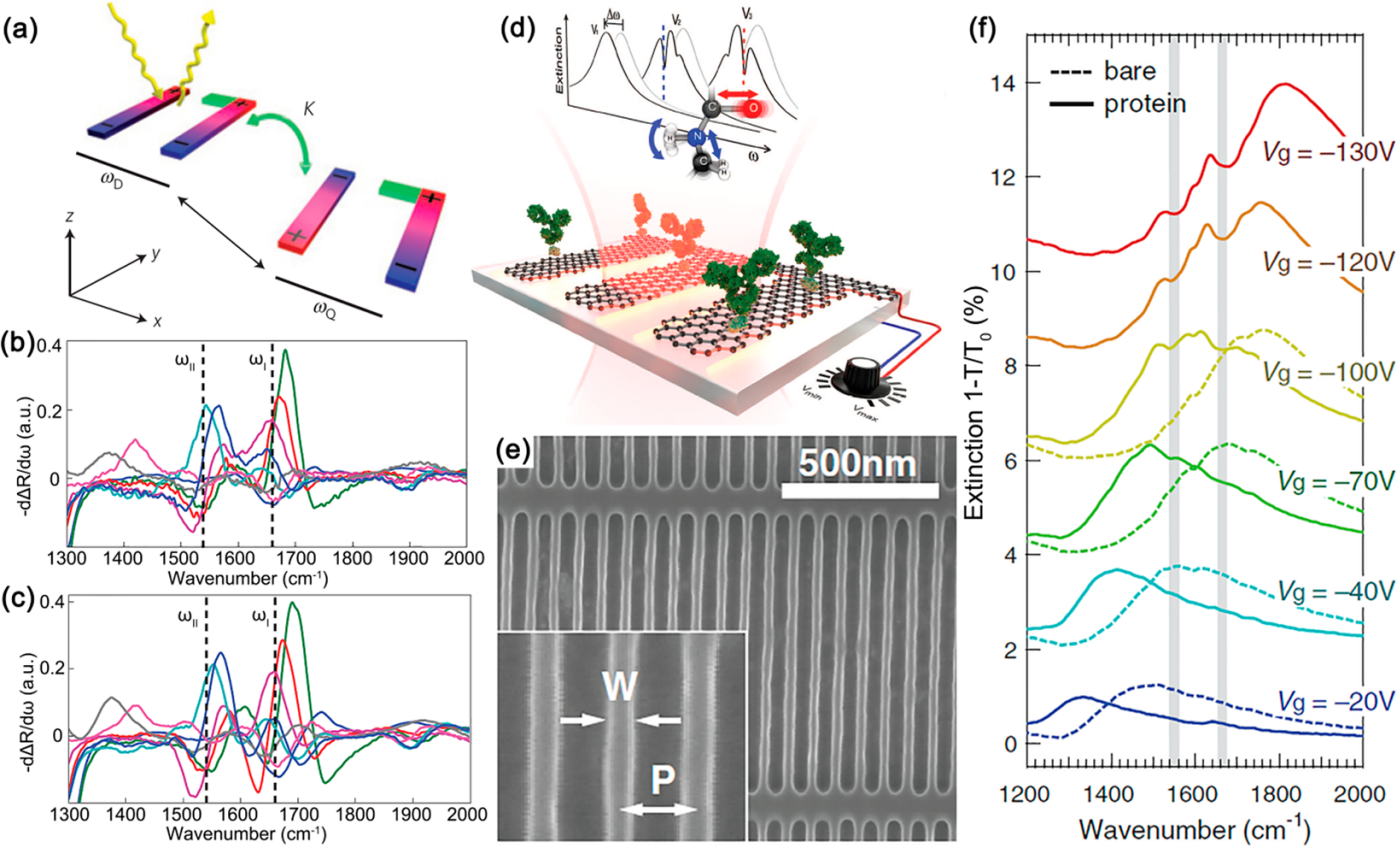
Application of plasmonic metamaterials for SEIRA spectroscopy. (a) Schematic charge distributions for subradiant (Q) and superradiant (D) modes of a Fano-resonant asymmetric metamaterial excited by incident light. Sensing data for (b) the protein A/G monolayer and (c) the protein A/G + IgG antibody bilayer from different asymmetric metamaterial-based pixels. Vertical dashed lines: frequencies of protein backbone vibrations (amide I/II) strongly coupled to the metamaterial modes. Panels a–c are reproduced with permission from ref (366). Copyright 2012 Springer Nature. (d) Conceptual view of a tunable graphene mid-infrared biosensor. (e) SEM image of a graphene nanoribbon array with a width and a period of 30 and 80 nm, respectively. Vertical nanoribbons are electrically interconnected by horizontal strips to maintain the graphene surface at a uniform potential. (f) Extinction spectra of the graphene nanoribbon array for various bias voltage V_g_ before (dashed curves) and after (solid curves) protein bilayer formation. Extinction was calculated as the relative difference in transmission between regions with (T) and without (T_0_) graphene nanoribbons. Gray vertical strips indicate the amide I and II vibrational bands of the protein. Panels d–f are reproduced with permission from ref (420). Copyright 2015 The American Association for the Advancement of Science.

A variety of metal-based plasmonic metamaterials (e.g., metallic hole arrays,^427^ SRRs,^428,429^ nanoantenna arrays^430−435^) have been exploited for SEIRA applications with strong signal enhancement and high reproducibility. Significant efforts have been focused on the development of SEIRA substrates providing intense local fields and, therefore, high detection sensitivity.^366,427,428,435,436^ Employing an SRR array which has large near-field enhancement in the gap, IR detection of molecules with zeptomole-level sensitivity was demonstrated enabled by the resonant coupling of plasmonic modes of the SRR array and IR vibrational modes of the molecules.^428^ By using a gold cross nanoantenna array elevated over a gold layer, a monolayer of biomolecules was successfully detected with a SEIRA signal about four times higher than that from a control nonresonant metamaterial.^435^ The improved detection sensitivity is due to the access to extremely enhanced electromagnetic fields formed between each gold cross nanoantenna and the gold ground layer. Despite the important requirement of selective detection of multiband molecular vibrational modes over a broad IR range, the operating frequencies of plasmonic metamaterials for SEIRA are usually fixed after the fabrication. By integrating metamaterials with a stretchable substrate (e.g., polydimethylsiloxane), the resonant frequency can be precisely tuned in a wide spectral range, by applying mechanical force, to cover many different vibrational modes of the analyte.^429^ Simultaneous detection of multiple spectral fingerprints of different moieties with SEIRA, which can provide a better accuracy for the identification of analytes, has also been investigated. Detection of two molecular vibrational modes of PMMA using just a 4 nm thick PMMA layer was achieved with a dual-band perfect absorber based on a gold nanocross metamaterial.^430^ In another example, employing a Fano-resonant asymmetric metamaterial exhibiting sharp resonances caused by the interference between subradiant and superradiant plasmonic resonances (Figure 23a), quantitative biosensing and fingerprinting of nanometer-scale multimolecular nanoassemblies was demonstrated.^366^ Vibrational fingerprints (e.g., the amide I and II of proteins) of the protein A/G monolayer (Figure 23b) and the protein A/G and IgG antibody bilayer (Figure 23c) were clearly detected with high signal enhancement. Additionally, simultaneous detection of multiple spectral fingerprints of different moieties with SEIRA can be realized using plasmonic metamaterials with broadband resonance in the mid-IR. By self-assembling plasmonic nanoshells resonant in the near-infrared in a two-dimensional periodic array with sub-10-nm interparticle gaps, the simultaneous enhancement of Raman scattering and infrared absorption was achieved.^437,438^ In these close-packed nanoshell arrays, the multipolar plasmon resonances of individual nanoshells hybridize forming a relatively narrow visible or near-infrared resonance originating from the coupled quadrupolar nanoshell resonances, which provides a SERS enhancement factor on the order of 10^8^–10^9^, and a broad mid-infrared resonance (∼2–8 μm) arising from the coupled dipolar resonances, which enhances SEIRA by a factor on the order of 10^4^. By integrating infrared plasmonic metamaterials with a microfluidic chamber, real-time and in situ SEIRA characterization of biomolecules in aqueous solutions can be further realized,^431,433^ which is of great interest for practical applications.

Recently, graphene has emerged as an attractive material for mid-IR plasmonics,^439−441^ exhibiting unprecedented optical confinement and active tunability of the spectral response (by varying the doping level, for example, using electrical gating) that are interesting for mid-IR plasmon-enhanced infrared spectroscopy.^420,442−446^ In this way, high-sensitivity detection of refractive index and vibrational fingerprints of biomaterials was realized with graphene metamaterials.^420^ As schematically illustrated in Figure 23d,e, upon infrared illumination, the plasmon is excited across the nanoribbons, which greatly enhances optical interaction with protein molecules adsorbed on graphene. Protein sensing is achieved by electrostatically-tuned spectral sweeping the plasmonic resonance across the molecular vibration bands and detecting the associated narrow absorption lines. Experimentally, graphene plasmons in a nanoribbon array demonstrated a superior sensitivity in the detection of a protein bilayer both in transmission and SEIRA spectroscopies (Figure 23f). A red shift of the plasmon resonance frequency exceeding 200 cm^–1^ was observed. Furthermore, two spectral dips, which coincide with the amide I and II bands of the protein, emerged and became progressively more intense with the increase of the spectral overlap (e.g., for *V*_*g*_ = −130 V). Due to the extremely strong field confinement of graphene plasmons in the mid-IR range, the graphene nanoribbon sensor provides an obvious increase in both spectral shift (∼6 times) and SEIRA signal (∼3 times) when compared with those of a gold plasmonic sensor. In addition to graphene, plasmonic metamaterials with optical response in the infrared range based on doped semiconductor materials (e.g., Si,^447^ Ge,^448^ indium tin oxide,^449^ InAs^450^) can also be used for SEIRA spectroscopy.

### 7.5 Chiral Sensing

Chirality, a property related to broken mirror symmetry, is a ubiquitous phenomenon in nature and plays an important role in life. It is well-known that the chemical and physical properties of chiral natural biomolecules (as well as synthesized chiral molecules in drugs) are strongly influenced by their chirality.^451,452^ Therefore, discrimination of enantiomers is of extreme importance in biology and pharmaceutical industry. Chiroptical spectroscopy, which measures CD and optical rotatory dispersion (ORD), is a powerful tool for the detection of chiral molecules of different kinds and has been widely used in biology and chemistry to analyze the secondary structure and conformation of biomolecules. However, the chiral optical response of biomolecules mostly lies in the ultraviolet spectral region from ∼150 to 250 nm and is inherently weak, making it difficult to detect with high precision. Therefore, high concentrations or large volumes of analytes are usually required to study many of the aforementioned properties. Plasmonic metamaterials, which have the ability to enhance chiroptical signals in their local environment around the plasmon resonance frequency, usually extended in the visible spectral range,^453^ provide new possibilities for chiral sensing of biomolecules with high sensitivity. In this section, we review the recent advances in chiral sensing using plasmonic metamaterials.

**Figure 24:**
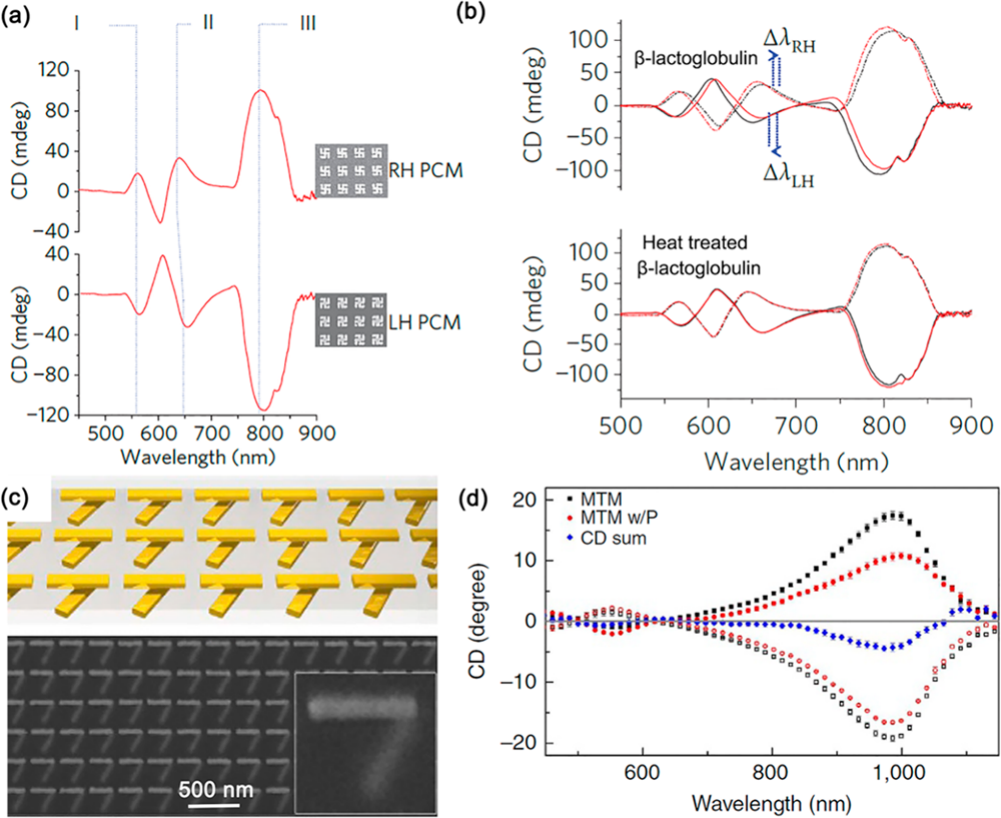
Application of plasmonic metamaterials in chiral sensing. (a) CD spectra of left- and right-handed gold gammadion arrays immersed in distilled water. SEM images of the corresponding chiral metamaterials are in the inserts. (b) Influence of the absorbed proteins β-lactoglobulin and thermally denatured β-lactoglobulin on the CD spectra of the chiral metamaterials. Red spectra were collected in Tris buffer before protein adsorption (solid line, left-hand chiral metamaterial; dashed line, right-handed metamaterial), and black spectra were collected after protein adsorption. Panels a and b are reproduced with permission from ref (454). Copyright 2010 Springer Nature. (c) SEM image and illustration of a +60° twisted metamaterial. (d) CD spectra of the metamaterial in panel c functionalized with a monolayer of protein (Concanavalin A) for ±60° twisted metamaterials (MTM): (solid symbols) +60° metamaterials and (empty sympols) −60° metamaterials. Panels c and d are reproduced with permission from ref (455). Copyright 2017 Springer Nature.

Chiral metamaterials (section 2.3) can create a strong optical chirality in the near field. Therefore, they provide an attractive platform for chiral sensing of biomolecules.^454−462^ Superchiral electromagnetic fields, generated by the optical excitation of plasmonic planar chiral metamaterials, are highly sensitive probes of chirality of supermolecular structures.^454^ Planar chiral metamaterials used in this experiment were composed of left- and right-handed gold gammadion arrays fabricated on a glass substrate (inset of Figure 24a), with their CD spectra being essentially mirror images of each other (Figure 24a). Three distinct resonance modes (labeled I, II, and III) in the CD spectra can be attributed to the excitation of LSPs in the metamaterials. Upon the adsorption of a monolayer of chiral molecules (β-lactoglobulin, which has high levels of β-sheet secondary structure) onto the surface of the left- and right-handed gold gammadion arrays, the strong coupling between the chiral-shaped gammadions and the chiral molecules resulted in dissymmetrical shift of the metamaterial resonances δΔλ = Δ*λ*_*RH*_ – Δ*λ*_*LH*_ ≈ 16 nm, where Δ*λ*_*LH*_ and Δ*λ*_*RH*_ are the wavelength shifts of the LSP modes for left- and right-handed metamaterials (Figure 24b). In contrast, in the case of heat treated β-lactoglobulin, which lost the β-sheet secondary structure, a markedly smaller dissymmetry (δΔλ ≈ 0) was observed (Figure 24c). The difference in the effective refractive indices of chiral samples (∼10^–2^–10^–1^) of left- and right-handed metamaterials for superchiral fields of opposite handedness was estimated to be up to 10^6^ times greater than those observed in optical polarimetry measurements (∼10^–7^), thus allowing picogram quantities of absorbed molecules to be characterized. Using this approach, proteins with different contents of β-sheets can be distinguished. Later studies have demonstrated that chiral plasmonic metamaterials, such as Shuriken metamaterials, enable the detection of higher-order (tertiary/quaternary) hierarchical structure of proteins at the pictogram level,^457^ proteins with similar structure but having primary sequences that differ by a single amino,^459^ and structural order of proteins in complex biointerfaces.^460^

Using left- and right-handed twisted optical metamaterials (Figure 24c), ultrasensitive (zeptomole scale) detection of chiral molecules was demonstrated without relying on any spectral shift.^455^ This was realized by summing the measurements obtained with the enantiomeric pair of metamaterial substrates functionalized with the same analytes to remove the large background CD signals from the metamaterial and cancel its effective contribution to the output CD response. Figure 24d shows CD measurements of a monolayer of Concanavalin A proteins spin-coated on ±60° twisted optical metamaterials. The resultant ΣCD (blue diamonds) clearly show a pronounced dip to the negative values near a wavelength of 970 nm, indicating the left-handed nature of the proteins. The concentration of the detected molecules in the imaging area was as low as ∼55 zeptomoles (corresponding to ∼44 molecules per unit cell of the metamaterial), which is 10^15^ times less than what typical commercial CD spectroscopy tools are able to detect.

## 8 Molecular Plasmonics with Metamaterials for Nanochemistry

**Figure 25:**
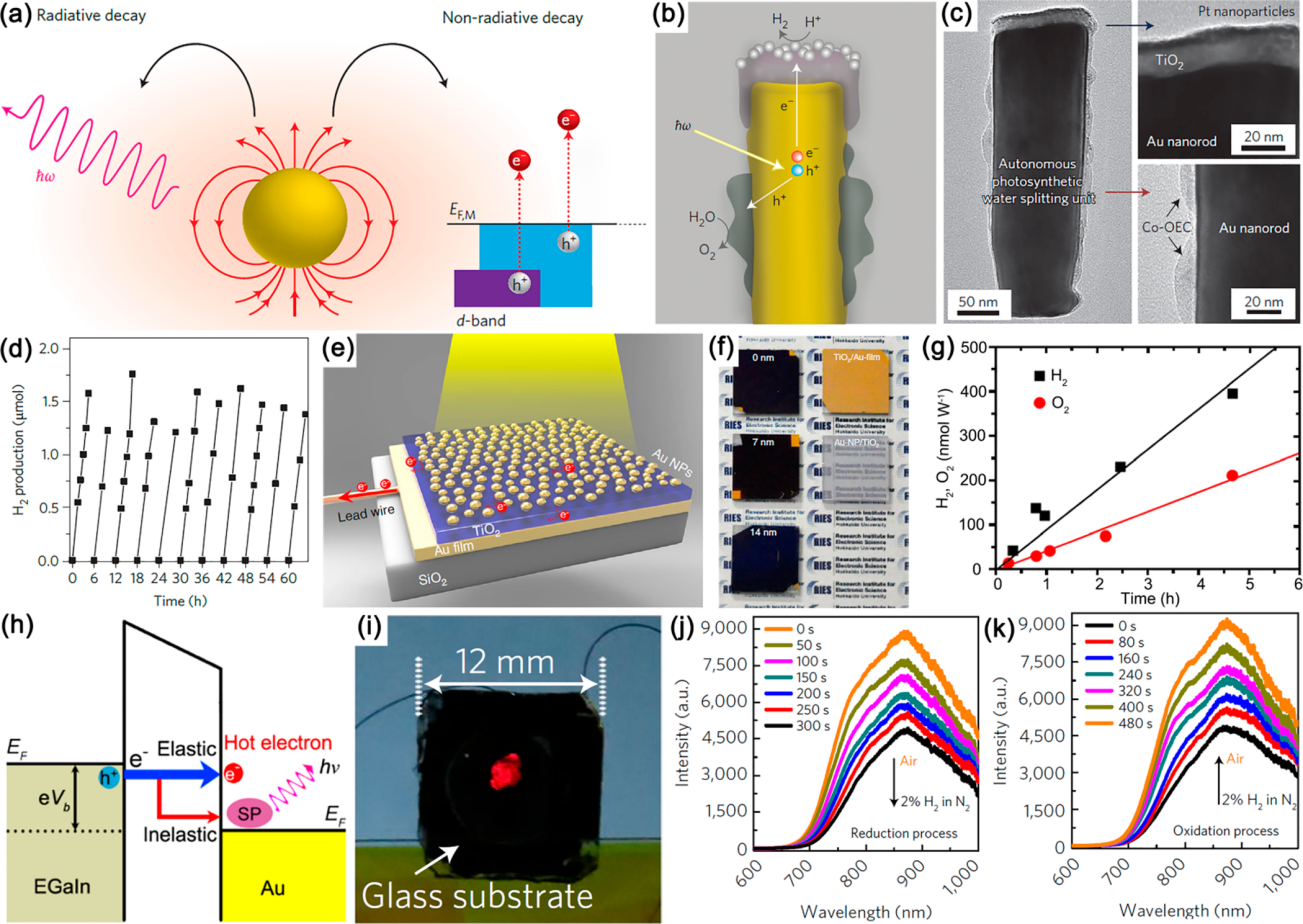
Exploitation of hot carrier-molecule interactions in plasmonic metamaterials for nanochemistry. (a) Schematics illustrating LSP decay channels: radiative via re-emission of photons or nonradiative via excitation of hot electrons. Reproduced with permission from ref (463). Copyright 2014 Springer Nature. (b) Schematic of a meta-atom of the nanorod metamaterial, acting as an individual photosynthetic unit and (c) corresponding TEM image (left) and magnified views of the platinum/TiO_2_ cap (top right) and the Co-OEC (bottom right). (d) Hydrogen evolution under visible-light illumination (with wavelength larger than 410 nm)^2^. Panels b–d are reproduced with permission from ref (465). Copyright 2013 Springer Nature. (e) Schematic of the gold nanoparticle/TiO_2_/gold film with partially inlaid gold nanoparticles. (f) Photographs of gold nanoparticle/TiO_2_/gold film structures with inlaid depths of 0, 7, and 14 nm (left panel). Photographs of the 28 nm-TiO_2_/gold film nanostructure without gold nanoparticles and the gold nanoparticle/28 nm-TiO_2_ structure without the gold film are shown in the right panel for comparison. (g) H_2_ and O_2_ evolution on the Pt cathode and gold nanoparticle/TiO_2_/gold film photoanode with 7 nm inlaid gold nanoparticles under visible-light illumination. Panels e–g are reproduced with permission from ref (466). Copyright 2018 Springer Nature. (h) Inelastic and elastic tunneling of electrons in plasmonic tunnel junctions for the excitation of plasmons and hot electrons, respectively. (i) Photograph of the emission from an electrically driven nanorod metamaterial under a bias of 2.5 V. (j and k) Evolution of the emission spectra of an electrical driven metamaterial under a bias of 2.5 V when the cell atmosphere was switched (j) from air to 2% H_2_ and (k) from 2% H_2_ to air. Panels h–k are reproduced with permission from ref (119). Copyright 2018 Springer Nature.

Following the excitation in metal nanostructures upon light absorption, surface plasmons can decay either radiatively through re-emission of photons or nonradiatively through the generation of hot electron–hole pairs via Landau damping on a femtosecond time scale (Figure 25a).^463^ The generated hot carriers relax through electron-electron and electron-photon scattering processes and ultimately result in the heating of the nanostructures.^49^ The nonradiative decay channel of surface plasmons had long been considered to be purely detrimental to the performance of plasmonic devices in applications such as waveguiding, sensing, and emission control. Recently, by harvesting highly energetic hot carriers generated from the nonradiative decay of surface plasmons, new applications in photocatalysis, photothermal heating, photovoltaics, and photodetection^49,463,464^ have been demonstrated. In this section, we review the recent progress in the exploitation of hot carrier-molecule interactions in plasmonic metamaterials for nanochemistry.

Compared with SPP-supporting metal films and LSP-supporting metal nanostructures, plasmonic metamaterials, which have the ability to provide larger surface areas as well as engineerable and greatly enhanced optical absorption,^467−469^ are more advantageous for the efficient generation and extraction of hot carriers to the immediate surroundings for nanochemistry. For example, employing a plasmonic metal-semiconductor-metal structure with TiO_2_ embedded between a gold mirror and gold nanoparticles, the enhanced broadband light absorption (>90%) was achieved, leading to a ∼40-fold increase of the photon-to-electron conversion efficiency with respect to a TiO_2_-supported gold nanoparticle monolayer.^468^ Therefore, plasmonic metamaterials provide an attractive platform for hot-carrier-based nanochemistry for water splitting, H_2_ dissociation, photocatalytic degradation of organic compounds, and CO_2_ reduction, to name but a few.

The harvesting of solar energy for water splitting to generate hydrogen has recently received considerable interest.^465,470−472^ In a typical experiment, an efficient, autonomous solar water-splitting device was demonstrated in which all charge carriers involved in the oxidation and reduction steps are derived from surface plasmons.^465^ The implemented device is based on a gold nanorod array serving as light-harvesting antenna capped with a thin layer of TiO_2_ which forms metal-semiconductor Schottky junction (Figure 25b). Pt nanoparticle-based hydrogen evolution catalyst and cobalt-based oxygen evolution catalyst (Co-OEC) were further deposited on the surface of TiO_2_ and the nanorod surfaces, respectively. A TEM image of an individual photosynthetic unit is presented in Figure 25c. Under light illumination, surface plasmons excited in the nanorod array generate hot electrons, a fraction of which can enter the conduction band of the TiO_2_ and be captured by Pt nanoparticles at the TiO_2_ surface, functioning as water reduction sites to generate H_2_. The positive charges (holes) in the gold nanorods, on the other hand, are transported to the Co-OEC, resulting in water oxidation reactions to generate O_2_. Therefore, the net result of this design is the direct splitting of water, with the charge carriers derived from surface plasmon decay with light being the only energy input. Under a constant illumination, the autonomous plasmonic solar splitter ran without noticeable degradation for more than 66 h (Figure 25d), with an external quantum efficiency reaching ∼0.25% at the plasmon resonance excitation. In another example, the enhanced water splitting was demonstrated under strong mode coupling conditions in a gold nanoparticle/TiO_2_/gold film structure.^466^ The partial inlay of a gold nanoparticle monolayer in the TiO_2_ layer (Figure 25e) enables a strong mode coupling between the Fabry–Perot nanocavity modes of the TiO_2_/gold film and LSPs of the gold nanoparticles, resulting in an obvious mode splitting in the absorption spectrum and, at same time, greatly enhanced absorption (more than 98%, as evidenced by the black appearance of the photographs of gold nanoparticle/TiO_2_/gold film structures in Figure 25f). Therefore, they observed an 11-fold increase in the incident photon-to-current conversion efficiency with respect to a photoanode structure without the gold film at the bottom. In a water-splitting experiment, H_2_ and O_2_ evolution on the Pt cathode and gold nanoparticle/TiO_2_/gold film photoanode increases linearly with the illumination time at the rates up to 90 and 43 nmol h^–1^ W^–1^ (Figure 25g), respectively, which are ∼6 fold higher than those from the Au-NP/TiO_2_ system.

Plasmonic metamaterials in a colloidal form, which have advantages such as broadband absorption, easy fabrication, and high surface-to-volume ratio, have also been investigated for hot-carrier-based nanochemistry.^473−477^ A plasmonic hot-carrier catalytic system based on silica nanoparticles decorated with plasmonic gold nanoparticles and platinum nanoclusters was developed which exhibits enhanced plasmon-induced photodegradation of methylene blue dyes that outperforms other photocatalysts.^477^

In addition to optical excitation by light illumination, hot electrons can also be generated electrically by tunneling electrons in tunnel junctions.^478,479^ Recent realization of the electrically driven plasmonic nanorod metamaterial provides the opportunity to use an electron tunneling effect for the simultaneous excitation of hot electrons and surface plasmons,^119,480^ opening a way to realize a new kind of hot-electron-activated nanoreactors, as well as highly compact and sensitive plasmonic sensors. By constructing a high-density array of plasmonic tunnel junctions at the top surface of a plasmonic metamaterial composed of vertically oriented gold nanorods, efficient electrical excitation of plasmonic modes of the metamaterial by inelastic electron tunneling was achieved (Figure 25h).^119^ Their radiative decay further produced an obvious light emission visible by the naked eye from the substrate side (Figure 25i). The spectrum of the emission in this case is shaped by the metamaterial plasmonic modes, which can be engineered throughout the visible and near-infrared ranges by tuning the metamaterial modes via the nanostructure geometric parameters.^481^ In addition to the excitation of plasmons by inelastic tunneling (with an efficiency around 0.1%), during the tunneling process, the majority of electrons (∼99.9%) tunnel elastically, appearing as energetic hot electrons in the nanorod tips (

The highly efficient and confined hot-electron generation makes the tunnel junctions highly reactive and opens up opportunities for the precise activation of chemical reactions in the junctions, which can be further detected with high sensitivity by observing the light emission from the tunnel junction or tunneling current changes due to the strong dependence of the highly confined tunneling process on any changes in the junction. This was demonstrated for both hydrogen and oxygen sensing using an electrically driven metamaterial with a monolayer of poly-l-histidine as a tunnel barrier, which was placd in a gas cell. When 2% H_2_ was introduced to replace air in the gas cell, the light emission intensity from the metamaterial decreased gradually to approximately one-half of the initial value (Figure 25j). When air containing oxygen was subsequently introduced into the gas cell, the emission intensity from the metamaterial increased gradually back to the initial value (Figure 25k). The sensing mechanism is based on hot electron-mediated oxidation and reduction of the tunnel junctions, which result in changes in the tunneling current and the emission intensity.^119^ Furthermore, promoting and stopping the reactions with the choice of the gas environment lead to the realization of the nonvolatile and multilevel memory device using reactive tunnel junctions.^480^ The information can be written into the junctions both electrically or optically via the hot-electron-mediated chemical reactions and read out by measuring the resistance or light emission. It has the potential to be used as multilevel nonvolatile memory, logic units, or artificial synapses in future optoelectronic and artificial neural networks.

## 9 Conclusion and Outlook

We have overviewed the research status of molecular plasmonics enabled by metamaterials. Thanks to the greatly enhanced light-molecule interactions and opportunities for flexible engineering of a resonant response, plasmonic metamaterials provide a versatile platform for the investigation of molecular plasmonics as well as the development of a variety of applications, including photoluminescence enhancement and engineering, optical modulation, high-sensitivity optical sensing, and photoactuated nanochemistry. There are still many challenges to be addressed for improving practical applications. In terms of plasmonic metamaterial fabrication, to achieve stronger light-molecule interaction and, therefore, better application performance, further advances in the quality of constituent meta-atoms to reduce optical losses and increase homogeneity of plasmonic metamaterials are always desirable. Mass production of plasmonic metamaterials is also required to reduce the cost of metamaterial-based photonic devices, which signifies the need for developing industrial-scale methods for the incorporation of molecular materials in the existing fabrication processes. For light modulators, incoherent light sources, and lasers, the challenges are related to the improvement of the stability of their characteristics and extension of their lifetimes. For optical sensing applications, the performance of biochemical sensors is usually characterized in a laboratory environment, which needs further evaluation in practical sensing scenarios with much more complex interference factors (e.g., temperature changes, the presence of other molecules, contaminations). In this case, despite the fact that the sensitivity of metamaterial-based biochemical sensors has reached an extremely low detection limit (e.g., picomole level), the specificity of the sensors still requires more attention, because the current specific binding approaches are limited to a small number of biochemical analytes. Integration of a metamaterial-based sensing element with a light source and a detector is also beneficial for the miniaturization of the metamaterial-based sensors, which are attractive for the development of lab-on-a-chip devices and wearable technology. Another challenge for metamaterial-based optical sensors is the protection of metamaterials from environmental contaminations while maintaining the high sensitivity to analytes in practical applications. For nanochemistry applications, further fundamental studies are needed to optimize the generation and extraction efficiencies of hot carriers.

In terms of the fundamental science of molecular plasmonics with metamaterials, several new directions are currently emerging. The first one is the development of new types of plasmonic metamaterials for molecular plasmonics, such as quantum plasmonic metamaterials and topological insulator-based metamaterials. Particularly, understanding and exploitation of nonlocal and quantum molecular plasmonics may open novel avenues across all the application areas, including engineered spontaneous emission and lasing, light modulation, and biochemical sensing. The exploitation of new plasmonic materials such as semiconductors and conducting oxides to extend the spectral range of light absorption and hot-electron generation is also important for optimal utilization of the solar spectrum. Research toward ultrastrong coupling of molecules with plasmonic excitations will lead to progress in understanding the fundamental physics of the process, enhancement of existing practical applications, and a search for new ones.

The use of molecules in combination with designer metamaterials has already provided many exquisite opportunities for active control of the optical properties of metamaterials using molecular processes as well as controlling molecular properties with metamaterials. Despite the above-mentioned challenges, as a versatile platform that can enhance and manipulate optical phenomena on the nanoscale, plasmonic metamaterials will continue to advance molecular plasmonics for the development of future photonic applications including ultrahigh-sensitivity optical sensing, nanolasers, active functionalities, and photoactuated nanochemistry.

## References

[ref1] Rivera N.; Kaminer I. Light–Matter Interactions with Photonic Quasiparticles. Nat. Rev. Phys. 2020, 2, 538–561. 10.1038/s42254-020-0224-2.

[ref2] Gutzler R.; Garg M.; Ast C. R.; Kuhnke K.; Kern K. Light–Matter Interaction at Atomic Scales. Nat. Rev. Phys. 2021, 3, 441–453. 10.1038/s42254-021-00306-5.

[ref3] Barnes W. L.; Dereux A.; Ebbesen T. W. Surface Plasmon Subwavelength Optics. Nature 2003, 424, 824–830. 10.1038/nature01937. 12917696

[ref4] Zayats A. V.; Smolyaninov I. I.; Maradudin A. A. Nano-Optics of Surface Plasmon Polaritons. Phys. Rep. 2005, 408, 131–314. 10.1016/j.physrep.2004.11.001.

[ref5] Giannini V.; Fernández-Domínguez A. I.; Heck S. C.; Maier S. A. Plasmonic Nanoantennas: Fundamentals and Their Use in Controlling the Radiative Properties of Nanoemitters. Chem. Rev. 2011, 111, 3888–3912. 10.1021/cr1002672. 21434605

[ref6] Schuller J. A.; Barnard E. S.; Cai W.; Jun Y. C.; White J. S.; Brongersma M. L. Plasmonics for Extreme Light Concentration and Manipulation. Nat. Mater. 2010, 9, 193–204. 10.1038/nmat2630. 20168343

[ref7] Chikkaraddy R.; de Nijs B.; Benz F.; Barrow S. J.; Scherman O. A.; Rosta E.; Demetriadou A.; Fox P.; Hess O.; Baumberg J. J. Single-Molecule Strong Coupling at Room Temperature in Plasmonic Nanocavities. Nature 2016, 535, 127–130. 10.1038/nature17974. 27296227 PMC4947385

[ref8] Baumberg J. J.; Aizpurua J.; Mikkelsen M. H.; Smith D. R. Extreme Nanophotonics from Ultrathin Metallic Gaps. Nat. Mater. 2019, 18, 668–678. 10.1038/s41563-019-0290-y. 30936482

[ref9] Lal S.; Link S.; Halas N. J. Nano-Optics from Sensing to Waveguiding. Nat. Photonics 2007, 1, 641–648. 10.1038/nphoton.2007.223.

[ref10] Krasavin A. V.; Zayats A. V. Guiding Light at the Nanoscale: Numerical Optimization of Ultrasubwavelength Metallic Wire Plasmonic Waveguides. Opt. Lett. 2011, 36, 3127–3129. 10.1364/OL.36.003127. 21847182

[ref11] Wei H.; Pan D.; Zhang S.; Li Z.; Li Q.; Liu N.; Wang W.; Xu H. Plasmon Waveguiding in Nanowires. Chem. Rev. 2018, 118, 2882–2926. 10.1021/acs.chemrev.7b00441. 29446301

[ref12] Homola J. Surface Plasmon Resonance Sensors for Detection of Chemical and Biological Species. Chem. Rev. 2008, 108, 462–493. 10.1021/cr068107d. 18229953

[ref13] Wang P.; Nasir M. E.; Krasavin A. V.; Dickson W.; Jiang Y.; Zayats A. V. Plasmonic Metamaterials for Nanochemistry and Sensing. Acc. Chem. Rev. 2019, 52, 3018–3028. 10.1021/acs.accounts.9b00325. 31680511

[ref14] Krasavin A. V.; Zheludev N. I. Active Plasmonics: Controlling Signals in Au/Ga Waveguide Using Nanoscale Structural Transformations. Appl. Phys. Lett. 2004, 84, 1416–1418. 10.1063/1.1650904.

[ref15] Wurtz G. A.; Pollard R.; Hendren W.; Wiederrecht G. P.; Gosztola D. J.; Podolskiy V. A.; Zayats A. V. Designed Ultrafast Optical Nonlinearity in a Plasmonic Nanorod Metamaterial Enhanced by Nonlocality. Nat. Nanotechnol. 2011, 6, 107–111. 10.1038/nnano.2010.278. 21258335

[ref16] Ayata M.; Fedoryshyn Y.; Heni W.; Baeuerle B.; Josten A.; Zahner M.; Koch U.; Salamin Y.; Hoessbacher C.; Haffner C.; et al. High-Speed Plasmonic Modulator in a Single Metal Layer. Science 2017, 358, 630–632. 10.1126/science.aan5953. 29097545

[ref17] Haffner C.; Chelladurai D.; Fedoryshyn Y.; Josten A.; Baeuerle B.; Heni W.; Watanabe T.; Cui T.; Cheng B.; Saha S.; et al. Low-Loss Plasmon-Assisted Electro-Optic Modulator. Nature 2018, 556, 483–486. 10.1038/s41586-018-0031-4. 29695845 PMC5935232

[ref18] Mühlschlegel P.; Eisler H. J.; Martin O. J. F.; Hecht B.; Pohl D. W. Resonant Optical Antennas. Science 2005, 308, 1607–1609. 10.1126/science.1111886. 15947182

[ref19] Kauranen M.; Zayats A. V. Nonlinear Plasmonics. Nat. Photonics 2012, 6, 737–748. 10.1038/nphoton.2012.244.

[ref20] Oulton R. F.; Sorger V. J.; Zentgraf T.; Ma R.-M.; Gladden C.; Dai L.; Bartal G.; Zhang X. Plasmon Lasers at Deep Subwavelength Scale. Nature 2009, 461, 629–632. 10.1038/nature08364. 19718019

[ref21] Wu H.; Gao Y.; Xu P.; Guo X.; Wang P.; Dai D.; Tong L. Plasmonic Nanolasers: Pursuing Extreme Lasing Conditions on Nanoscale. Adv. Opt. Mater. 2019, 7, 1900334 10.1002/adom.201900334.

[ref22] Fedyanin D. Y.; Krasavin A. V.; Arsenin A. V.; Zayats A. V. Lasing at the Nanoscale: Coherent Emission of Surface Plasmons by an Electrically Driven Nanolaser. Nanophotonics 2020, 9, 3965–3975. 10.1515/nanoph-2020-0157.

[ref23] Ma R.-M.; Wang S.-Y. Plasmonic Nanolasers: Fundamental Properties and Applications. Nanophotonics 2021, 10, 3623–3633. 10.1515/nanoph-2021-0298.

[ref24] Lu X.; Rycenga M.; Skrabalak S. E.; Wiley B.; Xia Y. Chemical Synthesis of Novel Plasmonic Nanoparticles. Annu. Rev. Phys. Chem. 2009, 60, 167–192. 10.1146/annurev.physchem.040808.090434. 18976140

[ref25] Romo-Herrera J. M.; Alvarez-Puebla R. A.; Liz-Marzán L. M. Controlled Assembly of Plasmonic Colloidal Nanoparticle Clusters. Nanoscale 2011, 3, 1304–1315. 10.1039/c0nr00804d. 21229160

[ref26] Córdova-Castro R. M.; Casavola M.; van Schilfgaarde M.; Krasavin A. V.; Green M. A.; Richards D.; Zayats A. V. Anisotropic Plasmonic CuS Nanocrystals as a Natural Electronic Material with Hyperbolic Optical Dispersion. ACS Nano 2019, 13, 6550–6560. 10.1021/acsnano.9b00282. 31117375

[ref27] Liu L.; Krasavin A. V.; Zheng J.; Tong Y.; Wang P.; Wu X.; Hecht B.; Pan C.; Li J.; Li L.; et al. Atomically Smooth Single-Crystalline Platform for Low-Loss Plasmonic Nanocavities. Nano Lett. 2022, 22, 1786–1794. 10.1021/acs.nanolett.2c00095. 35129980

[ref28] Huo D.; Kim M. J.; Lyu Z.; Shi Y.; Wiley B. J.; Xia Y. One-Dimensional Metal Nanostructures: From Colloidal Syntheses to Applications. Chem. Rev. 2019, 119, 8972–9073. 10.1021/acs.chemrev.8b00745. 30854849

[ref29] Kasani S.; Curtin K.; Wu N. A Review of 2D and 3D Plasmonic Nanostructure Array Patterns: Fabrication, Light Management and Sensing Applications. Nanophotonics 2019, 8, 2065–2089. 10.1515/nanoph-2019-0158.

[ref30] Yang Y.; Gu C.; Li J. Sub-5 Nm Metal Nanogaps: Physical Properties, Fabrication Methods, and Device Applications. Small 2019, 15, 1804177 10.1002/smll.201804177. 30589217

[ref31] Luo S.; Hoff B. H.; Maier S. A.; de Mello J. C. Scalable Fabrication of Metallic Nanogaps at the Sub-10 nm Level. Adv. Sci. 2021, 8, 2102756 10.1002/advs.202102756. PMC8693066 34719889

[ref32] Zheng J.; Cheng X.; Zhang H.; Bai X.; Ai R.; Shao L.; Wang J. Gold Nanorods: The Most Versatile Plasmonic Nanoparticles. Chem. Rev. 2021, 121, 13342–13453. 10.1021/acs.chemrev.1c00422. 34569789

[ref33] Liu Y.; Zhang X. Metamaterials: A New Frontier of Science and Technology. Chem. Soc. Rev. 2011, 40, 2494–2507. 10.1039/c0cs00184h. 21234491

[ref34] Hess O.; Pendry J. B.; Maier S. A.; Oulton R. F.; Hamm J. M.; Tsakmakidis K. L. Active Nanoplasmonic Metamaterials. Nat. Mater. 2012, 11, 573–584. 10.1038/nmat3356. 22717488

[ref35] Poddubny A.; Iorsh I.; Belov P.; Kivshar Y. Hyperbolic Metamaterials. Nat. Photonics 2013, 7, 948–957. 10.1038/nphoton.2013.243.

[ref36] Huo P.; Zhang S.; Liang Y.; Lu Y.; Xu T. Hyperbolic Metamaterials and Metasurfaces: Fundamentals and Applications. Adv. Opt. Mater. 2019, 7, 1801616 10.1002/adom.201801616.

[ref37] Pendry J. B.; Holden A. J.; Stewart W. J.; Youngs I. Extremely Low Frequency Plasmons in Metallic Mesostructures. Phys. Rev. Lett. 1996, 76, 4773–4776. 10.1103/PhysRevLett.76.4773. 10061377

[ref38] Yen T. J.; Padilla W. J.; Fang N.; Vier D. C.; Smith D. R.; Pendry J. B.; Basov D. N.; Zhang X. Terahertz Magnetic Response from Artificial Materials. Science 2004, 303, 1494–1496. 10.1126/science.1094025. 15001772

[ref39] Giovannetti V.; Lloyd S.; Maccone L. Quantum-Enhanced Measurements: Beating the Standard Quantum Limit. Science 2004, 306, 1330–1336. 10.1126/science.1104149. 15550661

[ref40] Smith D. R.; Padilla W. J.; Vier D. C.; Nemat-Nasser S. C.; Schultz S. Composite Medium with Simultaneously Negative Permeability and Permittivity. Phys. Rev. Lett. 2000, 84, 4184–4187. 10.1103/PhysRevLett.84.4184. 10990641

[ref41] Dolling G.; Enkrich C.; Wegener M.; Soukoulis C. M.; Linden S. Simultaneous Negative Phase and Group Velocity of Light in a Metamaterial. Science 2006, 312, 892–894. 10.1126/science.1126021. 16690860

[ref42] Soukoulis C. M.; Linden S.; Wegener M. Negative Refractive Index at Optical Wavelengths. Science 2007, 315, 47–49. 10.1126/science.1136481. 17204630

[ref43] Yang X.; Yao J.; Rho J.; Yin X.; Zhang X. Experimental Realization of Three-Dimensional Indefinite Cavities at the Nanoscale with Anomalous Scaling Laws. Nat. Photonics 2012, 6, 450–454. 10.1038/nphoton.2012.124.

[ref44] Nicholls L. H.; Rodríguez-Fortuño F. J.; Nasir M. E.; Córdova-Castro R. M.; Olivier N.; Wurtz G. A.; Zayats A. V. Ultrafast Synthesis and Switching of Light Polarization in Nonlinear Anisotropic Metamaterials. Nat. Photonics 2017, 11, 628–633. 10.1038/s41566-017-0002-6.

[ref45] Gansel J. K.; Thiel M.; Rill M. S.; Decker M.; Bade K.; Saile V.; von Freymann G.; Linden S.; Wegener M. Gold Helix Photonic Metamaterial as Broadband Circular Polarizer. Science 2009, 325, 1513–1515. 10.1126/science.1177031. 19696310

[ref46] Vestler D.; Shishkin I.; Gurvitz E. A.; Nasir M. E.; Ben-Moshe A.; Slobozhanyuk A. P.; Krasavin A. V.; Levi-Belenkova T.; Shalin A. S.; Ginzburg P.; et al. Circular Dichroism Enhancement in Plasmonic Nanorod Metamaterials. Opt. Express 2018, 26, 17841–17848. 10.1364/OE.26.017841. 30114069

[ref47] Ginzburg P.; Krasavin A. V.; Poddubny A. N.; Belov P. A.; Kivshar Y. S.; Zayats A. V. Self-Induced Torque in Hyperbolic Metamaterials. Phys. Rev. Lett. 2017, 111, 036804 10.1103/PhysRevLett.111.036804. 23909352

[ref48] Ivinskaya A.; Kostina N.; Proskurin A.; Petrov M. I.; Bogdanov A. A.; Sukhov S.; Krasavin A. V.; Karabchevsky A.; Shalin A. S.; Ginzburg P. Optomechanical Manipulation with Hyperbolic Metasurfaces. ACS Photonics 2018, 5, 4371–4377. 10.1021/acsphotonics.8b00775.

[ref49] Brongersma M. L.; Halas N. J.; Nordlander P. Plasmon-Induced Hot Carrier Science and Technology. Nat. Nanotechnol. 2015, 10, 25–34. 10.1038/nnano.2014.311. 25559968

[ref50] Vaskin A.; Kolkowski R.; Koenderink A. F.; Staude I. Light-Emitting Metasurfaces. Nanophotonics 2019, 8, 1151–1198. 10.1515/nanoph-2019-0110.

[ref51] Yu N.; Capasso F. Flat Optics with Designer Metasurfaces. Nat. Mater. 2014, 13, 139–150. 10.1038/nmat3839. 24452357

[ref52] Pollard R. J.; Murphy A.; Hendren W. R.; Evans P. R.; Atkinson R.; Wurtz G. A.; Zayats A. V.; Podolskiy V. A. Optical Nonlocalities and Additional Waves in Epsilon-near-Zero Metamaterials. Phys. Rev. Lett. 2009, 102, 127405 10.1103/PhysRevLett.102.127405. 19392325

[ref53] Ginzburg P.; Roth D. J.; Nasir M. E.; Segovia P.; Krasavin A. V.; Levitt J.; Hirvonen L. M.; Wells B.; Suhling K.; Richards D.; et al. Spontaneous Emission in Non-Local Materials. Light Sci. Appl. 2017, 6, e16273–e16273. 10.1038/lsa.2016.273. 30167260 PMC6062244

[ref54] Yariv A.; Yeh P. Photonics: Optical Electronics in Modern Communications; Oxford University Press, 2007; pp 43–44.

[ref55] Elser J.; Wangberg R.; Podolskiy V. A.; Narimanov E. E. Nanowire Metamaterials with Extreme Optical Anisotropy. Appl. Phys. Lett. 2006, 89, 261102 10.1063/1.2422893.

[ref56] Wells B. M.; Zayats A. V.; Podolskiy V. A. Nonlocal Optics of Plasmonic Nanowire Metamaterials. Phys. Rev. B 2014, 89, 035111 10.1103/PhysRevB.89.035111.

[ref57] Agranovich V. M.; Kravtsov V. E. Notes on Crystal Optics of Superlattices. Solid State Commun. 1985, 55, 85–90. 10.1016/0038-1098(85)91111-1.

[ref58] Drachev V. P.; Podolskiy V. A.; Kildishev A. V. Hyperbolic Metamaterials: New Physics Behind a Classical Problem. Opt. Express 2013, 21, 15048–15064. 10.1364/OE.21.015048. 23787692

[ref59] Sun L.; Li Z. G.; Luk T. S.; Yang X. D.; Gao J. Nonlocal Effective Medium Analysis in Symmetric Metal-Dielectric Multilayer Metamaterials. Phys. Rev. B 2015, 91, 195147 10.1103/PhysRevB.91.195147.

[ref60] Li L.; Wang W.; Luk T. S.; Yang X. D.; Gao J. Enhanced Quantum Dot Spontaneous Emission with Multilayer Metamaterial Nanostructures. ACS Photonics 2017, 4, 501–508. 10.1021/acsphotonics.6b01039.

[ref61] Gorlach M. A.; Lapine M. Boundary Conditions for the Effective-Medium Description of Subwavelength Multilayered Structures. Phys. Rev. B 2020, 101, 075127 10.1103/PhysRevB.101.075127.

[ref62] Krasavin A. V.; Ginzburg P.; Zayats A. V. Free-Electron Optical Nonlinearities in Plasmonic Nanostructures: A Review of the Hydrodynamic Description. Laser Photonics Rev. 2018, 12, 1700082 10.1002/lpor.201700082.

[ref63] Krasavin A. V. A Brief Review on Optical Properties of Planar Metallic Interfaces and Films: From Classical View to Quantum Description. J. Phys. Photonics 2021, 3, 042006 10.1088/2515-7647/ac2569.

[ref64] Javani M. H.; Stockman M. I. Real and Imaginary Properties of Epsilon-near-Zero Materials. Phys. Rev. Lett. 2016, 117, 107404 10.1103/PhysRevLett.117.107404. 27636495

[ref65] Neira A. D.; Olivier N.; Nasir M. E.; Dickson W.; Wurtz G. A.; Zayats A. V. Eliminating Material Constraints for Nonlinearity with Plasmonic Metamaterials. Nat. Commun. 2015, 6, 7757 10.1038/ncomms8757. 26195182 PMC4518246

[ref66] Wells B.; Bykov A. Y.; Marino G.; Nasir M. E.; Zayats A. V.; Podolskiy V. A. Structural Second-Order Nonlinearity in Plasmonic Metamaterials. Optica 2018, 5, 1502–1507. 10.1364/OPTICA.5.001502.

[ref67] Roth D. J.; Krasavin A. V.; Wade A.; Dickson W.; Murphy A.; Kena-Cohen S.; Pollard R.; Wurtz G. A.; Richards D.; Maier S. A.; et al. Spontaneous Emission inside a Hyperbolic Metamaterial Waveguide. ACS Photonics 2017, 4, 2513–2521. 10.1021/acsphotonics.7b00767.

[ref68] Marino G.; Segovia P.; Krasavin A. V.; Ginzburg P.; Olivier N.; Wurtz G. A.; Zayats A. V. Second-Harmonic Generation from Hyperbolic Plasmonic Nanorod Metamaterial Slab. Laser Photonics Rev. 2018, 12, 1700189 10.1002/lpor.201700189.

[ref69] High A. A.; Devlin R. C.; Dibos A.; Polking M.; Wild D. S.; Perczel J.; de Leon N. P.; Lukin M. D.; Park H. Visible-Frequency Hyperbolic Metasurface. Nature 2015, 522, 192–196. 10.1038/nature14477. 26062510

[ref70] Kuzmin D. A.; Bychkov I. V.; Shavrov V. G.; Temnov V. V. Hyperbolic Plasmonics with Anisotropic Gain-Loss Metasurfaces. Opt. Lett. 2021, 46, 420–423. 10.1364/OL.413511. 33449044

[ref71] Ferrari L.; Wu C. H.; Lepage D.; Zhang X.; Liu Z. W. Hyperbolic Metamaterials and Their Applications. Prog. Quantum. Electron. 2015, 40, 1–40. 10.1016/j.pquantelec.2014.10.001.

[ref72] Córdova-Castro R. M.; Krasavin A. V.; Nasir M. E.; Zayats A. V.; Dickson W. Nanocone-Based Plasmonic Metamaterials. Nanotechnology 2019, 30, 055301 10.1088/1361-6528/aaea39. 30521490

[ref73] Yu N.; Genevet P.; Kats M. A.; Aieta F.; Tetienne J.-P.; Capasso F.; Gaburro Z. Light Propagation with Phase Discontinuities: Generalized Laws of Reflection and Refraction. Science 2011, 334, 333–337. 10.1126/science.1210713. 21885733

[ref74] Liu N.; Guo H.; Fu L.; Kaiser S.; Schweizer H.; Giessen H. Three-Dimensional Photonic Metamaterials at Optical Frequencies. Nat. Mater. 2008, 7, 31–37. 10.1038/nmat2072. 18059275

[ref75] Liu N.; Liu H.; Zhu S.; Giessen H. Stereometamaterials. Nat. Photonics 2009, 3, 157–162. 10.1038/nphoton.2009.4.

[ref76] Kante B.; Park Y. S.; O’Brien K.; Shuldman D.; Lanzillotti-Kimura N. D.; Wong Z. J.; Yin X. B.; Zhang X. Symmetry Breaking and Optical Negative Index of Closed Nanorings. Nat. Commun. 2012, 3, 1180 10.1038/ncomms2161. 23149726

[ref77] Cui A. J.; Liu Z.; Li J. F.; Shen T. H. H.; Xia X. X.; Li Z. Y.; Gong Z. J.; Li H. Q.; Wang B. L.; Li J. J.; et al. Directly Patterned Substrate-Free Plasmonic ″Nanograter” Structures with Unusual Fano Resonances. Light Sci. Appl. 2015, 4, e308 10.1038/lsa.2015.81.

[ref78] Fan J. A.; Wu C. H.; Bao K.; Bao J. M.; Bardhan R.; Halas N. J.; Manoharan V. N.; Nordlander P.; Shvets G.; Capasso F. Self-Assembled Plasmonic Nanoparticle Clusters. Science 2010, 328, 1135–1138. 10.1126/science.1187949. 20508125

[ref79] Mueller N. S.; Okamura Y.; Vieira B. G. M.; Juergensen S.; Lange H.; Barros E. B.; Schulz F.; Reich S. Deep Strong Light-Matter Coupling in Plasmonic Nanoparticle Crystals. Nature 2020, 583, 780 10.1038/s41586-020-2508-1. 32728238

[ref80] Kuzyk A.; Schreiber R.; Fan Z. Y.; Pardatscher G.; Roller E. M.; Hogele A.; Simmel F. C.; Govorov A. O.; Liedl T. DNA-Based Self-Assembly of Chiral Plasmonic Nanostructures with Tailored Optical Response. Nature 2012, 483, 311–314. 10.1038/nature10889. 22422265

[ref81] Li P.; Chen S.; Dai H.; Yang Z.; Chen Z.; Wang Y.; Chen Y.; Peng W.; Shan W.; Duan H. Recent Advances in Focused Ion Beam Nanofabrication for Nanostructures and Devices: Fundamentals and Applications. Nanoscale 2021, 13, 1529–1565. 10.1039/D0NR07539F. 33432962

[ref82] Lu D.; Kan J. J.; Fullerton E. E.; Liu Z. W. Enhancing Spontaneous Emission Rates of Molecules Using Nanopatterned Multilayer Hyperbolic Metamaterials. Nat. Nanotechnol. 2014, 9, 48–53. 10.1038/nnano.2013.276. 24390565

[ref83] Lu D.; Qian H. L.; Wang K. W.; Shen H.; Wei F. F.; Jiang Y. F.; Fullerton E. E.; Yu P. K. L.; Liu Z. W. Nanostructuring Multilayer Hyperbolic Metamaterials for Ultrafast and Bright Green Ingan Quantum Wells. Adv. Mater. 2018, 30, 1706411 10.1002/adma.201706411. 29512215

[ref84] Isoniemi T.; Maccaferri N.; Ramasse Q. M.; Strangi G.; De Angelis F. Electron Energy Loss Spectroscopy of Bright and Dark Modes in Hyperbolic Metamaterial Nanostructures. Adv. Opt. Mater. 2020, 8, 2000277 10.1002/adom.202000277.

[ref85] Liu Z. G.; Du H. F.; Li J. F.; Lu L.; Li Z. Y.; Fang N. X. Nano-Kirigami with Giant Optical Chirality. Sci. Adv. 2018, 4, eaat4436 10.1126/sciadv.aat4436. 29984308 PMC6035038

[ref86] Li J. F.; Liu Z. G. Focused-Ion-Beam-Based Nano-Kirigami: From Art to Photonics. Nanophotonics 2018, 7, 1637–1650. 10.1515/nanoph-2018-0117.

[ref87] Manoccio M.; Esposito M.; Passaseo A.; Cuscuna M.; Tasco V. Focused Ion Beam Processing for 3D Chiral Photonics Nanostructures. Micromachines 2021, 12, 6 10.3390/mi12010006. PMC7823276 33374782

[ref88] Kollmann H.; Piao X.; Esmann M.; Becker S. F.; Hou D. C.; Huynh C.; Kautschor L. O.; Bosker G.; Vieker H.; Beyer A.; et al. Toward Plasmonics with Nanometer Precision: Nonlinear Optics of Helium-Ion Milled Gold Nanoantennas. Nano Lett. 2014, 14, 4778–4784. 10.1021/nl5019589. 25051422

[ref89] Kawata S.; Sun H. B.; Tanaka T.; Takada K. Finer Features for Functional Microdevices - Micromachines Can Be Created with Higher Resolution Using Two-Photon Absorption. Nature 2001, 412, 697–698. 10.1038/35089130. 11507627

[ref90] Deubel M.; Von Freymann G.; Wegener M.; Pereira S.; Busch K.; Soukoulis C. M. Direct Laser Writing of Three-Dimensional Photonic-Crystal Templates for Telecommunications. Nat. Mater. 2004, 3, 444–447. 10.1038/nmat1155. 15195083

[ref91] Radke A.; Gissibl T.; Klotzbucher T.; Braun P. V.; Giessen H. Three-Dimensional Bichiral Plasmonic Crystals Fabricated by Direct Laser Writing and Electroless Silver Plating. Adv. Mater. 2011, 23, 3018 10.1002/adma.201100543. 21509833

[ref92] Fischer J.; von Freymann G.; Wegener M. The Materials Challenge in Diffraction-Unlimited Direct-Laser-Writing Optical Lithography. Adv. Mater. 2010, 22, 3578 10.1002/adma.201000892. 20593434

[ref93] Fischer J.; Wegener M. Three-Dimensional Direct Laser Writing Inspired by Stimulated-Emission-Depletion Microscopy. Opt. Mater. Express 2011, 1, 614–624. 10.1364/OME.1.000614.

[ref94] Kaschke J.; Blume L.; Wu L.; Thiel M.; Bade K.; Yang Z. Y.; Wegener M. A Helical Metamaterial for Broadband Circular Polarization Conversion. Adv. Opt. Mater. 2015, 3, 1411–1417. 10.1002/adom.201500194.

[ref95] Kaschke J.; Wegener M. Gold Triple-Helix Mid-Infrared Metamaterial by STED-Inspired Laser Lithography. Opt. Lett. 2015, 40, 3986–3989. 10.1364/OL.40.003986. 26368693

[ref96] Li P.; Li Y.; Zhou Z.-K.; Tang S.; Yu X.-F.; Xiao S.; Wu Z.; Xiao Q.; Zhao Y.; Wang H.; et al. Evaporative Self-Assembly of Gold Nanorods into Macroscopic 3D Plasmonic Superlattice Arrays. Adv. Mater. 2016, 28, 2511–2517. 10.1002/adma.201505617. 26823278

[ref97] Liu N.; Liedl T. DNA-Assembled Advanced Plasmonic Architectures. Chem. Rev. 2018, 118, 3032–3053. 10.1021/acs.chemrev.7b00225. 29384370 PMC6546600

[ref98] Schulz F.; Pavelka O.; Lehmkühler F.; Westermeier F.; Okamura Y.; Mueller N. S.; Reich S.; Lange H. Structural Order in Plasmonic Superlattices. Nat. Commun. 2020, 11, 3821 10.1038/s41467-020-17632-4. 32732893 PMC7393164

[ref99] Edel J. B.; Kornyshev A. A.; Kucernak A. R.; Urbakh M. Fundamentals and Applications of Self-Assembled Plasmonic Nanoparticles at Interfaces. Chem. Soc. Rev. 2016, 45, 1581–1596. 10.1039/C5CS00576K. 26806599

[ref100] Montelongo Y.; Sikdar D.; Ma Y.; McIntosh A. J. S.; Velleman L.; Kucernak A. R.; Edel J. B.; Kornyshev A. A. Electrotunable Nanoplasmonic Liquid Mirror. Nat. Mater. 2017, 16, 1127 10.1038/nmat4969. 28892055

[ref101] Sikdar D.; Bucher A.; Zagar C.; Kornyshev A. A. Electrochemical Plasmonic Metamaterials: Towards Fast Electro-Tuneable Reflecting Nanoshutters. Faraday Discuss. 2017, 199, 585–602. 10.1039/C6FD00249H. 28429003

[ref102] Ma Y.; Zagar C.; Klemme D. J.; Sikdar D.; Velleman L.; Montelongo Y.; Oh S. H.; Kucernak A. R.; Edel J. B.; Kornyshev A. A. A Tunable Nanoplasmonic Mirror at an Electrochemical Interface. ACS Photon. 2018, 5, 4604–4616. 10.1021/acsphotonics.8b01105.

[ref103] Jin Y.; Zhou L.; Liang J.; Zhu J. Electrochemically Driven Dynamic Plasmonics. Adv. Photon. 2021, 3, 044002 10.1117/1.AP.3.4.044002.

[ref104] Alivisatos A. P.; Johnsson K. P.; Peng X. G.; Wilson T. E.; Loweth C. J.; Bruchez M. P.; Schultz P. G. Organization of ’Nanocrystal Molecules’ Using DNA. Nature 1996, 382, 609–611. 10.1038/382609a0. 8757130

[ref105] Mirkin C. A.; Letsinger R. L.; Mucic R. C.; Storhoff J. J. A DNA-Based Method for Rationally Assembling Nanoparticles into Macroscopic Materials. Nature 1996, 382, 607–609. 10.1038/382607a0. 8757129

[ref106] Mastroianni A. J.; Claridge S. A.; Alivisatos A. P. Pyramidal and Chiral Groupings of Gold Nanocrystals Assembled Using DNA Scaffolds. J. Am. Chem. Soc. 2009, 131, 8455–8459. 10.1021/ja808570g. 19331419 PMC2767249

[ref107] Tan S. J.; Campolongo M. J.; Luo D.; Cheng W. L. Building Plasmonic Nanostructures with DNA. Nat. Nanotechnol. 2011, 6, 268–276. 10.1038/nnano.2011.49. 21499251

[ref108] Chao J.; Lin Y. F.; Liu H. J.; Wang L. H.; Fan C. H. DNA-Based Plasmonic Nanostructures. Mater. Today 2015, 18, 326–335. 10.1016/j.mattod.2015.01.018.

[ref109] Rothemund P. W. K. Folding DNA to Create Nanoscale Shapes and Patterns. Nature 2006, 440, 297–302. 10.1038/nature04586. 16541064

[ref110] Douglas S. M.; Dietz H.; Liedl T.; Hogberg B.; Graf F.; Shih W. M. Self-Assembly of DNA into Nanoscale Three-Dimensional Shapes. Nature 2009, 459, 414–418. 10.1038/nature08016. 19458720 PMC2688462

[ref111] Young K. L.; Ross M. B.; Blaber M. G.; Rycenga M.; Jones M. R.; Zhang C.; Senesi A. J.; Lee B.; Schatz G. C.; Mirkin C. A. Using DNA to Design Plasmonic Metamaterials with Tunable Optical Properties. Adv. Mater. 2014, 26, 653–659. 10.1002/adma.201302938. 24166990

[ref112] Wang P. F.; Gaitanaros S.; Lee S.; Bathe M.; Shih W. M.; Ke Y. G. Programming Self-Assembly of DNA Origami Honeycomb Two-Dimensional Lattices and Plasmonic Metamaterials. J. Am. Chem. Soc. 2016, 138, 7733–7740. 10.1021/jacs.6b03966. 27224641

[ref113] Foss C. A.; Hornyak G. L.; Stockert J. A.; Martin C. R. Template-Synthesized Nanoscopic Gold Particles: Optical Spectra and the Effects of Particle Size and Shape. J. Phys. Chem. 1994, 98, 2963–2971. 10.1021/j100062a037.

[ref114] Su Z. X.; Zhou W. Z. Formation Mechanism of Porous Anodic Aluminium and Titanium Oxides. Adv. Mater. 2008, 20, 3663 10.1002/adma.200800845.

[ref115] Martin C. R. Nanomaterials: A Membrane-Based Synthetic Approach. Science 1994, 266, 1961–1966. 10.1126/science.266.5193.1961. 17836514

[ref116] Sander M. S.; Tan L. S. Nanoparticle Arrays on Surfaces Fabricated Using Anodic Alumina Films as Templates. Adv. Funct. Mater. 2003, 13, 393–397. 10.1002/adfm.200304290.

[ref117] Evans P.; Hendren W. R.; Atkinson R.; Wurtz G. A.; Dickson W.; Zayats A. V.; Pollard R. J. Growth and Properties of Gold and Nickel Nanorods in Thin Film Alumina. Nanotechnology 2006, 17, 5746–5753. 10.1088/0957-4484/17/23/006.

[ref118] Nasir M. E.; Peruch S.; Vasilantonakis N.; Wardley W. P.; Dickson W.; Wurtz G. A.; Zayats A. V. Tuning the Effective Plasma Frequency of Nanorod Metamaterials from Visible to Telecom Wavelengths. Appl. Phys. Lett. 2015, 107, 121110 10.1063/1.4931687.

[ref119] Wang P.; Krasavin A. V.; Nasir M. E.; Dickson W.; Zayats A. V. Reactive Tunnel Junctions in Electrically Driven Plasmonic Nanorod Metamaterials. Nat. Nanotechnol. 2018, 13, 159–164. 10.1038/s41565-017-0017-7. 29230044 PMC5805091

[ref120] Cheng Z. Q.; Nan F.; Yang D. J.; Zhong Y. T.; Ma L.; Hao Z. H.; Zhou L.; Wang Q. Q. Plasmonic Nanorod Arrays of a Two-Segment Dimer and a Coaxial Cable with 1 Nm Gap for Large Field Confinement and Enhancement. Nanoscale 2015, 7, 1463–1470. 10.1039/C4NR05544F. 25503522

[ref121] Nasir M. E.; Krasavin A. V.; Cordova-Castro R. M.; McPolin C. P. T.; Bouillard J. S. G.; Wang P.; Zayats A. V. Mode Engineering in Large Arrays of Coupled Plasmonic-Dielectric Nanoantennas. Adv. Opt. Mater. 2021, 9, 2001467 10.1002/adom.202001467.

[ref122] Yakovlev V. V.; Dickson W.; Murphy A.; McPhillips J.; Pollard R. J.; Podolskiy V. A.; Zayats A. V. Ultrasensitive Non-Resonant Detection of Ultrasound with Plasmonic Metamaterials. Adv. Mater. 2013, 25, 2351–2356. 10.1002/adma.201300314. 23450522

[ref123] Nasir M. E.; Dickson W.; Wurtz G. A.; Wardley W. P.; Zayats A. V. Hydrogen Detected by the Naked Eye: Optical Hydrogen Gas Sensors Based on Core/Shell Plasmonic Nanorod Metamaterials. Adv. Mater. 2014, 26, 3532–3537. 10.1002/adma.201305958. 24643991

[ref124] Fan B.; Nasir M. E.; Nicholls L. H.; Zayats A. V.; Podolskiy V. A. Magneto-Optical Metamaterials: Nonreciprocal Transmission and Faraday Effect Enhancement. Adv. Opt. Mater. 2019, 7, 1801420 10.1002/adom.201801420.

[ref125] McPhillips J.; Murphy A.; Jonsson M. P.; Hendren W. R.; Atkinson R.; Hook F.; Zayats A. V.; Pollard R. J. High-Performance Biosensing Using Arrays of Plasmonic Nanotubes. ACS Nano 2010, 4, 2210–2216. 10.1021/nn9015828. 20218668

[ref126] Murphy A.; McPhillips J.; Hendren W.; McClatchey C.; Atkinson R.; Wurtz G.; Zayats A. V.; Pollard R. J. The Controlled Fabrication and Geometry Tunable Optics of Gold Nanotube Arrays. Nanotechnology 2011, 22, 045705 10.1088/0957-4484/22/4/045705. 21169660

[ref127] Murphy A.; Sonnefraud Y.; Krasavin A. V.; Ginzburg P.; Morgan F.; McPhillips J.; Wurtz G.; Maier S. A.; Zayats A. V.; Pollard R. Fabrication and Optical Properties of Large-Scale Arrays of Gold Nanocavities Based on Rod-in-a-Tube Coaxials. Appl. Phys. Lett. 2013, 102, 103103 10.1063/1.4794935.

[ref128] Tsai K.-T.; Wurtz G. A.; Chu J.-Y.; Cheng T.-Y.; Wang H.-H.; Krasavin A. V.; He J.-H.; Wells B. M.; Podolskiy V. A.; Wang J.-K.; et al. Looking into Meta-Atoms of Plasmonic Nanowire Metamaterial. Nano Lett. 2014, 14, 4971–4976. 10.1021/nl501283c. 25115592

[ref129] Yan R. Q.; Wang T.; Yue X. Z.; Wang H. M.; Zhang Y. H.; Xu P.; Wang L.; Wang Y. D.; Zhang J. Y. Highly Sensitive Plasmonic Nanorod Hyperbolic Metamaterial Biosensor. Photon. Res. 2022, 10, 84–95. 10.1364/PRJ.444490.

[ref130] Wu W.; Yu Z.; Wang S.-Y.; Williams R. S.; Liu Y.; Sun C.; Zhang X.; Kim E.; Shen Y. R.; Fang N. X. Midinfrared Metamaterials Fabricated by Nanoimprint Lithography. Appl. Phys. Lett. 2007, 90, 063107 10.1063/1.2450651.

[ref131] Ok J. G.; Seok Youn H.; Kyu Kwak M.; Lee K.-T.; Jae Shin Y.; Jay Guo L.; Greenwald A.; Liu Y. Continuous and Scalable Fabrication of Flexible Metamaterial Films Via Roll-to-Roll Nanoimprint Process for Broadband Plasmonic Infrared Filters. Appl. Phys. Lett. 2012, 101, 223102 10.1063/1.4767995.

[ref132] Varghese L. T.; Fan L.; Xuan Y.; Tansarawiput C.; Kim S.; Qi M. Resistless Nanoimprinting in Metal for Plasmonic Nanostructures. Small 2013, 9, 3778–3783. 10.1002/smll.201300168. 23606576

[ref133] Chou S. Y.; Krauss P. R.; Renstrom P. J. Imprint Lithography with 25-Nanometer Resolution. Science 1996, 272, 85–87. 10.1126/science.272.5258.85.

[ref134] Lucas B. D.; Kim J.-S.; Chin C.; Guo L. J. Nanoimprint Lithography Based Approach for the Fabrication of Large-Area, Uniformly-Oriented Plasmonic Arrays. Adv. Mater. 2008, 20, 1129–1134. 10.1002/adma.200700225.

[ref135] Zhou J.; Kaplan A. F.; Chen L.; Guo L. J. Experiment and Theory of the Broadband Absorption by a Tapered Hyperbolic Metamaterial Array. ACS Photon. 2014, 1, 618–624. 10.1021/ph5001007.

[ref136] Oh D. K.; Lee T.; Ko B.; Badloe T.; Ok J. G.; Rho J. Nanoimprint Lithography for High-Throughput Fabrication of Metasurfaces. Front. Optoelectron. 2021, 14, 229–251. 10.1007/s12200-021-1121-8. PMC9743954 36637666

[ref137] Ok J. G.; Ahn S. H.; Kwak M. K.; Guo L. J. Continuous and High-Throughput Nanopatterning Methodologies Based on Mechanical Deformation. J. Mater. Chem. C 2013, 1, 7681–7691. 10.1039/c3tc30908h.

[ref138] Buzzi S.; Robin F.; Callegari V.; Loffler J. F. Metal Direct Nanoimprinting for Photonics. Microelectron. Eng. 2008, 85, 419–424. 10.1016/j.mee.2007.08.001.

[ref139] Buzzi S.; Galli M.; Agio M.; Loffler J. F. Silver High-Aspect-Ratio Micro- and Nanoimprinting for Optical Applications. Appl. Phys. Lett. 2009, 94, 223115 10.1063/1.3142426.

[ref140] Curto A. G.; Volpe G.; Taminiau T. H.; Kreuzer M. P.; Quidant R.; van Hulst N. F. Unidirectional Emission of a Quantum Dot Coupled to a Nanoantenna. Science 2010, 329, 930–933. 10.1126/science.1191922. 20724630

[ref141] Rakovich A.; Albella P.; Maier S. A. Plasmonic Control of Radiative Properties of Semiconductor Quantum Dots Coupled to Plasmonic Ring Cavities. ACS Nano 2015, 9, 2648–2658. 10.1021/nn506433e. 25602764

[ref142] Flauraud V.; Mastrangeli M.; Bernasconi G. D.; Butet J.; Alexander D. T. L.; Shahrabi E.; Martin O. J. F.; Brugger J. Nanoscale Topographical Control of Capillary Assembly of Nanoparticles. Nat. Nanotechnol. 2017, 12, 73–80. 10.1038/nnano.2016.179. 27694849

[ref143] Gupta S. N.; Bitton O.; Neuman T.; Esteban R.; Chuntonov L.; Aizpurua J.; Haran G. Complex Plasmon-Exciton Dynamics Revealed through Quantum Dot Light Emission in a Nanocavity. Nat. Commun. 2021, 12, 1310 10.1038/s41467-021-21539-z. 33637699 PMC7910578

[ref144] Novotny L. Strong Coupling, Energy Splitting, and Level Crossings: A Classical Perspective. Am. J. Phys. 2010, 78, 1199–1202. 10.1119/1.3471177.

[ref145] Limonov M. F.; Rybin M. V.; Poddubny A. N.; Kivshar Y. S. Fano Resonances in Photonics. Nat. Photonics 2017, 11, 543–554. 10.1038/nphoton.2017.142.

[ref146] Törmä P.; Barnes W. L. Strong Coupling between Surface Plasmon Polaritons and Emitters: A Review. Rep. Prog. Phys. 2015, 78, 013901 10.1088/0034-4885/78/1/013901. 25536670

[ref147] Purcell E. M. Spontaneous Emission Probabilities at Radio Frequencies. Phys. Rev. 1946, 69, 681 10.1103/PhysRev.69.674.2.

[ref148] Ford G. W.; Weber W. H. Electromagnetic Interactions of Molecules with Metal Surfaces. Phys. Rep. 1984, 113, 195–287. 10.1016/0370-1573(84)90098-X.

[ref149] Hecht B.; Novotny L. Principles of Nano-Optics; Cambridge University Press: Cambridge, 2012; pp 12–44.

[ref150] Jacob Z.; Kim J. Y.; Naik G. V.; Boltasseva A.; Narimanov E. E.; Shalaev V. M. Engineering Photonic Density of States Using Metamaterials. Appl. Phys. B-Lasers O. 2010, 100, 215–218. 10.1007/s00340-010-4096-5.

[ref151] Noginov M. A.; Li H.; Barnakov Y. A.; Dryden D.; Nataraj G.; Zhu G.; Bonner C. E.; Mayy M.; Jacob Z.; Narimanov E. E. Controlling Spontaneous Emission with Metamaterials. Opt. Lett. 2010, 35, 1863–1865. 10.1364/OL.35.001863. 20517443

[ref152] Tumkur T.; Zhu G.; Black P.; Barnakov Y. A.; Bonner C. E.; Noginov M. A. Control of Spontaneous Emission in a Volume of Functionalized Hyperbolic Metamaterial. Appl. Phys. Lett. 2011, 99, 151115 10.1063/1.3631723.

[ref153] Iorsh I.; Poddubny A.; Orlov A.; Belov P.; Kivshar Y. S. Spontaneous Emission Enhancement in Metal Dielectric Metamaterials. Phys. Lett. A 2012, 376, 185–187. 10.1016/j.physleta.2011.11.001.

[ref154] Kidwai O.; Zhukovsky S. V.; Sipe J. E. Effective-Medium Approach to Planar Multilayer Hyperbolic Metamaterials: Strengths and Limitations. Phys. Rev. A 2012, 85, 053842 10.1103/PhysRevA.85.053842.

[ref155] Kim J.; Drachev V. P.; Jacob Z.; Naik G. V.; Boltasseva A.; Narimanov E. E.; Shalaev V. M. Improving the Radiative Decay Rate for Dye Molecules with Hyperbolic Metamaterials. Opt. Express 2012, 20, 8100–8116. 10.1364/OE.20.008100. 22453481

[ref156] Krishnamoorthy H. N. S.; Jacob Z.; Narimanov E.; Kretzschmar I.; Menon V. M. Topological Transitions in Metamaterials. Science 2012, 336, 205–209. 10.1126/science.1219171. 22499943

[ref157] Ferrari L.; Lu D. L.; Lepage D.; Liu Z. W. Enhanced Spontaneous Emission inside Hyperbolic Metamaterials. Opt. Express 2014, 22, 4301–4306. 10.1364/OE.22.004301. 24663753

[ref158] Guclu C.; Luk T. S.; Wang G. T.; Capolino F. Radiative Emission Enhancement Using Nano-Antennas Made of Hyperbolic Metamaterial Resonators. Appl. Phys. Lett. 2014, 105, 123101 10.1063/1.4895816.

[ref159] Podolskiy V. A.; Ginzburg P.; Wells B.; Zayats A. V. Light Emission in Nonlocal Plasmonic Metamaterials. Faraday Discuss. 2015, 178, 61–70. 10.1039/C4FD00186A. 25728217

[ref160] Slobozhanyuk A. P.; Ginzburg P.; Powell D. A.; Iorsh I.; Shalin A. S.; Segovia P.; Krasavin A. V.; Wurtz G. A.; Podolskiy V. A.; Belov P. A.; et al. Purcell Effect in Hyperbolic Metamaterial Resonators. Phys. Rev. B 2015, 92, 195127 10.1103/PhysRevB.92.195127.

[ref161] Roth D.; Ginzburg P.; Hirvonen L. M.; Levitt J. A.; Nasir M. E.; Suhling K.; Richards D.; Podolskiy V. A.; Zayats A. V. Singlet-Triplet Transition Rate Enhancement inside Hyperbolic Metamaterials. Laser Photonics Rev. 2019, 13, 1900101 10.1002/lpor.201900101.

[ref162] Li J.; Krasavin A. V.; Webster L.; Segovia P.; Zayats A. V.; Richards D. Spectral Variation of Fluorescence Lifetime near Single Metal Nanoparticles. Sci. Rep. 2016, 6, 21349 10.1038/srep21349. 26876780 PMC4753420

[ref163] Sreekanth K. V.; Krishna K. H.; De Luca A.; Strangi G. Large Spontaneous Emission Rate Enhancement in Grating Coupled Hyperbolic Metamaterials. Sci. Rep. 2015, 4, 6340 10.1038/srep06340. PMC4160708 25209102

[ref164] Galfsky T.; Krishnamoorthy H. N. S.; Newman W.; Narimanov E. E.; Jacob Z.; Menon V. M. Active Hyperbolic Metamaterials: Enhanced Spontaneous Emission and Light Extraction. Optica 2015, 2, 62–65. 10.1364/OPTICA.2.000062.

[ref165] Inam F. A.; Ahmed N.; Steel M. J.; Castelletto S. Hyperbolic Metamaterial Resonator-Antenna Scheme for Large, Broadband Emission Enhancement and Single-Photon Collection. J. Opt. Soc. Am. B 2018, 35, 2153–2162. 10.1364/JOSAB.35.002153.

[ref166] Wang W.; Yang X. D.; Gao J. Scaling Law of Purcell Factor in Hyperbolic Metamaterial Cavities with Dipole Excitation. Opt. Lett. 2019, 44, 471–474. 10.1364/OL.44.000471. 30702656

[ref167] Tumkur T. U.; Kitur J. K.; Bonner C. E.; Poddubny A. N.; Narimanov E. E.; Noginov M. A. Control of Förster Energy Transfer in the Vicinity of Metallic Surfaces and Hyperbolic Metamaterials. Faraday Discuss. 2015, 178, 395–412. 10.1039/C4FD00184B. 25803206

[ref168] Roth D. J.; Nasir M. E.; Ginzburg P.; Wang P.; Le Marois A.; Suhling K.; Richards D.; Zayats A. V. Forster Resonance Energy Transfer inside Hyperbolic Metamaterials. ACS Photonics 2018, 5, 4594–4603. 10.1021/acsphotonics.8b01083.

[ref169] Weis P.; Garcia-Pomar J. L.; Beigang R.; Rahm M. Hybridization Induced Transparency in Composites of Metamaterials and Atomic Media. Opt. Express 2011, 19, 23573–23580. 10.1364/OE.19.023573. 22109237

[ref170] Tanyi E. K.; Hong N.; Sawyer T.; Van Schenck J. D. B.; Giesbers G.; Ostroverkhova O.; Cheng L. J. Strong Exciton-Plasmon Coupling in Dye-Doped Film on a Planar Hyperbolic Metamaterial. Opt. Lett. 2020, 45, 6736–6739. 10.1364/OL.402210. 33325884

[ref171] Mahmud M. S.; Rosenmann D.; Czaplewski D. A.; Gao J.; Yang X. D. Plasmon-Phonon Coupling between Mid-Infrared Chiral Metasurfaces and Molecular Vibrations. Opt. Express 2020, 28, 21192–21201. 10.1364/OE.397725. 32680164

[ref172] Nishijima Y.; Morimoto S.; Balcytis A.; Hashizume T.; Matsubara R.; Kubono A.; To N.; Ryu M.; Morikawa J.; Juodkazis S. Coupling of Molecular Vibration and Metasurface Modes for Efficient Mid-Infrared Emission. J. Mater. Chem. C 2022, 10, 451 10.1039/D1TC04519A.

[ref173] Ginzburg P.; Krasavin A. V.; Richards D.; Zayats A. V. Impact of Nonradiative Line Broadening on Emission in Photonic and Plasmonic Cavities. Phys. Rev. A 2014, 90, 043836 10.1103/PhysRevA.90.043836.

[ref174] Huang Z. X.; Droulias S.; Koschny T.; Soukoulis C. M. Mechanism of the Metallic Metamaterials Coupled to the Gain Material. Opt. Express 2014, 22, 28596–28605. 10.1364/OE.22.028596. 25402101

[ref175] Ramezani M.; Halpin A.; Fernandez-Dominguez A. I.; Feist J.; Rodriguez S. R. K.; Garcia-Vidal F. J.; Rivas J. G. Plasmon-Exciton-Polariton Lasing. Optica 2017, 4, 31–37. 10.1364/OPTICA.4.000031.

[ref176] Shi L.; Hakala T. K.; Rekola H. T.; Martikainen J. P.; Moerland R. J.; Torma P. Spatial Coherence Properties of Organic Molecules Coupled to Plasmonic Surface Lattice Resonances in the Weak and Strong Coupling Regimes. Phys. Rev. Lett. 2014, 112, 153002 10.1103/PhysRevLett.112.153002. 24785036

[ref177] Schwartz T.; Hutchison J. A.; Genet C.; Ebbesen T. W. Reversible Switching of Ultrastrong Light-Molecule Coupling. Phys. Rev. Lett. 2011, 106, 196405 10.1103/PhysRevLett.106.196405. 21668181

[ref178] Yoo D.; de Leon-Perez F.; Pelton M.; Lee I. H.; Mohr D. A.; Raschke M. B.; Caldwell J. D.; Martin-Moreno L.; Oh S. H. Ultrastrong Plasmon-Phonon Coupling Via Epsilon-near-Zero Nanocavities. Nat. Photonics 2021, 15, 125–130. 10.1038/s41566-020-00731-5.

[ref179] Wu F.; Guo J.; Huang Y.; Liang K.; Jin L.; Li J.; Deng X.; Jiao R.; Liu Y.; Zhang J.; et al. Plexcitonic Optical Chirality: Strong Exciton–Plasmon Coupling in Chiral J-Aggregate-Metal Nanoparticle Complexes. ACS Nano 2021, 15, 2292–2300. 10.1021/acsnano.0c08274. 33356158

[ref180] Orgiu E.; George J.; Hutchison J. A.; Devaux E.; Dayen J. F.; Doudin B.; Stellacci F.; Genet C.; Schachenmayer J.; Genes C.; et al. Conductivity in Organic Semiconductors Hybridized with the Vacuum Field. Nat. Mater. 2015, 14, 1123–1129. 10.1038/nmat4392. 26366850

[ref181] Li C.; Lu X.; Srivastava A.; Storm S. D.; Gelfand R.; Pelton M.; Sukharev M.; Harutyunyan H. Second Harmonic Generation from a Single Plasmonic Nanorod Strongly Coupled to a Wse2Monolayer. Nano Lett. 2021, 21, 1599–1605. 10.1021/acs.nanolett.0c03757. 33306403

[ref182] Fofang N. T.; Grady N. K.; Fan Z.; Govorov A. O.; Halas N. J. Plexciton Dynamics: Exciton–Plasmon Coupling in a J-Aggregate–Au Nanoshell Complex Provides a Mechanism for Nonlinearity. Nano Lett. 2011, 11, 1556–1560. 10.1021/nl104352j. 21417362

[ref183] Thomas A.; George J.; Shalabney A.; Dryzhakov M.; Varma S. J.; Moran J.; Chervy T.; Zhong X. L.; Devaux E.; Genet C.; et al. Ground-State Chemical Reactivity under Vibrational Coupling to the Vacuum Electromagnetic Field. Angew. Chem., Int. Ed. Engl. 2016, 55, 11462–11466. 10.1002/anie.201605504. 27529831 PMC5113700

[ref184] Dovzhenko D. S.; Ryabchuk S. V.; Rakovich Y. P.; Nabiev I. R. Light–Matter Interaction in the Strong Coupling Regime: Configurations, Conditions, and Applications. Nanoscale 2018, 10, 3589–3605. 10.1039/C7NR06917K. 29419830

[ref185] Wurtz G. A.; Evans P. R.; Hendren W.; Atkinson R.; Dickson W.; Pollard R. J.; Zayats A. V.; Harrison W.; Bower C. Molecular Plasmonics with Tunable Exciton–Plasmon Coupling Strength in J-Aggregate Hybridized Au Nanorod Assemblies. Nano Lett. 2007, 7, 1297–1303. 10.1021/nl070284m. 17455984

[ref186] Liu N.; Fu L.; Kaiser S.; Schweizer H.; Giessen H. Plasmonic Building Blocks for Magnetic Molecules in Three-Dimensional Optical Metamaterials. Adv. Mater. 2008, 20, 3859–3865. 10.1002/adma.200702950.

[ref187] Valentine J.; Zhang S.; Zentgraf T.; Ulin-Avila E.; Genov D. A.; Bartal G.; Zhang X. Three-Dimensional Optical Metamaterial with a Negative Refractive Index. Nature 2008, 455, 376–379. 10.1038/nature07247. 18690249

[ref188] Chanda D.; Shigeta K.; Gupta S.; Cain T.; Carlson A.; Mihi A.; Baca A. J.; Bogart G. R.; Braun P.; Rogers J. A. Large-Area Flexible 3D Optical Negative Index Metamaterial Formed by Nanotransfer Printing. Nat. Nanotechnol. 2011, 6, 402–407. 10.1038/nnano.2011.82. 21642984

[ref189] Stockman M. I. Loss Compensation by Gain and Spasing. Philos. Trans. R. Soc. A 2011, 369, 3510–3524. 10.1098/rsta.2011.0143. 21807725

[ref190] Sivan Y.; Xiao S. M.; Chettiar U. K.; Kildishev A. V.; Shalaev V. M. Frequency-Domain Simulations of a Negative-Index Material with Embedded Gain. Opt. Express 2009, 17, 24060–24074. 10.1364/OE.17.024060. 20052118

[ref191] Wuestner S.; Pusch A.; Tsakmakidis K. L.; Hamm J. M.; Hess O. Overcoming Losses with Gain in a Negative Refractive Index Metamaterial. Phys. Rev. Lett. 2010, 105, 127401 10.1103/PhysRevLett.105.127401. 20867669

[ref192] Hamm J. M.; Wuestner S.; Tsakmakidis K. L.; Hess O. Theory of Light Amplification in Active Fishnet Metamaterials. Phys. Rev. Lett. 2011, 107, 167405 10.1103/PhysRevLett.107.167405. 22107428

[ref193] Wuestner S.; Pusch A.; Tsakmakidis K. L.; Hamm J. M.; Hess O. Gain and Plasmon Dynamics in Active Negative-Index Metamaterials. Philos. Trans. R. Soc. A 2011, 369, 3525–3550. 10.1098/rsta.2011.0140. 21807726

[ref194] Pusch A.; Wuestner S.; Hamm J. M.; Tsakmakidis K. L.; Hess O. Coherent Amplification and Noise in Gain-Enhanced Nanoplasmonic Metamaterials: A Maxwell-Bloch Langevin Approach. ACS Nano 2012, 6, 2420–2431. 10.1021/nn204692x. 22329714

[ref195] Wuestner S.; Hamm J. M.; Pusch A.; Renn F.; Tsakmakidis K. L.; Hess O. Control and Dynamic Competition of Bright and Dark Lasing States in Active Nanoplasmonic Metamaterials. Phys. Rev. B 2012, 85, 201406 10.1103/PhysRevB.85.201406.

[ref196] Wuestner S.; Pusch A.; Hamm J. M.; Tsakmakidis K. L.; Hess O. Dynamics of Amplification in a Nanoplasmonic Metamaterial. Appl. Phys. A: Mater. Sci. Process. 2012, 107, 77–82. 10.1007/s00339-012-6784-y.

[ref197] Xiao S. M.; Drachev V. P.; Kildishev A. V.; Ni X. J.; Chettiar U. K.; Yuan H. K.; Shalaev V. M. Loss-Free and Active Optical Negative-Index Metamaterials. Nature 2010, 466, 735–U736. 10.1038/nature09278. 20686570

[ref198] Fang M.; Huang Z. X.; Sha W. E. I.; Soukoulis C. M. Modelling of the Fluctuation and Coherent Dynamics in Active Metamaterial Devices. IEEE Trans. Nanotechnol. 2021, 20, 543–551. 10.1109/TNANO.2021.3092059.

[ref199] Wegener M.; Garcia-Pomar J. L.; Soukoulis C. M.; Meinzer N.; Ruther M.; Linden S. Toy Model for Plasmonic Metamaterial Resonances Coupled to Two-Level System Gain. Opt. Express 2008, 16, 19785–19798. 10.1364/OE.16.019785. 19030064

[ref200] Droulias S.; Koschny T.; Kafesaki M.; Soukoulis C. M. On Loss Compensation, Amplification and Lasing in Metallic Metamaterials. Nanomater. Nanotechnol. 2019, 9, 184798041881794 10.1177/1847980418817947.

[ref201] Zheludev N. I.; Prosvirnin S. L.; Papasimakis N.; Fedotov V. A. Lasing Spaser. Nat. Photonics 2008, 2, 351–354. 10.1038/nphoton.2008.82.

[ref202] Huang Y. W.; Chen W. T.; Wu P. C.; Fedotov V. A.; Zheludev N. I.; Tsai D. P. Toroidal Lasing Spaser. Sci. Rep. 2013, 3, 1237 10.1038/srep01237. 23393619 PMC3566619

[ref203] Fang A.; Koschny T.; Soukoulis C. M. Lasing in Metamaterial Nanostructures. J. Opt. 2010, 12, 024013 10.1088/2040-8978/12/2/024013.

[ref204] Azzam S. I.; Kildishev A. V.; Ma R.-M.; Ning C.-Z.; Oulton R.; Shalaev V. M.; Stockman M. I.; Xu J.-L.; Zhang X. Ten Years of Spasers and Plasmonic Nanolasers. Light Sci. Appl. 2020, 9, 90 10.1038/s41377-020-0319-7. 32509297 PMC7248101

[ref205] Kitur J. K.; Gu L.; Tumkur T.; Bonner C.; Noginov M. A. Stimulated Emission of Surface Plasmons on Top of Metamaterials with Hyperbolic Dispersion. ACS Photonics 2015, 2, 1019–1024. 10.1021/ph500475x.

[ref206] Chandrasekar R.; Wang Z. X.; Meng X. G.; Azzam S. I.; Shalaginov M. Y.; Lagutchev A.; Kim Y. L.; Wei A.; Kildishev A. V.; Boltasseva A.; et al. Lasing Action with Gold Nanorod Hyperbolic Metamaterials. ACS Photonics 2017, 4, 674–680. 10.1021/acsphotonics.7b00010.

[ref207] Pustovit V. N.; Urbas A. M.; Zelmon D. E. Surface Plasmon Amplification by Stimulated Emission of Radiation in Hyperbolic Metamaterials. Phys. Rev. B 2016, 94, 235445 10.1103/PhysRevB.94.235445

[ref208] Shramkova O. V.; Tsironis G. P. Propagation of Electromagnetic Waves in PT-Symmetric Hyperbolic Structures. Phys. Rev. B 2016, 94, 035141 10.1103/PhysRevB.94.035141

[ref209] Janaszek B.; Szczepanski P. Distributed Feedback Laser Based on Tunable Photonic Hypercrystal. Materials 2021, 14, 4065 10.3390/ma14154065 34361259 PMC8348560

[ref210] Shen K. C.; Ku C. T.; Hsieh C.; Kuo H. C.; Cheng Y. J.; Tsai D. P. Deep-Ultraviolet Hyperbolic Metacavity Laser. Adv. Mater. 2018, 30, 1706918 10.1002/adma.201706918 29633385

[ref211] Haider G.; Lin H. I.; Yadav K.; Shen K. C.; Liao Y. M.; Hu H. W.; Roy P. K.; Bera K. P.; Lin K. H.; Lee H. M.; et al. A Highly-Efficient Single Segment White Random Laser. ACS Nano 2018, 12, 11847–11859. 10.1021/acsnano.8b03035. 30352157

[ref212] Caligiuri V.; Pezzi L.; Veltri A.; De Luca A. Resonant Gain Singularities in 1D and 3D Metal/Dielectric Multilayered Nanostructures. ACS Nano 2017, 11, 1012–1025. 10.1021/acsnano.6b07638. 28009498

[ref213] Ni X. J.; Ishii S.; Thoreson M. D.; Shalaev V. M.; Han S. H.; Lee S.; Kildishev A. V. Loss-Compensated and Active Hyperbolic Metamaterials. Opt. Express 2011, 19, 25242–25254. 10.1364/OE.19.025242. 22273915

[ref214] Argyropoulos C.; Estakhri N. M.; Monticone F.; Alu A. Negative Refraction, Gain and Nonlinear Effects in Hyperbolic Metamaterials. Opt. Express 2013, 21, 15037–15047. 10.1364/OE.21.015037. 23787691

[ref215] Caligiuri V.; Dhama R.; Sreekanth K. V.; Strangi G.; De Luca A. Dielectric Singularity in Hyperbolic Metamaterials: The Inversion Point of Coexisting Anisotropies. Sci. Rep. 2016, 6, 20002 10.1038/srep20002. 26833022 PMC4735793

[ref216] Vecchi G.; Giannini V.; Gómez Rivas J. Shaping the Fluorescent Emission by Lattice Resonances in Plasmonic Crystals of Nanoantennas. Phys. Rev. Lett. 2009, 102, 146807 10.1103/PhysRevLett.102.146807. 19392471

[ref217] Lozano G.; Louwers D. J; Rodríguez S. R.; Murai S.; Jansen O. T.; Verschuuren M. A; Gómez Rivas J. Plasmonics for Solid-State Lighting: Enhanced Excitation and Directional Emission of Highly Efficient Light Sources. Light Sci. Appl. 2013, 2, e66 10.1038/lsa.2013.22.

[ref218] Murai S.; Verschuuren M. A.; Lozano G.; Pirruccio G.; Rodriguez S. R. K.; Rivas J. G. Hybrid Plasmonic-Photonic Modes in Diffractive Arrays of Nanoparticles Coupled to Light-Emitting Optical Waveguides. Opt. Express 2013, 21, 4250–4262. 10.1364/OE.21.004250. 23481959

[ref219] Lozano G.; Grzela G.; Verschuuren M. A.; Ramezani M.; Rivas J. G. Tailor-Made Directional Emission in Nanoimprinted Plasmonic-Based Light-Emitting Devices. Nanoscale 2014, 6, 9223–9229. 10.1039/C4NR01391C. 24981706

[ref220] Nikitin A.; Remezani M.; Rivas J. G. Luminescent Metamaterials for Solid State Lighting. ECS J. Solid State Sci. Technol. 2016, 5, R3164–R3169. 10.1149/2.0211601jss.

[ref221] Zhou W.; Dridi M.; Suh J. Y.; Kim C. H.; Co D. T.; Wasielewski M. R.; Schatz G. C.; Odom T. W. Lasing Action in Strongly Coupled Plasmonic Nanocavity Arrays. Nat. Nanotechnol. 2013, 8, 506–511. 10.1038/nnano.2013.99. 23770807

[ref222] Stehr J.; Crewett J.; Schindler F.; Sperling R.; von Plessen G.; Lemmer U.; Lupton J. M.; Klar T. A.; Feldmann J.; Holleitner A. W.; et al. A Low Threshold Polymer Laser Based on Metallic Nanoparticle Gratings. Adv. Mater. 2003, 15, 1726 10.1002/adma.200305221.

[ref223] Schokker A. H.; Koenderink A. F. Lasing at the Band Edges of Plasmonic Lattices. Phys. Rev. B 2014, 90, 155452 10.1103/PhysRevB.90.155452.

[ref224] Schokker A. H.; Koenderink A. F. Statistics of Randomized Plasmonic Lattice Lasers. ACS Photonics 2015, 2, 1289–1297. 10.1021/acsphotonics.5b00226.

[ref225] Yang A. K.; Li Z. Y.; Knudson M. P.; Hryn A. J.; Wang W. J.; Aydin K.; Odom T. W. Unidirectional Lasing from Template-Stripped Two-Dimensional Plasmonic Crystals. ACS Nano 2015, 9, 11582–11588. 10.1021/acsnano.5b05419. 26456299

[ref226] Yang A.; Hoang T. B.; Dridi M.; Deeb C.; Mikkelsen M. H.; Schatz G. C.; Odom T. W. Real-Time Tunable Lasing from Plasmonic Nanocavity Arrays. Nat. Commun. 2015, 6, 6939 10.1038/ncomms7939. 25891212 PMC4411284

[ref227] Schokker A. H.; Koenderink A. F. Lasing in Quasi-Periodic and Aperiodic Plasmon Lattices. Optica 2016, 3, 686–693. 10.1364/OPTICA.3.000686.

[ref228] Wang D. Q.; Yang A. K.; Wang W. J.; Hua Y.; Schaller R. D.; Schatz G. C.; Odom T. W. Band-Edge Engineering for Controlled Multi-Modal Nanolasing in Plasmonic Superlattices. Nat. Nanotechnol. 2017, 12, 889 10.1038/nnano.2017.126. 28692060

[ref229] Hakala T. K.; Rekola H. T.; Vakevainen A. I.; Martikainen J. P.; Necada M.; Moilanen A. J.; Torma P. Lasing in Dark and Bright Modes of a Finite-Sized Plasmonic Lattice. Nat. Commun. 2017, 8, 13687 10.1038/ncomms13687. 28045047 PMC5216126

[ref230] Schokker A. H.; van Riggelen F.; Hadad Y.; Alu A.; Koenderink A. F. Systematic Study of the Hybrid Plasmonic-Photonic Band Structure Underlying Lasing Action of Diffractive Plasmon Particle Lattices. Phys. Rev. B 2017, 95, 085409 10.1103/PhysRevB.95.085409.

[ref231] Hoang T. B.; Akselrod G. M.; Yang A. K.; Odom T. W.; Mikkelsen M. H. Millimeter-Scale Spatial Coherence from a Plasmon Laser. Nano Lett. 2017, 17, 6690–6695. 10.1021/acs.nanolett.7b02677. 28956442

[ref232] Wang Z. X.; Meng X. G.; Choi S. H.; Knitter S.; Kim Y. L.; Cao H.; Shalaev V. M.; Boltasseva A. Controlling Random Lasing with Three-Dimensional Plasmonic Nanorod Metamaterials. Nano Lett. 2016, 16, 2471–2477. 10.1021/acs.nanolett.6b00034. 27023052

[ref233] Kumar R.; Tiwari A. K.; Anantha Ramakrishna S. Surface Plasmon Coupling for Selectively Enhanced Random Lasing in Periodically Patterned Silver Columnar Thin Film Metamaterials. Appl. Phys. Lett. 2020, 116, 241902 10.1063/5.0010413.

[ref234] Zheludev N. I.; Plum E. Reconfigurable Nanomechanical Photonic Metamaterials. Nat. Nanotechnol. 2016, 11, 16–22. 10.1038/nnano.2015.302. 26740040

[ref235] Ee H. S.; Agarwal R. Tunable Metasurface and Flat Optical Zoom Lens on a Stretchable Substrate. Nano Lett. 2016, 16, 2818–2823. 10.1021/acs.nanolett.6b00618. 26986191

[ref236] Malek S. C.; Ee H. S.; Agarwal R. Strain Multiplexed Metasurface Holograms on a Stretchable Substrate. Nano Lett. 2017, 17, 3641–3645. 10.1021/acs.nanolett.7b00807. 28488437

[ref237] Ding T.; Rüttiger C.; Zheng X.; Benz F.; Ohadi H.; Vandenbosch G. A. E.; Moshchalkov V. V.; Gallei M.; Baumberg J. J. Fast Dynamic Color Switching in Temperature-Responsive Plasmonic Films. Adv. Opt. Mater. 2016, 4, 877–882. 10.1002/adom.201600094.

[ref238] Wang Q. G.; Liu L. J.; Wang Y. F.; Liu P.; Jiang H. W.; Xu Z.; Ma Z.; Oren S.; Chow E. K. C.; Lu M.; et al. Tunable Optical Nanoantennas Incorporating Bowtie Nanoantenna Arrays with Stimuli-Responsive Polymer. Sci. Rep. 2016, 5, 18567 10.1038/srep18567. PMC4683518 26681478

[ref239] Gehan H.; Mangeney C.; Aubard J.; Levi G.; Hohenau A.; Krenn J. R.; Lacaze E.; Felidj N. Design and Optical Properties of Active Polymer-Coated Plasmonic Nanostructures. J. Phys. Chem. Lett. 2011, 2, 926–931. 10.1021/jz200272r. 26295630

[ref240] Zhang Y. C.; Li Y.; Hu Y. L.; Zhu X. L.; Huang Y. W.; Zhang Z.; Rao S. L.; Hu Z. J.; Qiu W. X.; Wang Y. L.; et al. Localized Self- Growth of Reconfigurable Architectures Induced by a Femtosecond Laser on a Shape-Memory Polymer. Adv. Mater. 2018, 30, 1803072 10.1002/adma.201803072. 30259576

[ref241] Jiang S. J.; Hu Y. L.; Wu H.; Zhang Y. C.; Zhang Y. Y.; Wang Y. L.; Zhang Y. H.; Zhu W. L.; Li J. W.; Wu D.; et al. Multifunctional Janus Microplates Arrays Actuated by Magnetic Fields for Water/Light Switches and Bio-Inspired Assimilatory Coloration. Adv. Mater. 2019, 31, 1807507 10.1002/adma.201807507. 30721548

[ref242] Lin Q.-Y.; Mason J. A.; Li Z.; Zhou W.; O’Brien M. N.; Brown K. A.; Jones M. R.; Butun S.; Lee B.; Dravid V. P.; Aydin K.; Mirkin C. A.; et al. Building Superlattices from Individual Nanoparticles Via Template-Confined DNA-Mediated Assembly. Science 2018, 359, 669–672. 10.1126/science.aaq0591. 29348364

[ref243] Litt D. B.; Jones M. R.; Hentschel M.; Wang Y.; Yang S.; Ha H. D.; Zhang X.; Alivisatos A. P. Hybrid Lithographic and DNA-Directed Assembly of a Configurable Plasmonic Metamaterial That Exhibits Electromagnetically Induced Transparency. Nano Lett. 2018, 18, 859–864. 10.1021/acs.nanolett.7b04116. 29303595

[ref244] Kuzyk A.; Schreiber R.; Zhang H.; Govorov A. O.; Liedl T.; Liu N. Reconfigurable 3D Plasmonic Metamolecules. Nat. Mater. 2014, 13, 862–866. 10.1038/nmat4031. 24997737

[ref245] Umadevi S.; Feng X.; Hegmann T. Large Area Self-Assembly of Nematic Liquid-Crystal-Functionalized Gold Nanorods. Adv. Funct. Mater. 2013, 23, 1393–1403. 10.1002/adfm.201202727.

[ref246] Urban M. J.; Zhou C.; Duan X.; Liu N. Optically Resolving the Dynamic Walking of a Plasmonic Walker Couple. Nano Lett. 2015, 15, 8392–8396. 10.1021/acs.nanolett.5b04270. 26571209

[ref247] Zhou C.; Duan X. Y.; Liu N. A Plasmonic Nanorod That Walks on DNA Origami. Nat. Commun. 2015, 6,8102 10.1038/ncomms9102. 26303016 PMC4560816

[ref248] Kuzyk A.; Yang Y.; Duan X.; Stoll S.; Govorov A. O.; Sugiyama H.; Endo M.; Liu N. A Light-Driven Three-Dimensional Plasmonic Nanosystem That Translates Molecular Motion into Reversible Chiroptical Function. Nat. Commun. 2016, 7, 10591 10.1038/ncomms10591. 26830310 PMC4740900

[ref249] Di Martino G.; Tappertzhofen S.; Hofmann S.; Baumberg J. Nanoscale Plasmon-Enhanced Spectroscopy in Memristive Switches. Small 2016, 12, 1334–1341. 10.1002/smll.201503165. 26756792

[ref250] Schoen D. T.; Holsteen A. L.; Brongersma M. L. Probing the Electrical Switching of a Memristive Optical Antenna by STEM EELS. Nat. Commun. 2016, 7, 12162 10.1038/ncomms12162. 27412052 PMC4947179

[ref251] Thyagarajan K.; Sokhoyan R.; Zornberg L.; Atwater H. A. Millivolt Modulation of Plasmonic Metasurface Optical Response Via Ionic Conductance. Adv. Mater. 2017, 29, 1701044 10.1002/adma.201701044. 28612946

[ref252] Wang G.; Chen X.; Liu S.; Wong C.; Chu S. Mechanical Chameleon through Dynamic Real-Time Plasmonic Tuning. ACS Nano 2016, 10, 1788–1794. 10.1021/acsnano.5b07472. 26760215

[ref253] Liu Q.; Cui Y.; Gardner D.; Li X.; He S.; Smalyukh I. I. Self-Alignment of Plasmonic Gold Nanorods in Reconfigurable Anisotropic Fluids for Tunable Bulk Metamaterial Applications. Nano Lett. 2010, 10, 1347–1353. 10.1021/nl9042104. 20334353

[ref254] Liu Q.; Yuan Y.; Smalyukh I. I. Electrically and Optically Tunable Plasmonic Guest–Host Liquid Crystals with Long-Range Ordered Nanoparticles. Nano Lett. 2014, 14, 4071–4077. 10.1021/nl501581y. 24884975

[ref255] Thomas M. R.; Klein S.; Greasty R. J.; Mann S.; Perriman A. W.; Richardson R. M. Nematic Director-Induced Switching of Assemblies of Hexagonally Packed Gold Nanorods. Adv. Mater. 2012, 24, 4424–4429. 10.1002/adma.201201319. 22761047

[ref256] Zhang Y.; Liu Q.; Mundoor H.; Yuan Y.; Smalyukh I. I. Metal Nanoparticle Dispersion, Alignment, and Assembly in Nematic Liquid Crystals for Applications in Switchable Plasmonic Color Filters and E-Polarizers. ACS Nano 2015, 9, 3097–3108. 10.1021/nn5074644. 25712232

[ref257] Boehm S. J.; Kang L.; Werner D. H.; Keating C. D. Field-Switchable Broadband Polarizer Based on Reconfigurable Nanowire Assemblies. Adv. Funct. Mater. 2017, 27, 1604703 10.1002/adfm.201604703.

[ref258] Sterl F.; Strohfeldt N.; Walter R.; Griessen R.; Tittl A.; Giessen H. Magnesium as Novel Material for Active Plasmonics in the Visible Wavelength Range. Nano Lett. 2015, 15, 7949–7955. 10.1021/acs.nanolett.5b03029. 26312401

[ref259] Duan X.; Kamin S.; Sterl F.; Giessen H.; Liu N. Hydrogen-Regulated Chiral Nanoplasmonics. Nano Lett. 2016, 16, 1462–1466. 10.1021/acs.nanolett.5b05105. 26745446

[ref260] Duan X.; Kamin S.; Liu N. Dynamic Plasmonic Colour Display. Nat. Commun. 2017, 8, 14606 10.1038/ncomms14606. 28232722 PMC5333121

[ref261] Yu P.; Li J.; Zhang S.; Jin Z.; Schütz G.; Qiu C.-W.; Hirscher M.; Liu N. Dynamic Janus Metasurfaces in the Visible Spectral Region. Nano Lett. 2018, 18, 4584–4589. 10.1021/acs.nanolett.8b01848. 29927600

[ref262] Byers C. P.; Zhang H.; Swearer D. F.; Yorulmaz M.; Hoener B. S.; Huang D.; Hoggard A.; Chang W.-S.; Mulvaney P.; Ringe E.; Halas N. J.; Nordlander P.; Link S.; Landes C. F.; et al. From Tunable Core-Shell Nanoparticles to Plasmonic Drawbridges: Active Control of Nanoparticle Optical Properties. Sci. Adv. 2015, 1, e1500988 10.1126/sciadv.1500988. 26665175 PMC4672758

[ref263] Wu Y.; Yang W.; Fan Y.; Song Q.; Xiao S. TiO_2_ Metasurfaces: From Visible Planar Photonics to Photochemistry. Sci. Adv. 2019, 5, eaax0939 10.1126/sciadv.aax0939. 31701001 PMC6824849

[ref264] Li Y.; van de Groep J.; Talin A. A.; Brongersma M. L. Dynamic Tuning of Gap Plasmon Resonances Using a Solid-State Electrochromic Device. Nano Lett. 2019, 19, 7988–7995. 10.1021/acs.nanolett.9b03143. 31560552

[ref265] Peng J.; Jeong H.-H.; Lin Q.; Cormier S.; Liang H.-L.; De Volder M. F. L.; Vignolini S.; Baumberg J. J. Scalable Electrochromic Nanopixels Using Plasmonics. Sci. Adv. 2019, 5, eaaw2205 10.1126/sciadv.aaw2205. 31093530 PMC6510554

[ref266] Ren M.-X.; Wu W.; Cai W.; Pi B.; Zhang X.-Z.; Xu J.-J. Reconfigurable Metasurfaces That Enable Light Polarization Control by Light. Light Sci. Appl. 2017, 6, e16254–e16254. 10.1038/lsa.2016.254. 30167257 PMC6062238

[ref267] Leroux Y. R.; Lacroix J. C.; Chane-Ching K. I.; Fave C.; Félidj N.; Lévi G.; Aubard J.; Krenn J. R.; Hohenau A. Conducting Polymer Electrochemical Switching as an Easy Means for Designing Active Plasmonic Devices. J. Am. Chem. Soc. 2005, 127, 16022–16023. 10.1021/ja054915v. 16287278

[ref268] Leroux Y.; Lacroix J. C.; Fave C.; Trippe G.; Félidj N.; Aubard J.; Hohenau A.; Krenn J. R. Tunable Electrochemical Switch of the Optical Properties of Metallic Nanoparticles. ACS Nano 2008, 2, 728–732. 10.1021/nn700438a. 19206604

[ref269] Xu T.; Walter E. C.; Agrawal A.; Bohn C.; Velmurugan J.; Zhu W.; Lezec H. J.; Talin A. A. High-Contrast and Fast Electrochromic Switching Enabled by Plasmonics. Nat. Commun. 2016, 7, 10479 10.1038/ncomms10479. 26814453 PMC4737852

[ref270] Lu W.; Jiang N.; Wang J. Active Electrochemical Plasmonic Switching on Polyaniline-Coated Gold Nanocrystals. Adv. Mater. 2017, 29, 1604862 10.1002/adma.201604862. 28004862

[ref271] Karki A.; Cincotti G.; Chen S.; Stanishev V.; Darakchieva V.; Wang C.; Fahlman M.; Jonsson M. P. Electrical Tuning of Plasmonic Conducting Polymer Nanoantennas. Adv. Mater. 2022, 34, 2107172 10.1002/adma.202107172. 35064601

[ref272] Stockhausen V.; Martin P.; Ghilane J.; Leroux Y.; Randriamahazaka H.; Grand J.; Felidj N.; Lacroix J. C. Giant Plasmon Resonance Shift Using Poly(3,4-Ethylenedioxythiophene) Electrochemical Switching. J. Am. Chem. Soc. 2010, 132, 10224–10226. 10.1021/ja103337d. 20662496

[ref273] Chen S.; Kang E. S. H.; Shiran Chaharsoughi M.; Stanishev V.; Kühne P.; Sun H.; Wang C.; Fahlman M.; Fabiano S.; Darakchieva V.; et al. Conditions for Fabrication of Ideally Ordered Anodic Porous Alumina Using Pretextured Al. Nat. Nanotechnol. 2020, 15, 35–40. 10.1038/s41565-019-0583-y. 31819242

[ref274] Rossi S.; Olsson O.; Chen S.; Shanker R.; Banerjee D.; Dahlin A.; Jonsson M. P. Dynamically Tuneable Reflective Structural Coloration with Electroactive Conducting Polymer Nanocavities. Adv. Mater. 2021, 33, 2105004 10.1002/adma.202105004. PMC11469130 34626028

[ref275] Atighilorestani M.; dos Santos D. P.; Jaimes R. F. V. V.; Rahman M. M.; Temperini M. L. A.; Brolo A. G. Electrochemical Control of Light Transmission through Nanohole Electrode Arrays. ACS Photon. 2016, 3, 2375–2382. 10.1021/acsphotonics.6b00607.

[ref276] Xiong K.; Emilsson G.; Maziz A.; Yang X.; Shao L.; Jager E. W. H.; Dahlin A. B. Plasmonic Metasurfaces with Conjugated Polymers for Flexible Electronic Paper in Color. Adv. Mater. 2016, 28, 9956–9960. 10.1002/adma.201603358. 27670834

[ref277] Atighilorestani M.; Jiang H.; Kaminska B. Electrochromic-Polymer-Based Switchable Plasmonic Color Devices Using Surface-Relief Nanostructure Pixels. Adv. Opt. Mater. 2018, 6, 1801179 10.1002/adom.201801179.

[ref278] Zhou Z.; Yu Y.; Sun N.; Möhwald H.; Gu P.; Wang L.; Zhang W.; König T. A. F.; Fery A.; Zhang G. Broad-Range Electrically Tunable Plasmonic Resonances of a Multilayer Coaxial Nanohole Array with an Electroactive Polymer Wrapper. ACS Appl. Mater. Interfaces 2017, 9, 35244–35252. 10.1021/acsami.7b11139. 28925685

[ref279] Sasaki K.; Nagamura T. Ultrafast Wide Range All-Optical Switch Using Complex Refractive-Index Changes in a Composite Film of Silver and Polymer Containing Photochromic Dye. J. Appl. Phys. 1998, 83, 2894–2900. 10.1063/1.367076.

[ref280] Krasavin A. V.; Randhawa S.; Bouillard J.-S.; Renger J.; Quidant R.; Zayats A. V. Optically-Programmable Nonlinear Photonic Component for Dielectric-Loaded Plasmonic Circuitry. Opt. Express 2011, 19, 25222–25229. 10.1364/OE.19.025222. 22273913

[ref281] Gong S.; Ren M.; Wu W.; Cai W.; Xu J. Optically Addressed Spatial Light Modulator Based on Nonlinear Metasurface. Photon. Res. 2021, 9, 610–614. 10.1364/PRJ.416189.

[ref282] Zhang F.; Hu X.; Zhu Y.; Yang H.; Gong Q. Ultralow-Power All-Optical Tunable Dual Fano Resonances in Nonlinear Metamaterials. Appl. Phys. Lett. 2013, 103, 191116 10.1063/1.4829655.

[ref283] Hedayati M. K.; Javaheri M.; Zillohu A. U.; El-Khozondar H. J.; Bawa’aneh M. S.; Lavrinenko A.; Faupel F.; Elbahri M. Photo-Driven Super Absorber as an Active Metamaterial with a Tunable Molecular-Plasmonic Coupling. Adv. Opt. Mater. 2014, 2, 705–710. 10.1002/adom.201400105.

[ref284] Lin L.; Wang M.; Wei X.; Peng X.; Xie C.; Zheng Y. Photoswitchable Rabi Splitting in Hybrid Plasmon–Waveguide Modes. Nano Lett. 2016, 16, 7655–7663. 10.1021/acs.nanolett.6b03702. 27960522

[ref285] Traverso A. J.; Huang J.; Peyronel T.; Yang G.; Tiecke T. G.; Mikkelsen M. H. Low-Loss, Centimeter-Scale Plasmonic Metasurface for Ultrafast Optoelectronics. Optica 2021, 8, 202–207. 10.1364/OPTICA.400731.

[ref286] Xiao S.; Chettiar U. K.; Kildishev A. V.; Drachev V.; Khoo I. C.; Shalaev V. M. Tunable Magnetic Response of Metamaterials. Appl. Phys. Lett. 2009, 95, 033115 10.1063/1.3182857.

[ref287] Sautter J.; Staude I.; Decker M.; Rusak E.; Neshev D. N.; Brener I.; Kivshar Y. S. Active Tuning of All-Dielectric Metasurfaces. ACS Nano 2015, 9, 4308–4315. 10.1021/acsnano.5b00723. 25748581

[ref288] Kruk S.; Minovich A.; Farnell J.; McKerracher I.; Karouta F.; Tian J.; Powell D. A.; Shadrivov I. V.; Tan H. H.; Jagadish C.; . Tunable and Nonlinear Fishnet Metamaterials Based on Liquid Crystal Infiltration. Proc. SPIE, Metamaterials: Fundamentals and Applications V; 2012; Vol. 8455, p 84552O 10.1117/12.931309.

[ref289] Shrekenhamer D.; Chen W.-C.; Padilla W. J. Liquid Crystal Tunable Metamaterial Absorber. Phys. Rev. Lett. 2013, 110, 177403 10.1103/PhysRevLett.110.177403. 23679774

[ref290] Savo S.; Shrekenhamer D.; Padilla W. J. Liquid Crystal Metamaterial Absorber Spatial Light Modulator for Thz Applications. Adv. Opt. Mater. 2014, 2, 275–279. 10.1002/adom.201300384.

[ref291] Franklin D.; Chen Y.; Vazquez-Guardado A.; Modak S.; Boroumand J.; Xu D.; Wu S.-T.; Chanda D. Polarization-Independent Actively Tunable Colour Generation on Imprinted Plasmonic Surfaces. Nat. Commun. 2015, 6, 7337 10.1038/ncomms8337. 26066375 PMC4490413

[ref292] Wang L.; Lin X.-W.; Hu W.; Shao G.-H.; Chen P.; Liang L.-J.; Jin B.-B.; Wu P.-H.; Qian H.; Lu Y.-N.; et al. Broadband Tunable Liquid Crystal Terahertz Waveplates Driven with Porous Graphene Electrodes. Light Sci. Appl. 2015, 4, e253–e253. 10.1038/lsa.2015.26.

[ref293] Olson J.; Manjavacas A.; Basu T.; Huang D.; Schlather A. E.; Zheng B.; Halas N. J.; Nordlander P.; Link S. High Chromaticity Aluminum Plasmonic Pixels for Active Liquid Crystal Displays. ACS Nano 2016, 10, 1108–1117. 10.1021/acsnano.5b06415. 26639191

[ref294] Komar A.; Fang Z.; Bohn J.; Sautter J.; Decker M.; Miroshnichenko A.; Pertsch T.; Brener I.; Kivshar Y. S.; Staude I.; et al. Electrically Tunable All-Dielectric Optical Metasurfaces Based on Liquid Crystals. Appl. Phys. Lett. 2017, 110, 071109 10.1063/1.4976504.

[ref295] Wu J.; Shen Z.; Ge S.; Chen B.; Shen Z.; Wang T.; Zhang C.; Hu W.; Fan K.; Padilla W.; et al. Liquid Crystal Programmable Metasurface for Terahertz Beam Steering. Appl. Phys. Lett. 2020, 116, 131104 10.1063/1.5144858.

[ref296] Kang B.; Woo J. H.; Choi E.; Lee H.-H.; Kim E. S.; Kim J.; Hwang T.-J.; Park Y.-S.; Kim D. H.; Wu J. W. Optical Switching of near Infrared Light Transmission in Metamaterial-Liquid Crystal Cell Structure. Opt. Express 2010, 18, 16492–16498. 10.1364/OE.18.016492. 20721037

[ref297] Liu Y. J.; Si G. Y.; Leong E. S. P.; Xiang N.; Danner A. J.; Teng J. H. Light-Driven Plasmonic Color Filters by Overlaying Photoresponsive Liquid Crystals on Gold Annular Aperture Arrays. Adv. Mater. 2012, 24, Op131–Op135. 10.1002/adma.201104440. 22438069

[ref298] Liu Y. J.; Ding X. Y.; Lin S. C. S.; Shi J. J.; Chiang I. K.; Huang T. J. Surface Acoustic Wave Driven Light Shutters Using Polymer-Dispersed Liquid Crystals. Adv. Mater. 2011, 23, 1656 10.1002/adma.201003708. 21438028

[ref299] Zhang F. L.; Zhang W. H.; Zhao Q.; Sun J. B.; Qiu K. P.; Zhou J.; Lippens D. Electrically Controllable Fishnet Metamaterial Based on Nematic Liquid Crystal. Opt. Express 2011, 19, 1563–1568. 10.1364/OE.19.001563. 21263696

[ref300] Isić G.; Vasić B.; Zografopoulos D. C.; Beccherelli R.; Gajić R. Electrically Tunable Critically Coupled Terahertz Metamaterial Absorber Based on Nematic Liquid Crystals. Phys. Rev. Appl. 2015, 3, 064007 10.1103/PhysRevApplied.3.064007.

[ref301] Evans P. R.; Wurtz G. A.; Hendren W. R.; Atkinson R.; Dickson W.; Zayats A. V.; Pollard R. J. Electrically Switchable Nonreciprocal Transmission of Plasmonic Nanorods with Liquid Crystal. Appl. Phys. Lett. 2007, 91, 043101 10.1063/1.2759463.

[ref302] Zhao Q.; Kang L.; Du B.; Li B.; Zhou J.; Tang H.; Liang X.; Zhang B. Electrically Tunable Negative Permeability Metamaterials Based on Nematic Liquid Crystals. Appl. Phys. Lett. 2007, 90, 011112 10.1063/1.2430485.

[ref303] Decker M.; Kremers C.; Minovich A.; Staude I.; Miroshnichenko A. E.; Chigrin D.; Neshev D. N.; Jagadish C.; Kivshar Y. S. Electro-Optical Switching by Liquid-Crystal Controlled Metasurfaces. Opt. Express 2013, 21, 8879–8885. 10.1364/OE.21.008879. 23571978

[ref304] Buchnev O.; Podoliak N.; Kaczmarek M.; Zheludev N. I.; Fedotov V. A. Electrically Controlled Nanostructured Metasurface Loaded with Liquid Crystal: Toward Multifunctional Photonic Switch. Adv. Opt. Mater. 2015, 3, 674–679. 10.1002/adom.201400494.

[ref305] Wang Q.; Zhang X. G.; Tian H. W.; Jiang W. X.; Bao D.; Jiang H. L.; Luo Z. J.; Wu L. T.; Cui T. J. Millimeter-Wave Digital Coding Metasurfaces Based on Nematic Liquid Crystals. Adv. Theory Simul. 2019, 2, 1900141 10.1002/adts.201900141.

[ref306] Vasić B.; Isić G.; Beccherelli R.; Zografopoulos D. C. Tunable Beam Steering at Terahertz Frequencies Using Reconfigurable Metasurfaces Coupled with Liquid Crystals. IEEE J. Sel. Top. Quant. Electron 2020, 26, 1–9. 10.1109/JSTQE.2019.2956856.

[ref307] Vasić B.; Zografopoulos D. C.; Isić G.; Beccherelli R.; Gajić R. Electrically Tunable Terahertz Polarization Converter Based on Overcoupled Metal-Isolator-Metal Metamaterials Infiltrated with Liquid Crystals. Nanotechnology 2017, 28, 124002 10.1088/1361-6528/aa5bbd. 28220761

[ref308] Wang L.; Ge S.; Hu W.; Nakajima M.; Lu Y. Tunable Reflective Liquid Crystal Terahertz Waveplates. Opt. Mater. Express 2017, 7, 2023–2029. 10.1364/OME.7.002023.

[ref309] Li J.; Yu P.; Zhang S.; Liu N. Electrically-Controlled Digital Metasurface Device for Light Projection Displays. Nat. Commun. 2020, 11, 3574 10.1038/s41467-020-17390-3. 32681122 PMC7367846

[ref310] Sharma M.; Hendler N.; Ellenbogen T. Electrically Switchable Color Tags Based on Active Liquid-Crystal Plasmonic Metasurface Platform. Adv. Opt. Mater. 2020, 8, 1901182 10.1002/adom.201901182.

[ref311] Kowerdziej R.; Wróbel J.; Kula P. Ultrafast Electrical Switching of Nanostructured Metadevice with Dual-Frequency Liquid Crystal. Sci. Rep. 2019, 9, 20367 10.1038/s41598-019-55656-z. 31889047 PMC6937344

[ref312] Zhang F.; Zhao Q.; Kang L.; Gaillot D. P.; Zhao X.; Zhou J.; Lippens D. Magnetic Control of Negative Permeability Metamaterials Based on Liquid Crystals. Appl. Phys. Lett. 2008, 92, 193104 10.1063/1.2926678.

[ref313] Dickson W.; Evans P. R.; Wurtz G. A.; Hendren W.; Atkinson R.; Pollard R. J.; Zayats A. V. Towards Nonlinear Plasmonic Devices Based on Metallic Nanorods. J. Microsc. 2008, 229, 415–420. 10.1111/j.1365-2818.2008.01921.x. 18331488

[ref314] Lan S.; Rodrigues S.; Cui Y.; Kang L.; Cai W. Electrically Tunable Harmonic Generation of Light from Plasmonic Structures in Electrolytes. Nano Lett. 2016, 16, 5074–5079. 10.1021/acs.nanolett.6b01940. 27398925

[ref315] Vasić B.; Gajić R. Optical Modulation Based on Tunable Light Absorption and Amplification in Metasurfaces Coupled with Gain Medium. Opt. Lett. 2017, 42, 2181–2184. 10.1364/OL.42.002181. 28569876

[ref316] Pirruccio G.; Ramezani M.; Rodriguez S. R. K.; Rivas J. G. Coherent Control of the Optical Absorption in a Plasmonic Lattice Coupled to a Luminescent Layer. Phys. Rev. Lett. 2016, 116, 103002 10.1103/PhysRevLett.116.103002. 27015478

[ref317] He J. N.; Wang J. Q.; Ding P.; Fan C. Z.; Liang E. J. Gain-Assisted Plasmon Induced Transparency in T-Shaped Metamaterials for Slow Light. J. Opt. 2015, 17, 055002 10.1088/2040-8978/17/5/055002.

[ref318] Ginzburg P.; Rodríguez-Fortuño F. J.; Martínez A.; Zayats A. V. Analogue of the Quantum Hanle Effect and Polarization Conversion in Non-Hermitian Plasmonic Metamaterials. Nano Lett. 2012, 12, 6309–6314. 10.1021/nl3034174. 23163587

[ref319] Sakhdari M.; Farhat M.; Chen P.-Y. PT-Symmetric Metasurfaces: Wave Manipulation and Sensing Using Singular Points. New J. Phys. 2017, 19, 065002 10.1088/1367-2630/aa6bb9.

[ref320] Sounas D. L.; Fleury R.; Alù A. Unidirectional Cloaking Based on Metasurfaces with Balanced Loss and Gain. Phys. Rev. Appl. 2015, 4, 014005 10.1103/PhysRevApplied.4.014005.

[ref321] Savoia S.; Castaldi G.; Galdi V.; Alù A.; Engheta N. PT-Symmetry-Induced Wave Confinement and Guiding in Ε-near-Zero Metamaterials. Phys. Rev. B 2015, 91, 115114 10.1103/PhysRevB.91.115114.

[ref322] Tapar J.; Kishen S.; Emani N. K. Dynamically Tunable Asymmetric Transmission in PT-Symmetric Phase Gradient Metasurface. ACS Photonics 2021, 8, 3315–3322. 10.1021/acsphotonics.1c01178.

[ref323] Wang J. X.; Shen Y.; Yu X.; Zou L. E.; Ouyang S. J.; Deng X. H. Active Control of Parity-Time Symmetry Phase Transition in Terahertz Metasurface. Phys. Lett. A 2021, 400, 127304 10.1016/j.physleta.2021.127304.

[ref324] Valagiannopoulos C. A.; Monticone F.; Alù A. PT-Symmetric Planar Devices for Field Transformation and Imaging. J. Opt. 2016, 18, 044028 10.1088/2040-8978/18/4/044028.

[ref325] Tsironis G. P.; Lazarides N. PT-Symmetric Nonlinear Metamaterials and Zero-Dimensional Systems. Appl. Phys. A: Mater. Sci. Process. 2014, 115, 449–458. 10.1007/s00339-013-8035-2.

[ref326] El-Ganainy R.; Makris K. G.; Khajavikhan M.; Musslimani Z. H.; Rotter S.; Christodoulides D. N. Non-Hermitian Physics and PT Symmetry. Nat. Phys. 2018, 14, 11–19. 10.1038/nphys4323.

[ref327] Zhao H.; Longhi S.; Feng L. Robust Light State by Quantum Phase Transition in Non-Hermitian Optical Materials. Sci. Rep. 2015, 5, 17022 10.1038/srep17022. 26592765 PMC4655477

[ref328] Wurtz G. A.; Pollard R.; Zayats A. V. Optical Bistability in Nonlinear Surface-Plasmon Polaritonic Crystals. Phys. Rev. Lett. 2006, 97, 057402 10.1103/PhysRevLett.97.057402. 17026140

[ref329] Vasilantonakis N.; Wurtz G. A.; Podolskiy V. A.; Zayats A. V. Refractive Index Sensing with Hyperbolic Metamaterials: Strategies for Biosensing and Nonlinearity Enhancement. Opt. Express 2015, 23, 14329–14343. 10.1364/OE.23.014329. 26072797

[ref330] Wurtz G. A.; Zayats A. V. Nonlinear Surface Plasmon Polaritonic Crystals. Laser Photonics Rev. 2008, 2, 125–135. 10.1002/lpor.200810006.

[ref331] Gordon J. G.; Ernst S. Surface-Plasmons as a Probe of the Electrochemical Interface. Surf. Sci. 1980, 101, 499–506. 10.1016/0039-6028(80)90644-5.

[ref332] Nylander C.; Liedberg B.; Lind T. Gas-Detection by Means of Surface-Plasmon Resonance. Sens. Actuators 1982, 3, 79–88. 10.1016/0250-6874(82)80008-5.

[ref333] Homola J.; Piliarik M. Surface Plasmon Resonance (SPR) Sensors; Springer, 2006; pp 45–67. 10.1364/OE.17.016505 19770865

[ref334] Prabowo B. A.; Purwidyantri A.; Liu K.-C. Surface Plasmon Resonance Optical Sensor: A Review on Light Source Technology. Biosensors 2018, 8, 80 10.3390/bios8030080. PMC6163427 30149679

[ref335] Anker J. N.; Hall W. P.; Lyandres O.; Shah N. C.; Zhao J.; Van Duyne R. P. Biosensing with Plasmonic Nanosensors. Nat. Mater. 2008, 7, 442–453. 10.1038/nmat2162. 18497851

[ref336] Chen H.; Shao L.; Woo K. C.; Ming T.; Lin H.-Q.; Wang J. Shape-Dependent Refractive Index Sensitivities of Gold Nanocrystals with the Same Plasmon Resonance Wavelength. J. Phys. Chem. C 2009, 113, 17691–17697. 10.1021/jp907413n.

[ref337] Wu P. C.; Liao C. Y.; Chen J.-W.; Tsai D. P. Isotropic Absorption and Sensor of Vertical Split-Ring Resonator. Adv. Opt. Mater. 2017, 5, 1600581 10.1002/adom.201600581.

[ref338] Wang W.; Yan F.; Tan S.; Zhou H.; Hou Y. Ultrasensitive Terahertz Metamaterial Sensor Based on Vertical Split Ring Resonators. Photon. Res. 2017, 5, 571–577. 10.1364/PRJ.5.000571.

[ref339] Maeda E.; Mikuriya S.; Endo K.; Yamada I.; Suda A.; Delaunay J.-J. Optical Hydrogen Detection with Periodic Subwavelength Palladium Hole Arrays. Appl. Phys. Lett. 2009, 95, 133504 10.1063/1.3224890.

[ref340] Escobedo C.; Brolo A. G.; Gordon R.; Sinton D. Optofluidic Concentration: Plasmonic Nanostructure as Concentrator and Sensor. Nano Lett. 2012, 12, 1592–1596. 10.1021/nl204504s. 22352888

[ref341] Fang J.; Levchenko I.; Yan W.; Aharonovich I.; Aramesh M.; Prawer S.; Ostrikov K. Plasmonic Metamaterial Sensor with Ultra-High Sensitivity in the Visible Spectral Range. Adv. Opt. Mater. 2015, 3, 750–755. 10.1002/adom.201400577.

[ref342] Kabashin A. V.; Evans P.; Pastkovsky S.; Hendren W.; Wurtz G. A.; Atkinson R.; Pollard R.; Podolskiy V. A.; Zayats A. V. Plasmonic Nanorod Metamaterials for Biosensing. Nat. Mater. 2009, 8, 867–871. 10.1038/nmat2546. 19820701

[ref343] Sreekanth K. V.; Alapan Y.; ElKabbash M.; Ilker E.; Hinczewski M.; Gurkan U. A.; De Luca A.; Strangi G. Extreme Sensitivity Biosensing Platform Based on Hyperbolic Metamaterials. Nat. Mater. 2016, 15, 621–627. 10.1038/nmat4609. 27019384 PMC4959915

[ref344] Sreekanth K. V.; Alapan Y.; ElKabbash M.; Wen A. M.; Ilker E.; Hinczewski M.; Gurkan U. A.; Steinmetz N. F.; Strangi G. Enhancing the Angular Sensitivity of Plasmonic Sensors Using Hyperbolic Metamaterials. Adv. Opt. Mater. 2016, 4, 1767–1772. 10.1002/adom.201600448. 28649484 PMC5482536

[ref345] Jiang L.; Zeng S.; Xu Z.; Ouyang Q.; Zhang D.-H.; Chong P. H. J.; Coquet P.; He S.; Yong K.-T. Multifunctional Hyperbolic Nanogroove Metasurface for Submolecular Detection. Small 2017, 13, 1700600 10.1002/smll.201700600. 28597602

[ref346] Sreekanth K. V.; Mahalakshmi P.; Han S.; Mani Rajan M. S.; Choudhury P. K.; Singh R. Brewster Mode-Enhanced Sensing with Hyperbolic Metamaterial. Adv. Opt. Mater. 2019, 7, 1900680 10.1002/adom.201900680.

[ref347] Palermo G.; Sreekanth K. V.; Maccaferri N.; Lio G. E.; Nicoletta G.; De Angelis F.; Hinczewski M.; Strangi G. Hyperbolic Dispersion Metasurfaces for Molecular Biosensing. Nanophotonics 2020, 10, 295–314. 10.1515/nanoph-2020-0466.

[ref348] Wang P.; Krasavin A. V.; Viscomi F. N.; Adawi A. M.; Bouillard J.-S. G.; Zhang L.; Roth D. J.; Tong L.; Zayats A. V. Metaparticles: Dressing Nano-Objects with a Hyperbolic Coating. Laser Photonics Rev. 2018, 12, 1800179 10.1002/lpor.201800179.

[ref349] Sreekanth K. V.; ElKabbash M.; Alapan Y.; Ilker E. I.; Hinczewski M.; Gurkan U. A.; Strangi G. Hyperbolic Metamaterials-Based Plasmonic Biosensor for Fluid Biopsy with Single Molecule Sensitivity. EPJ. Appl. Metamaterials 2017, 4, 1 10.1051/epjam/2016015.

[ref350] Santos D. F.; Guerreiro A.; Baptista J. M. SPR Optimization Using Metamaterials in a D-Type PCF Refractive Index Sensor. Opt. Fiber Technol. 2017, 33, 83–88. 10.1016/j.yofte.2016.11.010.

[ref351] Hu S.; Chen Y.; Chen Y.; Chen L.; Zheng H.; Azeman N. H.; Liu M. X.; Liu G.-S.; Luo Y.; Chen Z. High-Performance Fiber Plasmonic Sensor by Engineering the Dispersion of Hyperbolic Metamaterials Composed of Ag/TiO_2_. Opt. Express 2020, 28, 25562–25573. 10.1364/OE.397461. 32907073

[ref352] Li C.; Gao J.; Shafi M.; Liu R.; Zha Z.; Feng D.; Liu M.; Du X.; Yue W.; Jiang S. Optical Fiber SPR Biosensor Complying with a 3D Composite Hyperbolic Metamaterial and a Graphene Film. Photon. Res. 2021, 9, 379–388. 10.1364/PRJ.416815.

[ref353] Yang W.; Gao J.; Li Z.; Li C.; Cheng Y.; Huo Y.; Jiang S.; Jiang M. High Performance D-Type Plastic Fiber SPR Sensor Based on a Hyperbolic Metamaterial Composed of Ag/MgF_2_. J. Mater. Chem. C 2021, 9, 13647–13658. 10.1039/D1TC02217B.

[ref354] Hao F.; Sonnefraud Y.; Dorpe P. V.; Maier S. A.; Halas N. J.; Nordlander P. Symmetry Breaking in Plasmonic Nanocavities: Subradiant Lspr Sensing and a Tunable Fano Resonance. Nano Lett. 2008, 8, 3983–3988. 10.1021/nl802509r. 18831572

[ref355] Hao F.; Nordlander P.; Sonnefraud Y.; Dorpe P. V.; Maier S. A. Tunability of Subradiant Dipolar and Fano-Type Plasmon Resonances in Metallic Ring/Disk Cavities: Implications for Nanoscale Optical Sensing. ACS Nano 2009, 3, 643–652. 10.1021/nn900012r. 19309172

[ref356] Sonnefraud Y.; Verellen N.; Sobhani H.; Vandenbosch G. A. E.; Moshchalkov V. V.; Van Dorpe P.; Nordlander P.; Maier S. A. Experimental Realization of Subradiant, Superradiant, and Fano Resonances in Ring/Disk Plasmonic Nanocavities. ACS Nano 2010, 4, 1664–1670. 10.1021/nn901580r. 20155967

[ref357] Butet J.; Martin O. J. F. Refractive Index Sensing with Fano Resonant Plasmonic Nanostructures: A Symmetry Based Nonlinear Approach. Nanoscale 2014, 6, 15262–15270. 10.1039/C4NR05623J. 25381752

[ref358] Fedotov V. A.; Rose M.; Prosvirnin S. L.; Papasimakis N.; Zheludev N. I. Sharp Trapped-Mode Resonances in Planar Metamaterials with a Broken Structural Symmetry. Phys. Rev. Lett. 2007, 99, 147401 10.1103/PhysRevLett.99.147401. 17930720

[ref359] Zhao J.; Zhang C.; Braun P. V.; Giessen H. Large-Area Low-Cost Plasmonic Nanostructures in the Nir for Fano Resonant Sensing. Adv. Mater. 2012, 24, OP247–OP252. 10.1002/adma.201202109. 22807217

[ref360] Mirin N. A.; Bao K.; Nordlander P. Fano Resonances in Plasmonic Nanoparticle Aggregates. J. Phys. Chem. A 2009, 113, 4028–4034. 10.1021/jp810411q. 19371111

[ref361] Hentschel M.; Saliba M.; Vogelgesang R.; Giessen H.; Alivisatos A. P.; Liu N. Transition from Isolated to Collective Modes in Plasmonic Oligomers. Nano Lett. 2010, 10, 2721–2726. 10.1021/nl101938p. 20586409

[ref362] Zhang Y.; Zhen Y.-R.; Neumann O.; Day J. K.; Nordlander P.; Halas N. J. Coherent Anti-Stokes Raman Scattering with Single-Molecule Sensitivity Using a Plasmonic Fano Resonance. Nat. Commun. 2014, 5, 4424 10.1038/ncomms5424. 25020075

[ref363] Liu N.; Weiss T.; Mesch M.; Langguth L.; Eigenthaler U.; Hirscher M.; Sönnichsen C.; Giessen H. Planar Metamaterial Analogue of Electromagnetically Induced Transparency for Plasmonic Sensing. Nano Lett. 2010, 10, 1103–1107. 10.1021/nl902621d. 20017551

[ref364] Papasimakis N.; Luo Z.; Shen Z. X.; De Angelis F.; Di Fabrizio E.; Nikolaenko A. E.; Zheludev N. I. Graphene in a Photonic Metamaterial. Opt. Express 2010, 18, 8353–8359. 10.1364/OE.18.008353. 20588680

[ref365] Cetin A. E.; Altug H. Fano Resonant Ring/Disk Plasmonic Nanocavities on Conducting Substrates for Advanced Biosensing. ACS Nano 2012, 6, 9989–9995. 10.1021/nn303643w. 23092386

[ref366] Wu C. H.; Khanikaev A. B.; Adato R.; Arju N.; Yanik A. A.; Altug H.; Shvets G. Fano-Resonant Asymmetric Metamaterials for Ultrasensitive Spectroscopy and Identification of Molecular Monolayers. Nat. Mater. 2012, 11, 69–75. 10.1038/nmat3161. 22081082

[ref367] Chen J.; Gan F.; Wang Y.; Li G. Plasmonic Sensing and Modulation Based on Fano Resonances. Adv. Opt. Mater. 2018, 6, 1701152 10.1002/adom.201701152.

[ref368] Yanik A. A.; Cetin A. E.; Huang M.; Artar A.; Mousavi S. H.; Khanikaev A.; Connor J. H.; Shvets G.; Altug H. Seeing Protein Monolayers with Naked Eye through Plasmonic Fano Resonances. Proc. Natl. Acad. Sci. U.S.A. 2011, 108, 11784–11789. 10.1073/pnas.1101910108. 21715661 PMC3141965

[ref369] Shen Y.; Zhou J.; Liu T.; Tao Y.; Jiang R.; Liu M.; Xiao G.; Zhu J.; Zhou Z.-K.; Wang X.; et al. Plasmonic Gold Mushroom Arrays with Refractive Index Sensing Figures of Merit Approaching the Theoretical Limit. Nat. Commun. 2013, 4, 2381 10.1038/ncomms3381. 23979039

[ref370] Tetz K. A.; Pang L.; Fainman Y. High-Resolution Surface Plasmon Resonance Sensor Based on Linewidth-Optimized Nanohole Array Transmittance. Opt. Lett. 2006, 31, 1528–1530. 10.1364/OL.31.001528. 16642161

[ref371] Homola J. Surface Plasmon Resonance Based Sensors; Springer-Verlag Berlin: Heidelberg, 2006.

[ref372] Yang A.; Huntington M. D.; Cardinal M. F.; Masango S. S.; Van Duyne R. P.; Odom T. W. Hetero-Oligomer Nanoparticle Arrays for Plasmon-Enhanced Hydrogen Sensing. ACS Nano 2014, 8, 7639–7647. 10.1021/nn502502r. 24956125

[ref373] Tittl A.; Giessen H.; Liu N. Plasmonic Gas and Chemical Sensing. Nanophotonics 2014, 3, 157–180. 10.1515/nanoph-2014-0002.

[ref374] Powell A. W.; Coles D. M.; Taylor R. A.; Watt A. A. R.; Assender H. E.; Smith J. M. Plasmonic Gas Sensing Using Nanocube Patch Antennas. Adv. Opt. Mater. 2016, 4, 634–642. 10.1002/adom.201500602.

[ref375] Tittl A.; Mai P.; Taubert R.; Dregely D.; Liu N.; Giessen H. Palladium-Based Plasmonic Perfect Absorber in the Visible Wavelength Range and Its Application to Hydrogen Sensing. Nano Lett. 2011, 11, 4366–4369. 10.1021/nl202489g. 21877697

[ref376] Luong H. M.; Pham M. T.; Guin T.; Madhogaria R. P.; Phan M. H.; Larsen G. K.; Nguyen T. D. Sub-Second and ppm-Level Optical Sensing of Hydrogen Using Templated Control of Nano-Hydride Geometry and Composition. Nat. Commun. 2021, 12, 2414 10.1038/s41467-021-22697-w. 33893313 PMC8065102

[ref377] Nau D.; Seidel A.; Orzekowsky R. B.; Lee S. H.; Deb S.; Giessen H. Hydrogen Sensor Based on Metallic Photonic Crystal Slabs. Opt. Lett. 2010, 35, 3150–3152. 10.1364/OL.35.003150. 20847808

[ref378] Yu J. C.; Sun J.; Chandrasekaran N.; Dunn C. J.; Chesman A. S. R.; Jasieniak J. J. Semi-Transparent Perovskite Solar Cells with a Cross-Linked Hole Transport Layer. Nano Energy 2020, 71, 104635 10.1016/j.nanoen.2020.104635.

[ref379] Hierro-Rodriguez A.; Leite I. T.; Rocha-Rodrigues P.; Fernandes P.; Araujo J. P.; Jorge P. A. S.; Santos J. L.; Teixeira J. M.; Guerreiro A. Hydrogen Sensing Via Anomalous Optical Absorption of Palladium-Based Metamaterials. Nanotechnology 2016, 27, 185501 10.1088/0957-4484/27/18/185501. 27003717

[ref380] Rocha-Rodrigues P.; Hierro-Rodriguez A.; Guerreiro A.; Jorge P.; Santos J. L.; Araújo J. P.; Teixeira J. M. Hydrogen Optical Metamaterial Sensor Based on Pd Dendritic Nanostructures. ChemistrySelect 2016, 1, 3854–3860. 10.1002/slct.201600833.

[ref381] Matuschek M.; Singh D. P.; Jeong H.-H.; Nesterov M.; Weiss T.; Fischer P.; Neubrech F.; Liu N. Chiral Plasmonic Hydrogen Sensors. Small 2018, 14, 1702990 10.1002/smll.201702990. 29266737

[ref382] Bagheri S.; Strohfeldt N.; Sterl F.; Berrier A.; Tittl A.; Giessen H. Large-Area Low-Cost Plasmonic Perfect Absorber Chemical Sensor Fabricated by Laser Interference Lithography. ACS Sens. 2016, 1, 1148–1154. 10.1021/acssensors.6b00444.

[ref383] Beni T.; Yamasaku N.; Kurotsu T.; To N.; Okazaki S.; Arakawa T.; Balčytis A.; Seniutinas G.; Juodkazis S.; Nishijima Y. Metamaterial for Hydrogen Sensing. ACS Sens. 2019, 4, 2389–2394. 10.1021/acssensors.9b00980. 31412698

[ref384] Sterl F.; Strohfeldt N.; Both S.; Herkert E.; Weiss T.; Giessen H. Design Principles for Sensitivity Optimization in Plasmonic Hydrogen Sensors. ACS Sens. 2020, 5, 917–927. 10.1021/acssensors.9b02436. 31997641

[ref385] Nugroho F. A. A.; Eklund R.; Nilsson S.; Langhammer C. A Fiber-Optic Nanoplasmonic Hydrogen Sensor Via Pattern-Transfer of Nanofabricated Pdau Alloy Nanostructures. Nanoscale 2018, 10, 20533–20539. 10.1039/C8NR03751E. 30397701

[ref386] Nugroho F. A. A.; Darmadi I.; Cusinato L.; Susarrey-Arce A.; Schreuders H.; Bannenberg L. J.; da Silva Fanta A. B.; Kadkhodazadeh S.; Wagner J. B.; Antosiewicz T. J.; et al. Metal–Polymer Hybrid Nanomaterials for Plasmonic Ultrafast Hydrogen Detection. Nat. Mater. 2019, 18, 489–495. 10.1038/s41563-019-0325-4. 30936481

[ref387] Liu N.; Tang M. L.; Hentschel M.; Giessen H.; Alivisatos A. P. Nanoantenna-Enhanced Gas Sensing in a Single Tailored Nanofocus. Nat. Mater. 2011, 10, 631–636. 10.1038/nmat3029. 21572410

[ref388] Tittl A.; Kremers C.; Dorfmüller J.; Chigrin D. N.; Giessen H. Spectral Shifts in Optical Nanoantenna-Enhanced Hydrogen Sensors. Opt. Mater. Express 2012, 2, 111–118. 10.1364/OME.2.000111.

[ref389] Gschneidtner T. A.; Fernandez Y. A. D.; Syrenova S.; Westerlund F.; Langhammer C.; Moth-Poulsen K. A Versatile Self-Assembly Strategy for the Synthesis of Shape-Selected Colloidal Noble Metal Nanoparticle Heterodimers. Langmuir 2014, 30, 3041–3050. 10.1021/la5002754. 24580549 PMC3982509

[ref390] Larsson E. M.; Langhammer C.; Zorić I.; Kasemo B. Nanoplasmonic Probes of Catalytic Reactions. Science 2009, 326, 1091–1094. 10.1126/science.1176593. 19933104

[ref391] Langhammer C.; Larsson E. M.; Kasemo B.; Zorić I. Indirect Nanoplasmonic Sensing: Ultrasensitive Experimental Platform for Nanomaterials Science and Optical Nanocalorimetry. Nano Lett. 2010, 10, 3529–3538. 10.1021/nl101727b. 20718400

[ref392] Shegai T.; Johansson P.; Langhammer C.; Käll M. Directional Scattering and Hydrogen Sensing by Bimetallic Pd–Au Nanoantennas. Nano Lett. 2012, 12, 2464–2469. 10.1021/nl300558h. 22449167

[ref393] Syrenova S.; Wadell C.; Langhammer C. Shrinking-Hole Colloidal Lithography: Self-Aligned Nanofabrication of Complex Plasmonic Nanoantennas. Nano Lett. 2014, 14, 2655–2663. 10.1021/nl500514y. 24697350

[ref394] Strohfeldt N.; Zhao J.; Tittl A.; Giessen H. Sensitivity Engineering in Direct Contact Palladium-Gold Nano-Sandwich Hydrogen Sensors. Opt. Mater. Express 2015, 5, 2525–2535. 10.1364/OME.5.002525.

[ref395] Wadell C.; Langhammer C. Drift-Corrected Nanoplasmonic Hydrogen Sensing by Polarization. Nanoscale 2015, 7, 10963–10969. 10.1039/C5NR01818H. 26059393

[ref396] Tittl A.; Yin X.; Giessen H.; Tian X.-D.; Tian Z.-Q.; Kremers C.; Chigrin D. N.; Liu N. Plasmonic Smart Dust for Probing Local Chemical Reactions. Nano Lett. 2013, 13, 1816–1821. 10.1021/nl4005089. 23458121

[ref397] Yashna S.; Mangesh J.; Rajib G.; Anuj D. Hydrogen Sensors Based on Plasmonic Nanostructures Present on Palladium Films. Proc. SPIE Optical Sensors; 2019; p 110282Z.

[ref398] Tang M. L.; Liu N.; Dionne J. A.; Alivisatos A. P. Observations of Shape-Dependent Hydrogen Uptake Trajectories from Single Nanocrystals. J. Am. Chem. Soc. 2011, 133, 13220–13223. 10.1021/ja203215b. 21793566

[ref399] Chiu C.-Y.; Huang M. H. Polyhedral Au–Pd Core–Shell Nanocrystals as Highly Spectrally Responsive and Reusable Hydrogen Sensors in Aqueous Solution. Angew. Chem., Int. Ed. 2013, 52, 12709–12713. 10.1002/anie.201306363. 24123474

[ref400] Chiu C.-Y.; Yang M.-Y.; Lin F.-C.; Huang J.-S.; Huang M. H. Facile Synthesis of Au–Pd Core–Shell Nanocrystals with Systematic Shape Evolution and Tunable Size for Plasmonic Property Examination. Nanoscale 2014, 6, 7656–7665. 10.1039/c4nr01765j. 24898776

[ref401] Nie S.; Emory S. R. Probing Single Molecules and Single Nanoparticles by Surface-Enhanced Raman Scattering. Science 1997, 275, 1102–1106. 10.1126/science.275.5303.1102. 9027306

[ref402] Xu H.; Bjerneld E. J.; Käll M.; Börjesson L. Spectroscopy of Single Hemoglobin Molecules by Surface Enhanced Raman Scattering. Phys. Rev. Lett. 1999, 83, 4357–4360. 10.1103/PhysRevLett.83.4357.

[ref403] Li J. F.; Huang Y. F.; Ding Y.; Yang Z. L.; Li S. B.; Zhou X. S.; Fan F. R.; Zhang W.; Zhou Z. Y.; Wu D. Y.; et al. Shell-Isolated Nanoparticle-Enhanced Raman Spectroscopy. Nature 2010, 464, 392–395. 10.1038/nature08907. 20237566

[ref404] Lim D.-K.; Jeon K.-S.; Kim H. M.; Nam J.-M.; Suh Y. D. Nanogap-Engineerable Raman-Active Nanodumbbells for Single-Molecule Detection. Nat. Mater. 2010, 9, 60–67. 10.1038/nmat2596. 20010829

[ref405] Zhang R.; Zhang Y.; Dong Z. C.; Jiang S.; Zhang C.; Chen L. G.; Zhang L.; Liao Y.; Aizpurua J.; Luo Y.; et al. Chemical Mapping of a Single Molecule by Plasmon-Enhanced Raman Scattering. Nature 2013, 498, 82–86. 10.1038/nature12151. 23739426

[ref406] Hatab N. A.; Hsueh C.-H.; Gaddis A. L.; Retterer S. T.; Li J.-H.; Eres G.; Zhang Z.; Gu B. Free-Standing Optical Gold Bowtie Nanoantenna with Variable Gap Size for Enhanced Raman Spectroscopy. Nano Lett. 2010, 10, 4952–4955. 10.1021/nl102963g. 21090585

[ref407] Yang K.; Wang J.; Yao X.; Lyu D.; Zhu J.; Yang Z.; Liu B.; Ren B. Large-Area Plasmonic Metamaterial with Thickness-Dependent Absorption. Adv. Opt. Mater. 2021, 9, 2001375 10.1002/adom.202001375.

[ref408] Wang Y.; Zhao C.; Wang J.; Luo X.; Xie L.; Zhan S.; Kim J.; Wang X.; Liu X.; Ying Y. Wearable Plasmonic-Metasurface Sensor for Noninvasive and Universal Molecular Fingerprint Detection on Biointerfaces. Sci. Adv. 2021, 7, eabe4553 10.1126/sciadv.abe4553. 33523953 PMC10964967

[ref409] Cao C.; Zhang J.; Wen X.; Dodson S. L.; Dao N. T.; Wong L. M.; Wang S.; Li S.; Phan A. T.; Xiong Q. Metamaterials-Based Label-Free Nanosensor for Conformation and Affinity Biosensing. ACS Nano 2013, 7, 7583–7591. 10.1021/nn401645t. 23952283

[ref410] Wen X.; Li G.; Zhang J.; Zhang Q.; Peng B.; Wong L. M.; Wang S.; Xiong Q. Transparent Free-Standing Metamaterials and Their Applications in Surface-Enhanced Raman Scattering. Nanoscale 2014, 6, 132–139. 10.1039/C3NR04012G. 24192898

[ref411] Cecchini M. P.; Turek V. A.; Paget J.; Kornyshev A. A.; Edel J. B. Self-Assembled Nanoparticle Arrays for Multiphase Trace Analyte Detection. Nat. Mater. 2013, 12, 165–171. 10.1038/nmat3488. 23160268

[ref412] Kim K.; Han H. S.; Choi I.; Lee C.; Hong S.; Suh S.-H.; Lee L. P.; Kang T. Interfacial Liquid-State Surface-Enhanced Raman Spectroscopy. Nat. Commun. 2013, 4, 2182 10.1038/ncomms3182. 23864000

[ref413] Ma Y.; Sikdar D.; Fedosyuk A.; Velleman L.; Klemme D. J.; Oh S.-H.; Kucernak A. R. J.; Kornyshev A. A.; Edel J. B. Electrotunable Nanoplasmonics for Amplified Surface Enhanced Raman Spectroscopy. ACS Nano 2020, 14, 328–336. 10.1021/acsnano.9b05257. 31808672

[ref414] Lee S. J.; Guan Z.; Xu H.; Moskovits M. Surface-Enhanced Raman Spectroscopy and Nanogeometry: The Plasmonic Origin of SERS. J. Phys. Chem. C 2007, 111, 17985–17988. 10.1021/jp077422g.

[ref415] Doherty M. D.; Murphy A.; McPhillips J.; Pollard R. J.; Dawson P. Wavelength Dependence of Raman Enhancement from Gold Nanorod Arrays: Quantitative Experiment and Modeling of a Hot Spot Dominated System. J. Phys. Chem. C 2010, 114, 19913–19919. 10.1021/jp107063x.

[ref416] Yue W.; Wang Z.; Whittaker J.; Lopez-royo F.; Yang Y.; Zayats A. V. Amplification of Surface-Enhanced Raman Scattering Due to Substrate-Mediated Localized Surface Plasmons in Gold Nanodimers. J. Mater. Chem. C 2017, 5, 4075–4084. 10.1039/C7TC00667E.

[ref417] Mao L.; Li Z.; Wu B.; Xu H. Effects of Quantum Tunneling in Metal Nanogap on Surface-Enhanced Raman Scattering. Appl. Phys. Lett. 2009, 94, 243102 10.1063/1.3155157.

[ref418] Zhang X.; Zheng Y.; Liu X.; Lu W.; Dai J.; Lei D. Y.; MacFarlane D. R. Hierarchical Porous Plasmonic Metamaterials for Reproducible Ultrasensitive Surface-Enhanced Raman Spectroscopy. Adv. Mater. 2015, 27, 1090–1096. 10.1002/adma.201404107. 25534763

[ref419] Natan M. J. Concluding Remarks Surface Enhanced Raman Scattering. Faraday Discuss. 2006, 132, 321–328. 10.1039/b601494c. 16833126

[ref420] Rodrigo D.; Limaj O.; Janner D.; Etezadi D.; García de Abajo F. J.; Pruneri V.; Altug H. Mid-Infrared Plasmonic Biosensing with Graphene. Science 2015, 349, 165–168. 10.1126/science.aab2051. 26160941

[ref421] Hartstein A.; Kirtley J. R.; Tsang J. C. Enhancement of the Infrared Absorption from Molecular Monolayers with Thin Metal Overlayers. Phys. Rev. Lett. 1980, 45, 201–204. 10.1103/PhysRevLett.45.201.

[ref422] Osawa M.; Ikeda M. Surface-Enhanced Infrared Absorption of P-Nitrobenzoic Acid Deposited on Silver Island Films: Contributions of Electromagnetic and Chemical Mechanisms. J. Phys. Chem. 1991, 95, 9914–9919. 10.1021/j100177a056.

[ref423] Osawa M.; Ataka K.-I.; Yoshii K.; Nishikawa Y. Surface-Enhanced Infrared Spectroscopy: The Origin of the Absorption Enhancement and Band Selection Rule in the Infrared Spectra of Molecules Adsorbed on Fine Metal Particles. Appl. Spectrosc. 1993, 47, 1497–1502. 10.1366/0003702934067478.

[ref424] Jensen T. R.; Van Duyne R. P.; Johnson S. A.; Maroni V. A. Surface-Enhanced Infrared Spectroscopy: A Comparison of Metal Island Films with Discrete and Nondiscrete Surface Plasmons. Appl. Spectrosc. 2000, 54, 371–377. 10.1366/0003702001949654.

[ref425] Enders D.; Pucci A. Surface Enhanced Infrared Absorption of Octadecanethiol on Wet-Chemically Prepared Au Nanoparticle Films. Appl. Phys. Lett. 2006, 88, 184104 10.1063/1.2201880.

[ref426] Tsiatmas A.; Fedotov V. A.; García de Abajo F. J.; Zheludev N. I. Low-Loss Terahertz Superconducting Plasmonics. New J. Phys. 2012, 14, 115006 10.1088/1367-2630/14/11/115006.

[ref427] Coe J. V.; Rodriguez K. R.; Teeters-Kennedy S.; Cilwa K.; Heer J.; Tian H.; Williams S. M. Metal Films with Arrays of Tiny Holes: Spectroscopy with Infrared Plasmonic Scaffolding. J. Phys. Chem. C 2007, 111, 17459–17472. 10.1021/jp072909a.

[ref428] Cubukcu E.; Zhang S.; Park Y.-S.; Bartal G.; Zhang X. Split Ring Resonator Sensors for Infrared Detection of Single Molecular Monolayers. Appl. Phys. Lett. 2009, 95, 043113 10.1063/1.3194154.

[ref429] Pryce I. M.; Kelaita Y. A.; Aydin K.; Atwater H. A. Compliant Metamaterials for Resonantly Enhanced Infrared Absorption Spectroscopy and Refractive Index Sensing. ACS Nano 2011, 5, 8167–8174. 10.1021/nn202815k. 21928788

[ref430] Chen K.; Adato R.; Altug H. Dual-Band Perfect Absorber for Multispectral Plasmon-Enhanced Infrared Spectroscopy. ACS Nano 2012, 6, 7998–8006. 10.1021/nn3026468. 22920565

[ref431] Adato R.; Altug H. In-Situ Ultra-Sensitive Infrared Absorption Spectroscopy of Biomolecule Interactions in Real Time with Plasmonic Nanoantennas. Nat. Commun. 2013, 4, 2154 10.1038/ncomms3154. 23877168 PMC3759039

[ref432] Chen K.; Dao T. D.; Ishii S.; Aono M.; Nagao T. Infrared Aluminum Metamaterial Perfect Absorbers for Plasmon-Enhanced Infrared Spectroscopy. Adv. Funct. Mater. 2015, 25, 6637–6643. 10.1002/adfm.201501151.

[ref433] Limaj O.; Etezadi D.; Wittenberg N. J.; Rodrigo D.; Yoo D.; Oh S.-H.; Altug H. Infrared Plasmonic Biosensor for Real-Time and Label-Free Monitoring of Lipid Membranes. Nano Lett. 2016, 16, 1502–1508. 10.1021/acs.nanolett.5b05316. 26761392

[ref434] Chen K.; Duy Dao T.; Nagao T. Tunable Nanoantennas for Surface Enhanced Infrared Absorption Spectroscopy by Colloidal Lithography and Post-Fabrication Etching. Sci. Rep. 2017, 7, 44069 10.1038/srep44069. 28272442 PMC5341049

[ref435] Hwang I.; Yu J.; Lee J.; Choi J.-H.; Choi D.-G.; Jeon S.; Lee J.; Jung J.-Y. Plasmon-Enhanced Infrared Spectroscopy Based on Metamaterial Absorbers with Dielectric Nanopedestals. ACS Photonics 2018, 5, 3492–3498. 10.1021/acsphotonics.8b00702.

[ref436] Jung Y.; Hwang I.; Yu J.; Lee J.; Choi J.-H.; Jeong J.-H.; Jung J.-Y.; Lee J. Fano Metamaterials on Nanopedestals for Plasmon-Enhanced Infrared Spectroscopy. Sci. Rep. 2019, 9, 7834 10.1038/s41598-019-44396-9. 31127173 PMC6534610

[ref437] Wang H.; Kundu J.; Halas N. J. Plasmonic Nanoshell Arrays Combine Surface-Enhanced Vibrational Spectroscopies on a Single Substrate. Angew. Chem., Int. Ed. 2007, 46, 9040–9044. 10.1002/anie.200702072. 17957664

[ref438] Le F.; Brandl D. W.; Urzhumov Y. A.; Wang H.; Kundu J.; Halas N. J.; Aizpurua J.; Nordlander P. Metallic Nanoparticle Arrays: A Common Substrate for Both Surface-Enhanced Raman Scattering and Surface-Enhanced Infrared Absorption. ACS Nano 2008, 2, 707–718. 10.1021/nn800047e. 19206602

[ref439] Ju L.; Geng B. S.; Horng J.; Girit C.; Martin M.; Hao Z.; Bechtel H. A.; Liang X. G.; Zettl A.; Shen Y. R.; et al. Graphene Plasmonics for Tunable Terahertz Metamaterials. Nat. Nanotechnol. 2011, 6, 630–634. 10.1038/nnano.2011.146. 21892164

[ref440] Chen J.; Badioli M.; Alonso-González P.; Thongrattanasiri S.; Huth F.; Osmond J.; Spasenović M.; Centeno A.; Pesquera A.; Godignon P.; et al. Optical Nano-Imaging of Gate-Tunable Graphene Plasmons. Nature 2012, 487, 77–81. 10.1038/nature11254. 22722861

[ref441] Grigorenko A. N.; Polini M.; Novoselov K. S. Graphene Plasmonics. Nat. Photonics 2012, 6, 749–758. 10.1038/nphoton.2012.262.

[ref442] Li Y.; Yan H.; Farmer D. B.; Meng X.; Zhu W.; Osgood R. M.; Heinz T. F.; Avouris P. Graphene Plasmon Enhanced Vibrational Sensing of Surface-Adsorbed Layers. Nano Lett. 2014, 14, 1573–1577. 10.1021/nl404824w. 24528250

[ref443] Farmer D. B.; Avouris P.; Li Y.; Heinz T. F.; Han S.-J. Ultrasensitive Plasmonic Detection of Molecules with Graphene. ACS Photonics 2016, 3, 553–557. 10.1021/acsphotonics.6b00143.

[ref444] Hu H.; Yang X.; Zhai F.; Hu D.; Liu R.; Liu K.; Sun Z.; Dai Q. Far-Field Nanoscale Infrared Spectroscopy of Vibrational Fingerprints of Molecules with Graphene Plasmons. Nat. Commun. 2016, 7, 12334 10.1038/ncomms12334. 27460765 PMC4974468

[ref445] Luxmoore I. J.; Liu P. Q.; Li P.; Faist J.; Nash G. R. Graphene–Metamaterial Photodetectors for Integrated Infrared Sensing. ACS Photonics 2016, 3, 936–941. 10.1021/acsphotonics.6b00226.

[ref446] Lee I.-H.; Yoo D.; Avouris P.; Low T.; Oh S.-H. Graphene Acoustic Plasmon Resonator for Ultrasensitive Infrared Spectroscopy. Nat. Nanotechnol. 2019, 14, 313–319. 10.1038/s41565-019-0363-8. 30742134

[ref447] Chou L.-W.; Shin N.; Sivaram S. V.; Filler M. A. Tunable Mid-Infrared Localized Surface Plasmon Resonances in Silicon Nanowires. J. Am. Chem. Soc. 2012, 134, 16155–16158. 10.1021/ja3075902. 22985223

[ref448] Baldassarre L.; Sakat E.; Frigerio J.; Samarelli A.; Gallacher K.; Calandrini E.; Isella G.; Paul D. J.; Ortolani M.; Biagioni P. Midinfrared Plasmon-Enhanced Spectroscopy with Germanium Antennas on Silicon Substrates. Nano Lett. 2015, 15, 7225–7231. 10.1021/acs.nanolett.5b03247. 26457387

[ref449] Abb M.; Wang Y.; Papasimakis N.; de Groot C. H.; Muskens O. L. Surface-Enhanced Infrared Spectroscopy Using Metal Oxide Plasmonic Antenna Arrays. Nano Lett. 2014, 14, 346–352. 10.1021/nl404115g. 24341902

[ref450] Law S.; Yu L.; Rosenberg A.; Wasserman D. All-Semiconductor Plasmonic Nanoantennas for Infrared Sensing. Nano Lett. 2013, 13, 4569–4574. 10.1021/nl402766t. 23987983

[ref451] Dobson C. M. Protein Folding and Misfolding. Nature 2003, 426, 884–890. 10.1038/nature02261. 14685248

[ref452] Smith S. W. Chiral Toxicology: It’s the Same Thing···Only Different. Toxicol. Sci. 2009, 110, 4–30. 10.1093/toxsci/kfp097. 19414517

[ref453] Govorov A. O. Plasmon-Induced Circular Dichroism of a Chiral Molecule in the Vicinity of Metal Nanocrystals. Application to Various Geometries. J. Phys. Chem. C 2011, 115, 7914–7923. 10.1021/jp1121432.

[ref454] Hendry E.; Carpy T.; Johnston J.; Popland M.; Mikhaylovskiy R. V.; Lapthorn A. J.; Kelly S. M.; Barron L. D.; Gadegaard N.; Kadodwala M. Ultrasensitive Detection and Characterization of Biomolecules Using Superchiral Fields. Nat. Nanotechnol. 2010, 5, 783–787. 10.1038/nnano.2010.209. 21037572

[ref455] Zhao Y.; Askarpour A. N.; Sun L.; Shi J.; Li X.; Alù A. Chirality Detection of Enantiomers Using Twisted Optical Metamaterials. Nat. Commun. 2017, 8, 14180 10.1038/ncomms14180. 28120825 PMC5288493

[ref456] Karimullah A. S.; Jack C.; Tullius R.; Rotello V. M.; Cooke G.; Gadegaard N.; Barron L. D.; Kadodwala M. Disposable Plasmonics: Plastic Templated Plasmonic Metamaterials with Tunable Chirality. Adv. Mater. 2015, 27, 5610–5616. 10.1002/adma.201501816. 26306427

[ref457] Tullius R.; Karimullah A. S.; Rodier M.; Fitzpatrick B.; Gadegaard N.; Barron L. D.; Rotello V. M.; Cooke G.; Lapthorn A.; Kadodwala M. Superchiral” Spectroscopy: Detection of Protein Higher Order Hierarchical Structure with Chiral Plasmonic Nanostructures. J. Am. Chem. Soc. 2015, 137, 8380–8383. 10.1021/jacs.5b04806. 26102606

[ref458] Hentschel M.; Schäferling M.; Duan X.; Giessen H.; Liu N. Chiral Plasmonics. Sci. Adv. 2017, 3, e1602735 10.1126/sciadv.1602735. 28560336 PMC5435411

[ref459] Tullius R.; Platt G. W.; Khosravi Khorashad L.; Gadegaard N.; Lapthorn A. J.; Rotello V. M.; Cooke G.; Barron L. D.; Govorov A. O.; Karimullah A. S.; et al. Superchiral Plasmonic Phase Sensitivity for Fingerprinting of Protein Interface Structure. ACS Nano 2017, 11, 12049–12056. 10.1021/acsnano.7b04698. 29220155 PMC6034627

[ref460] Kelly C.; Tullius R.; Lapthorn A. J.; Gadegaard N.; Cooke G.; Barron L. D.; Karimullah A. S.; Rotello V. M.; Kadodwala M. Chiral Plasmonic Fields Probe Structural Order of Biointerfaces. J. Am. Chem. Soc. 2018, 140, 8509–8517. 10.1021/jacs.8b03634. 29909628 PMC6070957

[ref461] Yoo S.; Park Q. H. Metamaterials and Chiral Sensing: A Review of Fundamentals and Applications. Nanophotonics 2019, 8, 249–261. 10.1515/nanoph-2018-0167.

[ref462] Lee Y. Y.; Kim R. M.; Im S. W.; Balamurugan M.; Nam K. T. Plasmonic Metamaterials for Chiral Sensing Applications. Nanoscale 2020, 12, 58–66. 10.1039/C9NR08433A. 31815994

[ref463] Clavero C. Plasmon-Induced Hot-Electron Generation at Nanoparticle/Metal-Oxide Interfaces for Photovoltaic and Photocatalytic Devices. Nat. Photonics 2014, 8, 95–103. 10.1038/nphoton.2013.238.

[ref464] Li W.; Valentine J. G. Harvesting the Loss: Surface Plasmon-Based Hot Electron Photodetection. Nanophotonics 2017, 6, 177–191. 10.1515/nanoph-2015-0154.

[ref465] Mubeen S.; Lee J.; Singh N.; Krämer S.; Stucky G. D.; Moskovits M. An Autonomous Photosynthetic Device in Which All Charge Carriers Derive from Surface Plasmons. Nat. Nanotechnol. 2013, 8, 247–251. 10.1038/nnano.2013.18. 23435280

[ref466] Shi X.; Ueno K.; Oshikiri T.; Sun Q.; Sasaki K.; Misawa H. Enhanced Water Splitting under Modal Strong Coupling Conditions. Nat. Nanotechnol. 2018, 13, 953–958. 10.1038/s41565-018-0208-x. 30061658

[ref467] Li W.; Valentine J. Metamaterial Perfect Absorber Based Hot Electron Photodetection. Nano Lett. 2014, 14, 3510–3514. 10.1021/nl501090w. 24837991

[ref468] Ng C.; Cadusch J. J.; Dligatch S.; Roberts A.; Davis T. J.; Mulvaney P.; Gómez D. E. Hot Carrier Extraction with Plasmonic Broadband Absorbers. ACS Nano 2016, 10, 4704–4711. 10.1021/acsnano.6b01108. 26982625

[ref469] Wang W.; Besteiro L. V.; Liu T.; Wu C.; Sun J.; Yu P.; Chang L.; Wang Z.; Govorov A. O. Generation of Hot Electrons with Chiral Metamaterial Perfect Absorbers: Giant Optical Chirality for Polarization-Sensitive Photochemistry. ACS Photonics 2019, 6, 3241–3252. 10.1021/acsphotonics.9b01180.

[ref470] Lee J.; Mubeen S.; Ji X.; Stucky G. D.; Moskovits M. Plasmonic Photoanodes for Solar Water Splitting with Visible Light. Nano Lett. 2012, 12, 5014–5019. 10.1021/nl302796f. 22916955

[ref471] Li J.; Cushing S. K.; Zheng P.; Senty T.; Meng F.; Bristow A. D.; Manivannan A.; Wu N. Solar Hydrogen Generation by a CdS-Au-TiO_2_ Sandwich Nanorod Array Enhanced with Au Nanoparticle as Electron Relay and Plasmonic Photosensitizer. J. Am. Chem. Soc. 2014, 136, 8438–8449. 10.1021/ja503508g. 24836347

[ref472] Dutta A.; Naldoni A.; Malara F.; Govorov A. O.; Shalaev V. M.; Boltasseva A. Gap-Plasmon Enhanced Water Splitting with Ultrathin Hematite Films: The Role of Plasmonic-Based Light Trapping and Hot Electrons. Faraday Discuss. 2019, 214, 283–295. 10.1039/C8FD00148K. 30821797

[ref473] Kumar A.; Kumar S.; Rhim W.-K.; Kim G.-H.; Nam J.-M. Oxidative Nanopeeling Chemistry-Based Synthesis and Photodynamic and Photothermal Therapeutic Applications of Plasmonic Core-Petal Nanostructures. J. Am. Chem. Soc. 2014, 136, 16317–16325. 10.1021/ja5085699. 25386786

[ref474] Yang J.; Li Y.; Zu L.; Tong L.; Liu G.; Qin Y.; Shi D. Light-Concentrating Plasmonic Au Superstructures with Significantly Visible-Light-Enhanced Catalytic Performance. ACS Appl. Mater. Interfaces 2015, 7, 8200–8208. 10.1021/acsami.5b01078. 25840556

[ref475] Xie W.; Schlücker S. Hot Electron-Induced Reduction of Small Molecules on Photorecycling Metal Surfaces. Nat. Commun. 2015, 6, 7570 10.1038/ncomms8570. 26138619 PMC4506517

[ref476] Salmón-Gamboa J. U.; Romero-Gómez M.; Roth D. J.; Barber M. J.; Wang P.; Fairclough S. M.; Nasir M. E.; Krasavin A. V.; Dickson W.; Zayats A. V. Optimizing Hot Carrier Effects in Pt-Decorated Plasmonic Heterostructures. Faraday Discuss. 2019, 214, 387–397. 10.1039/C8FD00150B. 30801594

[ref477] Salmón-Gamboa J. U.; Romero-Gómez M.; Roth D. J.; Krasavin A. V.; Wang P.; Dickson W.; Zayats A. V. Rational Design of Bimetallic Photocatalysts Based on Plasmonically-Derived Hot Carriers. Nanoscale Adv. 2021, 3, 767–780. 10.1039/D0NA00728E. 36133839 PMC9419383

[ref478] Lock D.; Rusimova K. R.; Pan T. L.; Palmer R. E.; Sloan P. A. Atomically Resolved Real-Space Imaging of Hot Electron Dynamics. Nat. Commun. 2015, 6, 8365 10.1038/ncomms9365. 26387703 PMC4595757

[ref479] Rusimova K. R.; Bannister N.; Harrison P.; Lock D.; Crampin S.; Palmer R. E.; Sloan P. A. Initiating and Imaging the Coherent Surface Dynamics of Charge Carriers in Real Space. Nat. Commun. 2016, 7, 12839 10.1038/ncomms12839. 27677938 PMC5052722

[ref480] Wang P.; Nasir M. E.; Krasavin A. V.; Dickson W.; Zayats A. V. Optoelectronic Synapses Based on Hot-Electron-Induced Chemical Processes. Nano Lett. 2020, 20, 1536–1541. 10.1021/acs.nanolett.9b03871. 32013449

[ref481] Krasavin A. V.; Wang P.; Nasir M. E.; Jiang Y.; Zayats A. V. Tunneling-Induced Broadband and Tunable Optical Emission from Plasmonic Nanorod Metamaterials. Nanophotonics 2020, 9, 427–434. 10.1515/nanoph-2019-0411.

